# ReCHEMbinant stapling enhances intracellular delivery and bioactivity of engineered protein inhibitors

**DOI:** 10.1016/j.chempr.2025.102839

**Published:** 2026-04-09

**Authors:** Jan Pascal Kahler, Brecht D. Ellenbroek, Vera E. van der Noord, Bob van de Water, Sebastian J. Pomplun

**Affiliations:** 1 Leiden University, 2333 CC Leiden, the Netherlands; 2 Oncode Institute, 3521 AL Utrecht, the Netherlands

**Keywords:** protein engineering, stapling, MYC, synthetic proteins, novel chemical modalities

## Abstract

Protein therapeutics have transformed drug discovery by enabling modulation of challenging targets inaccessible to small molecules. However, most proteins lack the ability to penetrate cells, where many critical drug targets reside. Here, we present reCHEMbinant protein engineering, a strategy designed to generate synthetically enhanced proteins with improved structural stability, serum resistance, and cellular uptake. Applying this approach to Omomyc, a protein-based MYC inhibitor, we developed several reCHEMbinant stapled variants (HeloMYCs) exhibiting low-nanomolar DNA-binding affinity. Notably, the *i*, *i* + 7 biphenyl-stapled construct HeloMYC-1421 outperformed Omomyc across several functional assays, including potent inhibition of MYC-driven gene expression in luciferase reporter assays and selective antiproliferative effects in MYC-dependent cells. Live-cell imaging showed that these enhanced effects result from significantly improved cellular uptake. Transcriptional reprogramming was further confirmed by RNA sequencing (RNA-seq). Together, our findings establish reCHEMbinant engineering as a chemically defined strategy for stapling entire recombinant proteins to enhance their intracellular bioactivity.

## Highlights

• ReCHEMbinant engineering staples proteins for improved intracellular activity
• Stapled Omomyc variants, HeloMYCs, gain serum stability and cellular uptake
• HeloMYC-1421 shows enhanced MYC inhibition and blocks cancer cell proliferation
• The reCHEMbinant strategy broadens tools for engineering intracellular biologics

## The bigger picture

Proteins are the body’s natural machinery for carrying out highly specific biological functions, making them uniquely powerful as therapeutics. Unlike traditional small-molecule drugs, protein therapeutics can target large and complex biological surfaces that are otherwise “undruggable.” However, most protein drugs act only outside cells because they cannot efficiently cross cellular membranes. Many critical disease drivers, including oncogenic transcription factors (such as MYC), reside inside cells, making intracellular delivery of biologics one of the most important frontiers in modern drug discovery.

In this study, we developed reCHEMbinant engineering, a broadly applicable strategy that integrates recombinant protein production with site-selective chemical stapling to create synthetically enhanced proteins. Using this approach, we generated HeloMYCs, stapled versions of the MYC inhibitor Omomyc that show potent DNA binding, serum resistance, and markedly enhanced cellular uptake, translating into potent inhibition of MYC-driven cancer programs. Beyond MYC, reCHEMbinant engineering might establish a versatile framework for designing next-generation intracellular biologics. In the long term, this strategy could enable the development of stable, cell-permeable protein therapeutics that modulate previously inaccessible targets involved in cancer, neurodegeneration, and infectious diseases, supporting innovation in safer and more effective treatments for human disease.

## Graphical abstract

**Figure:**
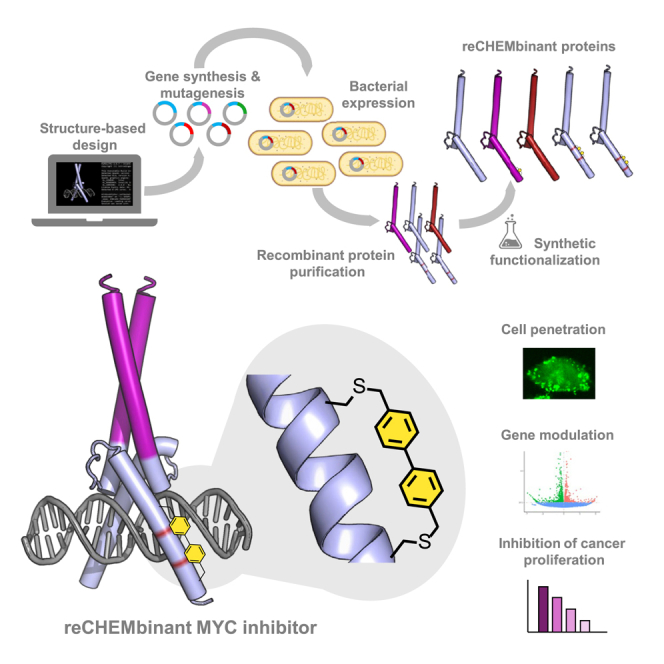

## Teaser

ReCHEMbinant engineering enables chemical stapling of recombinant proteins to enhance their cellular uptake, serum stability, and general activity. Applying this strategy to the MYC inhibitor Omomyc produced HeloMYCs—synthetically enhanced variants that display potent intracellular activity and better cell penetration and MYC inhibition than the natural parent protein.

## Introduction

Protein therapeutics have revolutionized drug discovery and expanded treatment options for challenging diseases.^1^ With the advent of recombinant technology, protein drugs have enabled the targeting of drug targets previously elusive for small molecules.^2^ Notable examples include antibodies such as Herceptin^3^ and Keytruda,^4^ as well as other therapeutic proteins, such as erythropoietin (EPO)^5^ and interferon.^6^ Because of their complex structures and extensive surfaces, protein drugs can recognize their targets with high specificity and potency. However, their large size and physicochemical properties prevent them from entering cells, making them effective primarily for extracellular targets. Many compelling drug targets, including thousands of protein-protein interactions (PPIs) and protein-nucleic acid interactions (PNAIs), do, however, reside inside cells, making them hardly accessible for protein-based drugs.^7^^,^^8^

Strategies that enable the direct cytosolic delivery of therapeutic proteins would have transformative potential for targeting intracellular pathways. Traditional approaches have relied on fusion to cell-penetrating peptides (CPPs), such as cyclic polyarginines, to facilitate uptake.^9^^,^^10^^,^^11^ These methods have enabled the delivery of cargos such as nanobodies^9^ and ubiquitin^10^ into cells, primarily in model systems. However, CPPs often exhibit nonspecific toxicity and poor serum stability, limiting their translational potential. An alternative approach developed by the Raines group involves transient masking of surface-exposed carboxylates as esters to promote protein uptake, demonstrated with GFP.^12^^,^^13^ Although conceptually elegant, this strategy is hampered by the serum lability of esters, which precludes therapeutic application. Other efforts have focused on miniaturizing protein domains into proteomimetic peptides that mimic key binding motifs.^14^^,^^15^^,^^16^^,^^17^^,^^18^ These constructs are often stabilized by chemical linkers or hydrocarbon staples and can achieve enhanced cell permeability thanks to their decreased size. However, replacing full-length proteins with short peptides can lead to reduced affinity or specificity, particularly when the target recognition depends on complex tertiary structures.

We sought to investigate a more general strategy for intracellular protein delivery by directly modifying full-length recombinant proteins. Our approach combines rationally introduced point mutations with compact, stable chemical modifications to enhance structural integrity and promote cell permeability without relying on large fusion tags or inherently toxic motifs.

For studies, we selected Omomyc, a well-characterized, dominant-negative variant of the transcription factor MYC.^19^^,^^20^^,^^21^^,^^22^^,^^23^^,^^24^ Omomyc forms homodimers that bind to the MYC recognition sequence (E-box) and competitively inhibit MYC’s oncogenic transcriptional activity. It offers several advantages as a model system for evaluating intracellular protein delivery strategies. First, Omomyc is biophysically well understood, and quantitative assays, such as DNA-binding electrophoretic mobility shift assays (EMSAs), enable precise evaluation of structural and functional integrity after chemical modification. Second, functional cellular readouts—including luciferase-based MYC reporter assays—provide rapid assessment of intracellular delivery and bioactivity. Third, Omomyc is recombinantly expressed in bacteria and is of moderate size, facilitating high-resolution analytical characterization, such as intact-protein mass spectrometry. Notably, Omomyc exhibits some intrinsic cell-penetrating activity^25^; however, conflicting reports suggest that its native uptake is limited and insufficient for robust therapeutic effects.^11^ These combined features—biochemical tractability, functional relevance, and borderline permeability—make Omomyc an ideal system for testing and optimizing chemically enhanced intracellular delivery strategies with potential therapeutic impact.

Here, we developed a reCHEMbinant protein engineering strategy that combines structure-guided design and recombinant expression of Omomyc analogs with targeted chemical modification. By introducing a series of sequence-based mutations and covalent stabilization elements, we generated stapled Omomyc variants with enhanced physicochemical and functional properties. This approach led to the identification of a reCHEMbinant class of compounds capable of efficient cellular uptake and potent inhibition of MYC-driven transcription. Our lead construct, HeloMYC-1421, exhibits low-nanomolar DNA-binding affinity and submicromolar potency in a MYC reporter assay, substantially outperforming unmodified Omomyc. Fluorescence imaging confirmed markedly improved cellular penetration, and further characterization demonstrated selective anti-proliferative effects in MYC-dependent cancer cells with no observable toxicity in MYC-independent lines. These findings establish reCHEMbinant stapling as a promising strategy for enabling intracellular delivery and therapeutic modulation of recombinant protein biologics.

## Results

### Design and synthesis of reCHEMbinant transcription factors

To engineer protein variants with high affinity for E-box DNA and improved bioactivity, particularly with a focus on cell penetration, we used Omomyc as a template. Our approach involved preserving its overall structural and functional characteristics while introducing targeted mutations to optimize bioactivity. We pursued two key strategies: (1) incorporating canonical sequence mutations to increase arginine content and (2) introducing chemical modifications aimed at enhancing structural stability and promoting cell penetration.

**Figure 1:**
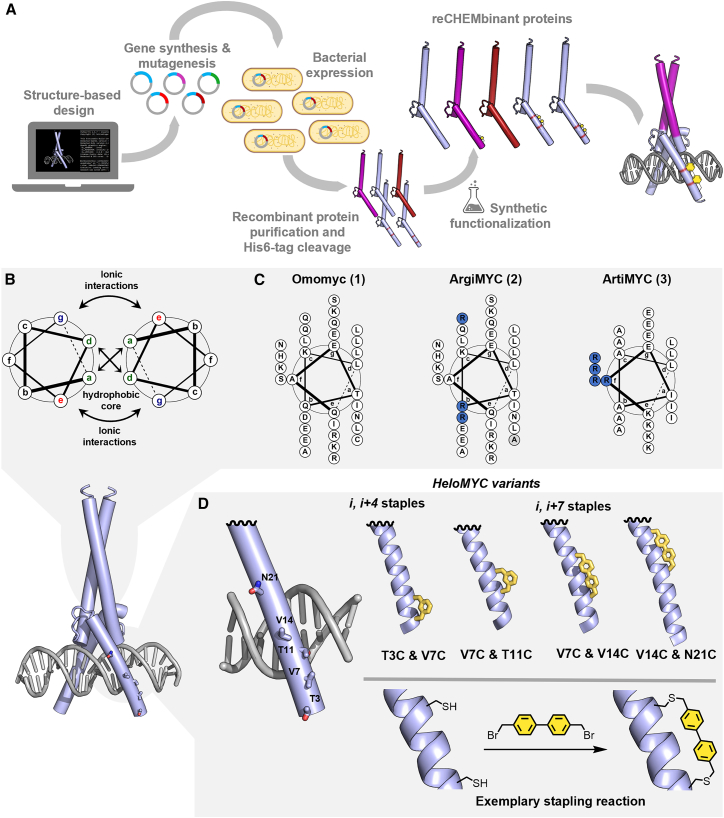
ReCHEMbinant protein design (A) Design, generation, and synthetic modification of reCHEMbinant proteins. After structure evaluation and mutagenesis, His-tagged proteins are expressed in *E. coli* cells. The His tag is then cleaved, and if applicable, the purified proteins are reacted with a staple. (B) Helical wheel showing the heptad pattern and the interactions between the α-helices in a coiled coil. (C) Helical wheel representation of the coiled-coil helices of Omomyc (**1**), ArgiMYC (**2**), and ArtiMYC (**3**). (D) DNA-binding basic part of Omomyc; residues that have been identified for mutation and stapling are highlighted. Two residues are mutated into Cys and react with a staple.

The cell-penetrating properties of polycationic arginine-rich peptides have been extensively reported.^26^ Studies about Omomyc’s intrinsic cell-penetrating properties have revealed that the arginines in the DNA-binding helix are critical for this ability.^25^ However, an excessive arginine content is associated with nonspecific toxicity.^27^^,^^28^^,^^29^ In an attempt to balance potential cell-penetrating efficacy and safety, we decided to modify the coiled-coil region of Omomyc by matching its arginine content to that of its DNA-binding helix. We first selected solvent-exposed residues, which, according to structural analysis, are not involved in the protein dimerization, and generated a variant—ArgiMYC—in which we mutated three residues (Q64, D71, and Q86) to arginine (Figure 1C, middle). In a second approach, we designed a fully artificial coiled coil (residues 61–83), also with higher arginine content than the wild-type protein (Figure 1C, right). The predicted AlphaFold structure of the resulting ArtiMYC dimer aligned well with wild-type Omomyc (Figure S1). ArgiMYC and ArtiMYC possess an overall slightly higher arginine content of 11.1% and 13.3%, respectively, than Omomyc (9.7%).

To chemically enhance the protein variants, we employed a semisynthetic strategy we refer to as the reCHEMbinant approach. Chemical staples, which stabilize the α-helical conformation of peptides, have been shown to improve cell penetration, stability, and overall bioactivity.^30^^,^^31^^,^^32^ These staples are covalent linkages between amino acid side chains spaced one or two helix turns apart (*i*, *i* + 4 or *i*, *i* + 7, respectively). The enhanced cell penetration of stapled helices is attributed to increased lipophilicity from the staple and the locked α-helical structure, which engages all backbone amides in intramolecular hydrogen bonding.^31^ In our reCHEMbinant approach, we adhered to the following steps: (1) identify suitable *i*, *i* + 4 and *i*, *i* + 7 positions on the basis of structural analysis; (2) mutate the selected residues pairwise to cysteines; (3) recombinantly express and purify the proteins; and (4) staple the proteins by using cysteine-reactive bifunctional reagents (Figure 1A).^33^^,^^34^ We selected four pairwise mutations—two for *i*, *i* + 4 and two for *i*, *i* + 7 stapling—and modified the backside of the DNA-binding helix to avoid disrupting DNA recognition (Figure 1D).

**Figure 2:**
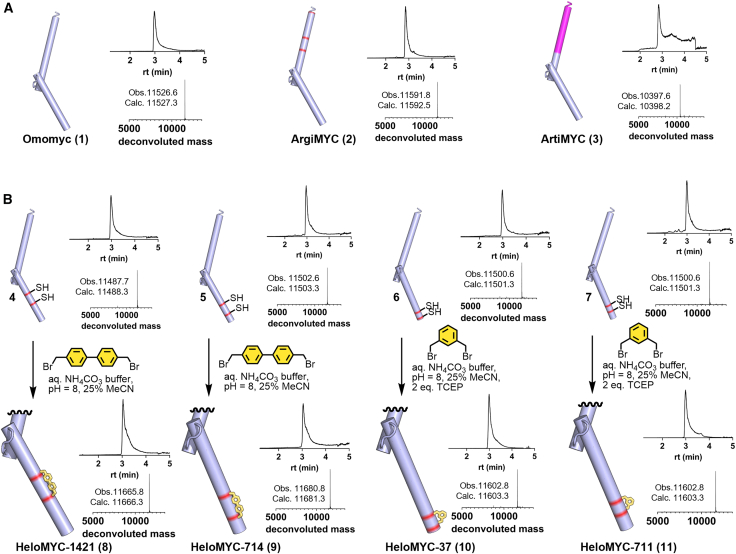
Generation of artificial transcription factors (A) Omomyc (**1**, left), coiled-coil modified Omomyc variants ArgiMYC (**2**), and ArtiMYC (**3**) were obtained by recombinant expression in good purity. (B) Point-mutated Omomyc derivatives obtained by recombinant expression in excellent purity (top) were reacted with the desired staple to yield HeloMYC proteins **8–11**.

We prepared all plasmids with the desired mutations and expressed the proteins in ArcticXpress cells. We purified via Ni-NTA affinity chromatography and subsequently cleaved the tags to release the pure protein products. Omomyc (1), ArgiMYC (2), and ArtiMYC (3) were all obtained in satisfying yield (8–14 mg/L culture) and purity, as shown by liquid chromatography-mass spectrometry (LC-MS) (Figures 2A and 2B). These three variants (1, 2, and 3) were not further synthetically modified. We also expressed and purified the bis-cysteine variants and further modified the proteins with either 4,4′-bis(bromomethyl)-1,1′-biphenyl or 1,3-bis(bromomethyl)benzene. The *i*, *i* + 4 cysteine pairs required the addition of a reducing agent (TCEP) during the reaction to prevent disulfide formation. All site-specific protein functionalizations resulted in clean conversions, and we obtained the four reCHEMbinant stapled proteins: HeloMYC-37, -711, -714, and -1421 (Figures 2C, S14, and S15).

### Biophysical characterization and binding evaluation

**Figure 3:**
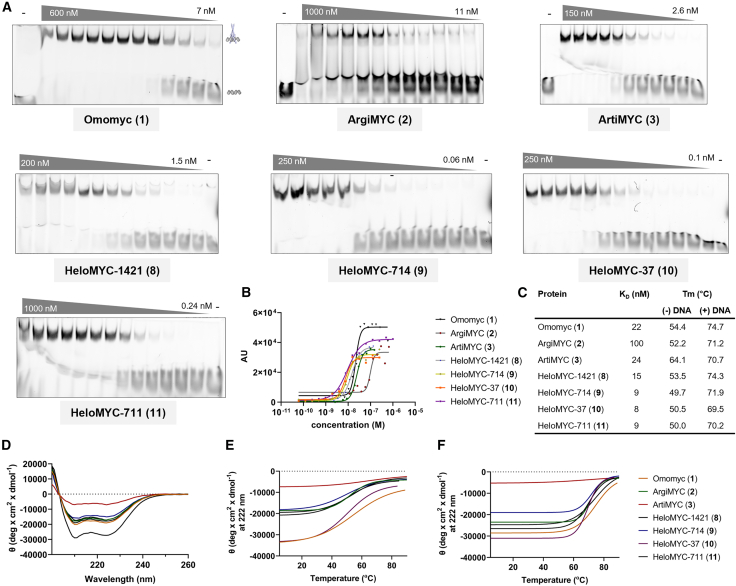
ReCHEMbinant transcription factors bind to E-box DNA with high potency (A) EMSAs of ReCHEMbinant transcription factors with 5′-IRD700-labeled dsDNA (sequence 5′-IRD700-ACCCCACCACGTGGTGCCT–3′) show DNA binding of Omomyc (**1**), ArgiMYC (**2**), ArtiMYC (**3**), and HeloMYC variants (**8–11**). The DNA construct is incubated with a miniprotein, and the mixture is resolved by native gel electrophoresis. DNA binding is seen as the DNA-protein complex running higher than free DNA. (B) Plot of curves used for K_D_-value determination. AU values were obtained via quantification of the signal of the bound fraction of DNA. (C) Summary of all K_D_ values as determined by EMSA and structural melting points as determined by CD in the absence and presence of E-box DNA. (D) CD spectra of all constructs. (E and F) CD melting curves of all constructs in the absence (E) and presence (F) of E-box DNA, measured at 222 nm.

We measured the affinity to E-box DNA of all reCHEMbinant transcription factors via EMSA. By design, all of our engineered protein variants are supposed to homodimerize, bind to E-box DNA, and result in a detectable shift when measured via native gel electrophoresis. To visualize the DNA and the protein-DNA complexes, we incubated all variants with 5′-IRD700-labeled dsDNA containing the E-box sequence CACGTG and resolved the mixture on a native gel. We then quantified the bound fraction of DNA and calculated K_D_ values. The control protein Omomyc bound DNA with a K_D_ of 22 nM. ArgiMYC (**2**) showed a K_D_ of 100 nM but consistently resulted in smeared bands, potentially indicating partial unspecific binding. ArtiMYC (**3**) bound E-box with 24 nM, an affinity comparable to that of Omomyc. The HeloMYC variants resulted in K_D_ values between 15 and 8 nM, representing a 2- to 3-fold better affinity than that of their unmodified parent compound Omomyc (Figures 3A–3C and S5–S11).

We used circular dichroism (CD) to evaluate structural features and structural stability of all variants. CD spectra of all proteins exhibited defined minima at around 208 and 222 nm, indicating α-helical folding (Figure 3D). Only ArtiMYC (**3**) showed a weaker CD signal, albeit still indicating α-helicity. This effect might have resulted from aggregation, but we did not investigate this phenomenon further. We also performed CD melting experiments in the absence and presence of DNA. In all cases, the presence of DNA resulted in significant structural stabilization (approximately +20°C), further confirming the functional DNA binding of these variants.

Together, our set of biophysical assays indicate robust DNA-binding activity and α-helical folding for the four stapled HeloMYC variants (**8–11**). ArgiMYC and ArtiMYC showed less-convincing results in the EMSA assay and CD analysis, respectively.

### Evaluation of bioactivity

**Figure 4:**
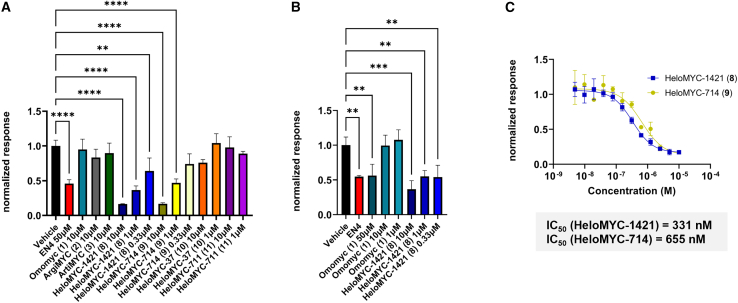
Biphenyl-stapled HeloMYC miniproteins significantly reduce MYC-related gene transcription in a reporter assay (A and B) HEK293T (A) and HeLa (B) cells transiently transfected with a MYC-dependent luciferase gene were incubated with transcriptional repressor miniproteins or small-molecule MYC inhibitor EN4 for 1 day, and luciferase activity was measured subsequently. HeloMYC-714 and HeloMYC-1421 show significant downregulation of MYC-related luciferase expression. (C) IC_50_ values for MYC-regulated luciferase expression inhibition by HeloMYC-1421 and HeloMYC-714 were measured in transiently transfected HEK293T cells. A one-way ANOVA comparing the effect of miniprotein treatment on normalized signal showed that there is a statistical difference between treatments: (A) F (14, 30) = 22.76, *p* < 0.0001; (B) F (7, 16) = 13.21, *p* < 0.0001. ∗ *p* < 0.05, ∗∗ *p* < 0.01, ∗∗∗ *p* < 0.001.

To evaluate the intracellular effects of our reCHEMbinant protein variants, we performed MYC-responsive reporter gene assays, which revealed that only the *i*, *i* + 7-stapled constructs HeloMYC-714 and HeloMYC-1421 elicited significant cellular activity. These assays used a firefly luciferase gene activated by MYC/MAX dimers with a constitutively expressed *Renilla* luciferase under control of the CMV promoter for normalization. After transfection of HEK293T with dual reporter DNA, cells were treated for 24 h with Omomyc or our mutated reCHEMbinant transcription factors. We also included the small-molecule covalent MYC inhibitor EN4 as a positive control.^35^ On the basis of previous reports, the main expected mechanism of action of Omomyc and analogs would be forming dimers and occupying the MYC/MAX DNA-binding sites.^25^ Among all protein variants, only the *i*, *i* + 7-stapled proteins HeloMYC-714 and -1421 displayed significant downregulation of MYC reporter activity (Figure 4A). Both HeloMYC derivatives exhibited roughly the same effect on MYC activity at 1 μM concentration as EN4 did at 50 μM. We then went on to measure their EC_50_ values in the reporter gene assay, which we determined to be 655 nM for HeloMYC-714 and 331 nM for HeloMYC-1421 (Figure 4C; data from three independent experiments in Figure S2). To corroborate these findings, we tested a subset of compounds (HeloMYC-1421 [**8**], Omomyc [**1**], and EN4) in HeLa cells, where HeloMYC-1421 (**8**) again showed potent reporter downregulation at sub-micromolar concentrations, whereas Omomyc (**1**) was effective only at 50 μM (Figure 4B). In our control experiments, we confirmed that the constructs had no impact on the constitutively expressed CMV-driven *Renilla* luciferase but that HeloMYC-1421 and EN4 exhibited a negative effect on constitutively expressed CMV-driven firefly luciferase. We could experimentally exclude that these compounds act as direct enzymatic inhibitors by adding the constructs to control-transfected cells directly before cell lysis and luciferase readout. In addition to suggesting direct MYC inhibition, these experiments therefore hint at a likely more complex inhibition pathway mechanism (Figure S3 for validation data). Despite this uncertainty, the MYC reporter gene assays indicated strong intracellular activity for the *i*, *i* + 7 biphenyl-stapled proteins HeloMYC-714 and HeloMYC-1421, whereas all other variants, including Omomyc, showed little to no effects.

To investigate whether the improved activity of the HeloMYC variants in the reporter gene assay was a result of improved cell penetration, we turned to fluorescence microscopy. Given that protein variants **2** and **3** (with increased arginine content) and the variants with the *i*, *i* + 4 staples had not shown any cell activity, we proceeded with only analyzing HeloMYC-1421 and comparing it against the unmodified parent protein Omomyc.

**Figure 5:**
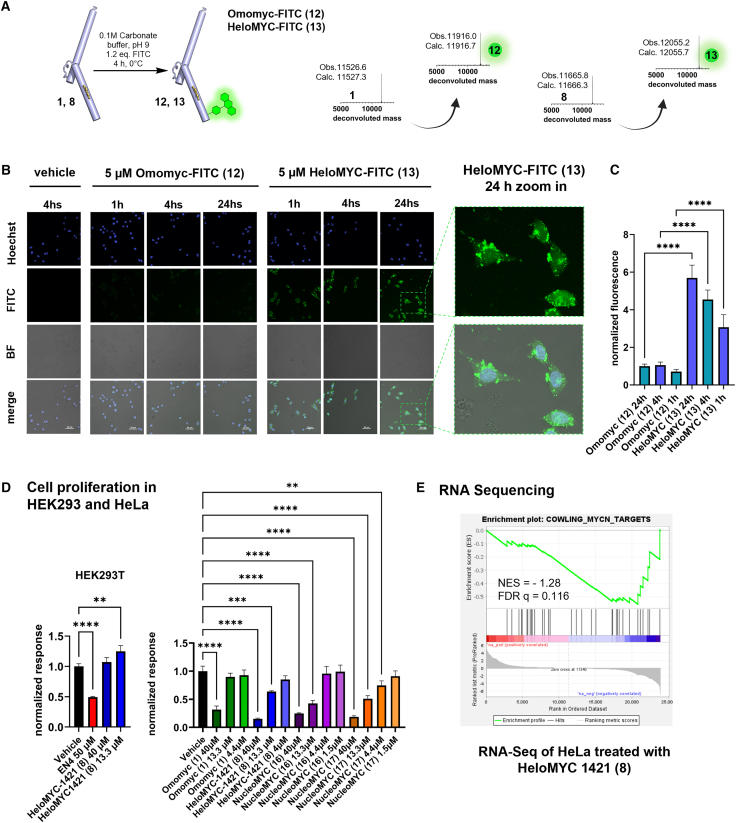
HeloMYC-1421 is cell permeable and inhibits cell proliferation of cancer cell lines (A) Omomyc (**1**) and HeloMYC-1421 (**8**) were reacted with FITC in carbonate buffer. (B) Fluorescence microscopy of live HeLa cells treated with HeloMYC-FITC (**13**) or Omomyc-FITC (**12**) and imaged at 1, 4, and 24 h. (C) Quantification of fluorescence shows superior cell permeability of HeloMYC-FITC (**13**). (D) Cell titer glow (CTG) assays for assessing HeloMYC-1421 (**8**) activity proliferation of HEK293T cells and CTG assay in HeLa cells with HeloMYC-1421 (**8**) and NucleoMYC **16** and **17**. (E) RNA-seq: HeLa cells were treated with HeloMYC-1421 (**8**) at 10 μM for 3 days. RNA was extracted and sequenced. The GSEA plot for the COWLING_MYC_Targets is shown. A one-way ANOVA comparing the effect of miniprotein treatment on normalized signal showed that there is a statistical difference between treatments: (B) F (5, 186) = 132.8, *p* < 0.0001; (D) HEK293T F (3, 8) = 75.49, *p* < 0.0001; HeLa F (14, 28) = 49.17, *p* < 0.0001. ∗ *p* < 0.05, ∗∗ *p* < 0.01, ∗∗∗ *p* < 0.001.

Fluorescence imaging revealed that HeloMYC-1421 entered cells more efficiently than Omomyc. We labeled HeloMYC-1421 and Omomyc with fluorescein isothiocyanate (FITC) and purified the resulting variants **12** and **13** (Figures 5A, S18, and S19). We then performed live-cell imaging in HeLa cells upon incubation with the fluorescent proteins (at 5 μM) for 1, 4, or 24 h. At all time points, HeloMYC-FITC (**13**) was taken up into cells significantly more than Omomyc-FITC (**12**). We observed that the uptake increased over time and also enhanced colocalization with the nucleus (Figure 5C). This time course points toward an endocytosis-based cell-entry mechanism followed by partial endosomal escape. Figure 5B shows that part of the compound remained localized in endosomal structures (green puncta), whereas a substantial fraction was also diffusely distributed throughout the cytosol and nucleus. The more-efficient cell penetration than that of Omomyc most likely explains the enhanced efficacy of the HeloMYC variants in the reporter gene assay.

Because α-helix stapling is often associated with improved resistance against proteases, we also performed a serum stability assay to compare unmodified Omomyc (**1**) with HeloMYC-1421 (**8**). We incubated both proteins in 10% human serum and assessed their half-lives by LC-MS (Figure S19). HeloMYC-1421 (**8**) displayed significantly better stability than Omomyc (4.8 vs. 1.3 h). In addition to the better cell uptake, the stability can also partly explain the improved bioactivity.

HeloMYC-1421 (**8**) inhibited the proliferation of cancer cells without inducing nonspecific cytotoxicity in MYC-independent cells. To assess potential off-target toxicity, we treated HEK293T cells—which do not rely on MYC for survival—with HeloMYC-1421 (**8**). The cells tolerated treatment well and showed no loss of viability after 3 days at concentrations up to 40 μM (Figure 5D). By contrast, MYC-dependent HeLa cells showed a significant reduction in proliferation at concentrations as low as 13.3 μM. To enhance nuclear delivery, we generated HeloMYC-1421 variants bearing nuclear localization sequences (NLSs) at either the N or C terminus (NucleoMYC **16** or **17**, respectively; Figures S4 and S12–S14). NucleoMYC **17** showed a modest improvement in potency, although overall, the NLS addition had only a moderate effect. Collectively, these data show that HeloMYC-1421 and its NLS-tagged variants inhibit cancer cell proliferation at 13.3 μM (compounds **8** and **16**) and 4.4 μM (compound **17**), and importantly, the reCHEMbinant HeloMYC did not exhibit cytotoxicity in MYC-independent HEK293T cells.

HeloMYC-1421 (**8**) modulates MYC activity in HeLa cells, leading to significant transcriptional changes. After 3 days of treatment, RNA sequencing (RNA-seq) analysis revealed 712 upregulated and 820 downregulated genes. Gene-set enrichment analysis (GSEA) uncovered a trend toward coordinated downregulation of the Cowling MYC target gene set, reflecting partial suppression of MYC-driven transcription (Figures 5E and S22). Although the enrichment was modest, this subtle effect aligns with the expected mechanism of the inhibitor, which tempers MYC function rather than fully abolishing it, unlike the complete loss seen in knockout models used for the gene-set comparisons. This nuanced modulation supports the therapeutic potential of HeloMYC-1421.

**Figure 6:**
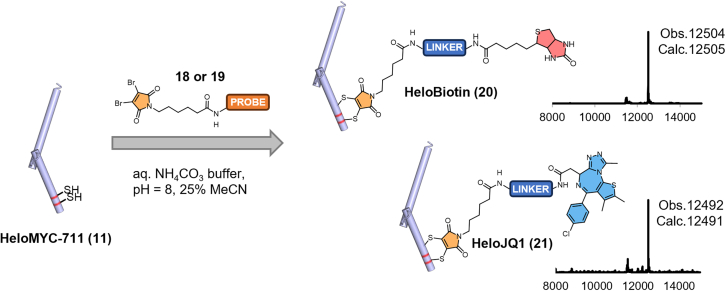
Synthesis of reCHEMbinant protein conjugates The reaction between HeloMYC-711 (**11**) and trifunctional dibromomaleimide-biotin (18) or dibromomaleimide-JQ1 (19) resulted in efficient conversion to HeloBiotin (20) and HeloJQ1 (21).

As a final experiment, we explored whether the stapling position in reCHEMbinant transcription factors could also serve as a site for the conjugation of additional functional groups, such as small molecules or affinity handles. To this end, we designed and synthesized two trifunctional probes containing a dibromomaleimide moiety for stapling via two cysteines and incorporated an additional functionality through substitution at the maleimide nitrogen (Figures S20 and S21). Probe 18 carried a biotin handle, whereas probe 19 contained the BET inhibitor JQ1, a compound known to modulate MYC expression. HeloMYC-711 (**11**) was reacted with these probes in aqueous buffer, resulting in efficient conversion to the corresponding conjugates, HeloBiotin and HeloJQ1 (Figure 6). Although we did not pursue a detailed characterization of these conjugates, the straightforward synthetic strategy demonstrates a feasible route for generating chemically diversified protein variants that could be of use in future biochemical studies or for probing functional effects.

## Discussion

In this study, we developed a chemically enhanced protein engineering strategy targeting the nuclear DNA-binding site of the oncogenic transcription factor MYC. Efficient intracellular delivery of proteins remains a key challenge for protein therapeutics.^9^^,^^10^^,^^12^^,^^13^^,^^36^ Starting from Omomyc, a known MYC inhibitor with some intrinsic cell-penetrating properties, we explored two distinct strategies to improve cell permeability and bioactivity: (1) increasing the arginine content, inspired by polycationic CPPs, and (2) chemically modifying the DNA-binding domains with peptide staples.

Whereas the arginine-rich variants (ArgiMYC and ArtiMYC) and the *i*, *i* + 4 staples showed no detectable improvements in cell activity, the *i*, *i* + 7 stapled proteins (HeloMYC-714 and HeloMYC-1421) exhibited significantly enhanced cellular activity. Via fluorescent microscopy, we confirmed that the biphenyl staple leads to a substantially improved uptake in cells. Interestingly, despite minimal impact on helical stability, the *i*, *i* + 7 stapling led to pronounced gains in cellular uptake, potentially as a result of the amphipathic nature of these constructs. The *i*, *i* + 4 xylene staple might not add enough hydrophobicity to the helix backside to achieve the same effect. Also, just slightly increasing the arginine content, as attempted in ArgiMYC and ArtiMYC, did not lead to the desired enhanced cellular uptake. Three more arginines distributed along such a long sequence might not be enough for such activity. Although arginine-rich peptides are often cell penetrating, the arginine residues might require closer clustering as opposed to the distributed placement in ArgiMYC and ArtiMYC.

Our findings highlight the unique potential of protein stapling for generating cell-penetrant protein drugs targeting intracellular complexes. Although peptide staples have been used for stabilizing α-helical structures, protecting against proteolytic degradation, and improving cell permeability,^31^^,^^32^^,^^37^ the application of stapling techniques to entire recombinant proteins, aimed at these properties, to date remains largely underexplored. Protein stapling has been reported but mainly in proof-of-principle studies or aimed at tertiary structure stabilization.^38^^,^^39^^,^^40^^,^^41^ Although there have been previous efforts to synthesize MYC analogs,^16^^,^^17^^,^^24^^,^^42^^,^^43^^,^^44^^,^^45^^,^^46^^,^^47^ we developed a practical reCHEMbinant workflow for directly modifying recombinant proteins. Our reCHEMbinant approach, which combines recombinant protein expression with targeted cysteine modification, enables rapid generation of stapled protein variants and could be applicable to other α-helical protein domains. Recent technology breakthroughs enable the *de novo* design of miniproteins binding to virtually any target.^48^ For intracellular targets, these approaches are of limited interest, but our reCHEMbinant strategy could unlock this potential.

Our cell-proliferation experiments showed that HeloMYC inhibits the proliferation of cancer cells without affecting MYC-independent HEK293 cells, indicating that it lacks the unspecific membrane toxicity sometimes observed for CPPs.^29^ Although our HeloMYC variants were clearly superior to the parent compound Omomyc, their current potency in inhibiting cell proliferation suggests that fully realizing their clinical potential will require further optimization. Overall, our findings demonstrate that this approach can significantly enhance the therapeutic potential of protein drugs, expanding their applicability to previously undruggable intracellular targets.

## Methods

### Expression of different Omomyc variants

Plasmids for the stapled HeloMYC variants were generated by site-directed mutagenesis of pET-30a with the Omomyc gene sequence. Plasmids for the ArtiMYC and ArgiMYC coiled-coil constructs and the NLS-HeloMYC fusions were supplied and express closed by GenScript and used as delivered (see the supplemental information for plasmid sequences). Plasmids were transformed into competent ArcticXpress DE3 RIL cells by heat shock according to the manufacturer’s protocol. In brief, 2 μL of 10% β mercaptoethanol was mixed with 100 μL of competent cell suspension thawed on ice and incubated for 10 min on ice. Next, 25 ng of plasmid DNA was added, and the cells were incubated for another 30 min on ice. The cells were then heat-shocked in a water bath for 20 s at 42°C and subsequently incubated on ice for 2 min. Then 0.9 mL SOC media was added, and the cells were incubated at 37°C and 250 rpm for 1 h. The cells were then pelleted by centrifugation, 0.9 mL of the supernatant was decanted, and the pellet was resuspended in the remaining 100 μL of media. The cells were plated for selection on LB agar with kanamycin and gentamicin and incubated at 37°C overnight.

Single colonies were picked from the plate and cultured overnight at 37°C and 180 rpm in 100 mL of LB media containing kanamycin and gentamycin. Next, 3 × 2 L of LB media containing kanamycin and gentamycin in 5 L Erlenmeyer flasks were inoculated with 25 mL of preculture and grown at 37°C and 180 rpm until an OD of 0.8 was reached. The temperature was then set to 14°C, and protein expression was induced by the addition of IPTG to a final concentration of 100 μM. Protein expression was conducted overnight for 18 h, after which the cells were harvested by centrifugation (5,000 g, 4°C, 12 min), the pellet was resuspended in lysis buffer (20 mM Tris-HCl [pH 8], 0.5 M NaCl, 10 mM imidazole, 3 mM MgCl_2_, one cOmplete EDTA-free Protease Inhibitor Cocktail tablet per 50 mL of buffer, 0.05%–0.1% DNase), and the cells were lysed by pressure lysis. Cell debris was removed by ultracentrifugation (35,000 rpm, 4°C, 45 min).

### Purification of His-tagged proteins

After ultracentrifugation, the supernatant was purified using an ÄKTA start protein purification system equipped with a 5 mL HisTrap HP His-tag protein purification column (Cytiva). After unbound protein was washed out with wash buffer (20 mM Tris-HCl [pH 8], 0.5 M NaCl, 10 mM imidazole), the protein was eluted with a gradient of 10–500 mM imidazole in the same buffer over 40–50 column volumes of buffer. Fractions with protein were analyzed for protein content and identity by LC-MS.

### General protocol for buffer exchange

The combined fractions obtained from Ni-column purification were incubated with 5 mM TCEP for 1 h to break any possible formed disulfide bonds. We then exchanged the buffer by subjecting the protein to column chromatography on a Biotage Selekt Flash Purification System equipped with a Biotage Sfär C18 D-Duo 25 or 50 g column and a stepwise gradient of 0% MeCN in water followed by 50%–100% MeCN in water. The combined fractions containing the protein were lyophilized, yielding the His-tagged or final protein as TFA salt.

### Tag cleavage with enterokinase and subsequent purification

Lyophilized protein was dissolved at 2 mg/mL in EK cleavage buffer (200 mM Tris-HCl [pH 7.4], 0.5 M NaCl, 20 mM CaCl_2_), and after the addition of 10 u/mL enterokinase, the protein was incubated overnight. The protein was then purified on an ÄKTA start protein purification system equipped with a 5 mL HisTrap HP His-tag protein purification column (Cytiva) and a gradient of 10–500 mM imidazole in elution buffer (20 mM Tris-HCl [pH 8], 0.5 M NaCl, 10–500 mM imidazole). The fractions were analyzed by LC-MS, and the buffer of the combined protein-containing fractions was exchanged as described above, yielding the final proteins as TFA salts after lyophilization.

### Site-directed mutagenesis

For site-directed mutagenesis, the QuikChange II Site-Directed Mutagenesis Kit (Agilent Technologies) was used, and primers were designed with the QuikChange Primer Design Program provided by Agilent Technologies. Mutagenesis was performed according to the manufacturer’s protocol. In brief, PCRs were prepared with pET-30a with Omomyc as a template (0.5 μL, ∼25 ng), forward and reverse primers (0.6 μL each, 10 μM), MQ water (18.8 μL), and the contents provided by the kit: NTP mix (1 μL), 10× reaction buffer (2.5 μL), and *PfuUltra* High-Fidelity DNA Polymerase. In some cases, when the formation of primer dimers was seen, primer concentration was reduced, and 1 μL of DMSO was added. PCR was performed for 22 cycles (95°C, 30 s; 55°C, 1 min; 68°C, 10 min), and then DpnI restriction was performed for 2 h at 37°C (addition of 0.5 μL at 10 U/μL).

Mutated plasmids were then incorporated into XL-1 blue competent cells. Cells were thawed on ice, and 8 μL of the DpnI-treated DNA was added to 50 μL of cells. The mixture was incubated on ice for 30 min, subjected to a 45 s heat pulse at 42°C, and incubated on ice for another 2 min. Subsequently, 0.5 mL SOC medium was added, and the cells were shaken at 250 rpm at 37°C for 1 h. For selection, 100 μL of the mixture was plated on one half of an LB agar plate containing gentamicin and kanamycin. The rest of the cells were spun down, the supernatant was removed (except for 100 μL), and the cells were resuspended in this 100 μL and plated on the other half of the LB agar plate. The plate was incubated at 37°C overnight.

About five to ten colonies were picked from selection and grown overnight in 5 mL LB media supplemented with gentamicin and kanamycin, and plasmid DNA was isolated with the QIAprep Spin Miniprep Kit (QIAGEN) according to the manufacturer’s protocol. The isolated plasmid DNA was sequenced by a Sanger sequencing service and analyzed with Benchling.

### Stapling reactions

#### Stapling with the *i*, *i* + 7 staple (4,4′-bis(bromomethyl)biphenyl)

The respective protein (1 equiv **4** or **5**) was dissolved in water (2 mM, e.g., 30 mg of **5** in 1.08 mL) and then diluted to 100 μM into stapling buffer (15.12 mL for **5**, NH_4_HCO_3_, 100 mM [pH 8]). 4,4′-Bis(bromomethyl)biphenyl was dissolved at 4× the final concentration in MeCN (500 μM) and then added (1.25 equiv, 5.4 mL for HeloMYC-1421) to the protein in stapling buffer. The final concentrations were 100 μM (1 equiv) protein and 125 μM (1.25 equiv) staple. The final MeCN content was 25%.

When the stapling reaction was completed as seen by LC-MS, the product was purified with a Biotage Selekt Flash Purification System equipped with a Biotage Sfär C18 D-Duo 10 g column on a gradient of water in MeCN (0%–100% with a flat gradient between 15% and 79%), both containing 0.1% TFA. The product containing fractions was lyophilized, yielding the proteins as TFA salts.

#### Stapling with the *i*, *i* + 4 staple (α,α′-dibromo-m-xylene)

The respective protein (1 equiv **6** or **7**) was dissolved in water (to 2 mM) and then diluted into stapling buffer (NH_4_HCO_3_, 100 mM [pH 8]). TCEP was dissolved at 100× the final concentration in water (20 mM) and added to the stapling reaction (2 equiv, 14.1 μL). α,α′-Dibromo-m-xylene was dissolved at 4× the final concentration in MeCN (500 μM) and then added (1.25 equiv, 353 μL for HeloMYC-711) to the protein in stapling buffer. The final concentrations were 100 μM (1 equiv) protein, 125 μM (1.25 equiv) staple, and 200 μM (2 equiv) TCEP. The final MeCN content was 25%.

When the stapling reaction was completed as seen by LC-MS, the product was purified with a Biotage Selekt Flash Purification System equipped with a Biotage Sfär C18 D-Duo 10 g column on a gradient of water in MeCN (0%–100% with a flat gradient between 15% and 79%), both containing 0.1% TFA. The product containing fractions was lyophilized, yielding the proteins as TFA salts.

#### EMSA

For EMSAs, proteins were serially diluted with water to a final volume of 10 μL. Subsequently, 5 μL of 4× EMSA buffer (final buffer concentration: 20 mM HEPES [pH 8.0], 150 mM NaCl, 5% glycerol, 1 mM EDTA, 2 mM MgCl_2_, 0.5 mg/mL of BSA, 1 mM DTT, 0.05% NP-40) followed by 5 μL of 4× FAM-labeled DNA construct (IRD700-ACC CCA CCA CGT GGT GCC T, final concentration 4 nM) was added.

The samples were incubated for 30 min at room temperature (RT), placed on ice, and incubated for another 15 min. Then 15 μL of the samples was loaded onto a 10% native acrylamide TBE gel, which was pre-run before for 1 h at 75 V at 4°C in 0.5× TBE. Samples were run for 20 min at 120 V followed by 40 min at 100 V at 4°C in 0.5× TBE and subsequently scanned on a Bio-Rad ChemiDoc MP machine. Bound protein signal was quantified with ImageJ, and K_D_ values were obtained with the inhibitor concentration vs. response variable slope model of nonlinear regression in GraphPad Prism 9.0.0. (the IC_50_ value was reported as K_D_).

#### CD spectroscopy

For CD measurements, 1 mM protein stocks were diluted to 20 μM in Dulbecco’s phosphate buffered saline (DPBS) to a final volume of 200 μL. For DNA-containing samples, a 1 mM DNA stock in water was heated for 5 min to 95°C and left to cool down to RT, and an equimolar amount of DNA was added to the protein samples to be measured with DNA. Additionally, a blank measurement with or without DNA was taken. CD samples were measured at 37°C with a Jasco J-8151 CD spectrometer with a 1-mm-path-length quartz cuvette. The following parameters were used for a full wavelength scan: wavelength = 260–200 nm; data pitch = 1 nm; scanning mode = continuous; scanning speed = 100 nm/min; response = 1, BW = 1, accumulation = 5. For CD melting curves, the same samples were cooled down to 5°C and slowly heated to 90°C while being measured at 222 nm with a heating speed of 2°C/min.

For CD analysis, the blank measurement was subtracted from each spectrum, and the mean residue molar ellipticity (*θ*, deg cm^2^ dmol) was calculated with the following equation^49^:

$$\left[\theta \right]=\frac{100*\theta_{\text{obs}}}{c*n*l}$$

with *θ*_obs_ in mdeg, concentration (*c*) in mM, and peptide bonds (*n*) and the path length of cuvette in cm.

#### Serum stability assay

For serum stability assays, 1 mM protein stocks were diluted to a final concentration of 60 μM in 10% human serum in DPBS. The mixture was vortexed immediately after protein addition, and 5 μL aliquots were mixed with 5 μL of 20% TFA in water (t = 0) to quench the human serum, resulting in protein precipitating. Subsequently, the protein serum solution was incubated at 37°C with a BIO-RAD T100 Thermal Cycler with the lid set to 95°C. At the indicated time points, 5 μL aliquots were mixed with 5 μL of 20% TFA in water. The resulting pellet was then diluted 5.5× with additional DPBS to redissolve. This solution was analyzed according to the high-resolution LC-MS protocol (supplemental method C). As an internal standard (IS), the extracted-ion chromatogram (XIC) from *m*/*z* = 1233.52, belonging to human serum albumin, was used. After measurement, the XICs obtained from the highest intensity peak belonging to each protein and IS were extracted from the total-ion chromatogram (TIC). With GraphPad Prism 9, the area under the curve (AUC) from each time point was calculated, normalized against the AUC of the IS and then against t = 0, and plotted. Next, a nonlinear regression—one phase decay analysis—was performed to yield t_½_ with a plateau constant equal to 0 and Y_0_ set to 1. The assay was performed in triplicate. For Omomyc, one set of outliers in the measurement was excluded.

#### Cell culture and cell assays

Cells were cultured at 37°C in a 5% CO_2_ atmosphere. Cell lines were cultured in American Type Culture Collection (ATCC)-recommended medium and split twice a week before confluency was reached.

#### MYC reporter gene assay

The reporter gene assay was performed with the Cignal Reporter Assay Kit (CCS-012L, QIAGEN). In brief, HEK293T cells were harvested and resuspended in OptiMEM media containing 5% fetal bovine serum (FBS) and 1% non-essential amino acids (NEAAs), as well as penicillin/streptavidin. 40,000 cells were seeded per well in a 96-well plate; 50 μL of transfection cocktail of either the Cignal reporter or the positive or negative control reporter, along with the attractene transfection reagent in OptiMEM without additives, was added; and the cells were incubated overnight. Next, the medium was changed to assay medium (OptiMEM, 0.5% FBS, 1% NEAA, penicillin/streptavidin), and the cells were incubated for 8 h, after which the medium was replaced with 75 μL assay medium containing the different proteins at the required concentration and the cells were incubated with the proteins for 24 h.

Luciferase assay was then performed with a luciferase assay kit (E2940, Promega). Cells were lysed by addition of 75 μL of DualGlo luciferase assay reagent and incubated for 15 min, after which luciferase luminescence was measured on a Perkin-Elmer EnVision 2104 Multilabel Reader. Subsequently, 75 μL of DualGlo Stop & Glo reagent was added, and *Renilla* luciferase luminescence was measured after 15 min of incubation time.

We normalized signal against cell number by calculating the ratio of firefly and *Renilla* luminescence, and eventually these signals were normalized against the untreated control. All experiments were done in technical triplicates. Data were analyzed with GraphPad Prism 9.0.0. with the model of inhibitor concentration vs. response with the variable slope model for nonlinear regression.

#### Cell-proliferation assay

Cells were seeded in 100 μL of their respective media at 1,500 cells/well (Figure 5D) or 2,000 cells/well (Figure 5E) in a white opaque 96-well plate and left to attach overnight. The medium was then changed to 100 μL of medium with the required protein at the appropriate concentration, the plate was covered with a membrane to prevent medium evaporation, and the cells were incubated with the proteins for 72 h. Cell proliferation was then assessed with CellTiter-Glo (Promega) reading luminescence on a Perkin-Elmer EnVision 2104 Multilabel Reader. All experiments were done in technical triplicates. Data were analyzed with GraphPad Prism 9.0.0.

#### Live-cell microscopy with FITC-labeled HeloMYC and Omomyc

HeLa cells were harvested and seeded at 12,000 cells/well in a 96-well plate and left to adhere overnight (4.5 h in the case of 24 h compound treatment). Cells were then treated with 5 μM Omomyc-FITC (12) or HeloMYC-1421-FITC (13) for the desired time in full medium. Subsequently, the medium was aspirated, and the cells were stained for 10 min with 1 μg/mL Hoechst in DPBS and then washed three times with full medium. Live cells were imaged on a Nikon Ti2 microscope equipped with a Plan Apo VC 20× DIC N2 air objective with a 405 nm laser for Hoechst and a 488 nm laser for fluorescein excitation.

#### Quantification of signal in the green channel

For signal quantification, the Hoechst channel was used, and regions of interest (ROIs) were determined via thresholding from 100 to 255. The ROIs were then dilated by 5 units to also cover the area surrounding the nucleus, and a mask was created. The mask was applied to the FITC channel, and the mean fluorescence was measured per image. For each condition, the mean of the mean fluorescence of the no-treatment control was subtracted from the individual mean fluorescence, and the values were normalized to the desired condition.

#### RNA-seq and GSEA

In a 12-well plate, 50,000 HeLa cells/well were seeded. The next day, the cells were treated with either 10 μM HeloMYC-1421 or vehicle in MEM medium supplemented with 10% FBS, 1% Pen/Strep, and Glutamax and incubated for 72 h. The total RNA was isolated with the QIAGEN RNeasy Plus Mini Kit and dissolved in RNase-free water. Samples were shipped to and sequencing and differential gene expression analysis were performed by Novogene GmbH. GSEA was performed with the GSEA desktop application (Broad Institute), version 4.4.0. Pre-ranked GSEA was conducted with log_2_ fold change as a ranking metric, and 10,000 permutations were performed.

Further details regarding the methods can be found in the supplemental information.

## Resource availability

### Lead contact

Requests for further information and resources should be directed to and will be fulfilled by the lead contact, Sebastian J. Pomplun (s.j.pomplun@lacdr.leidenuniv.nl).

### Materials availability

Unique reagents generated in this study are available from the lead contact with a completed materials transfer agreement. The distribution is limited by stock availability.

### Data and code availability

• All data reported in this paper will be shared by the lead contact upon request.
• This paper does not report original code.
• Any additional information required for reanalyzing the data reported in this paper is available from the lead contact upon request.

## Acknowledgments

J.P.K., B.D.E., and S.J.P. acknowledge funding from the European Research Council Starting Grant (SynTra 101039354). The Pomplun lab gratefully acknowledges financial support from Mr. H.J.M. Roels through a donation to the Oncode Institute and the Dutch Cancer Society’s (KWF) financial support of the Oncode Institute. We also acknowledge the Leiden Institute of Chemistry’s Protein Facility (especially Anneloes Cramer-Blok, Monika Timmer, and Patrick Voskamp) for support and the group of Prof. Dr. Eilers at the University of Wuerzburg for donating the plasmid used for the recombinant expression of Omomyc. The authors thank Sylvia le Dévédec and Kostas Tassis of the Leiden Cell Observatory for their support and assistance with confocal microscopy.

## Author contributions

Conceptualization, S.J.P. and J.P.K.; methodology, J.P.K., B.D.E., V.E.v.d.N., and B.v.d.W.; experiments, J.P.K., B.D.E., V.E.v.d.N., and S.J.P.; data analysis, J.P.K., B.D.E., V.E.v.d.N., B.v.d.W., and S.J.P.; manuscript, J.P.K. and S.J.P., with input from all authors.

## Declaration of interests

J.P.K. and S.J.P. filed a patent application regarding the compounds described in this article.

## Supplemental information

Document S1. Supplemental methods and Figures S1–S22

Document S2. Article plus supplemental information

## Footnotes

Supplemental information can be found online at https://doi.org/10.1016/j.chempr.2025.102839.
