# Supplementary material for: ReCHEMbinant stapling enhances intracellular delivery and bioactivity of engineered protein inhibitors
Source: Chem. 2026 Apr 9;12(4):None. doi: 10.1016/j.chempr.2025.102839 (PMC13065503; doi:10.1016/j.chempr.2025.102839)
Supplement: Document S1. Supplemental methods and Figures S1–S22 [file mmc1.pdf]

Chem, Volume *12*

Supplemental information

ReCHEMbinant stapling enhances intracellular delivery and bioactivity of engineered protein inhibitors

Jan Pascal Kahler, Brecht D. Ellenbroek, Vera E. van der Noord, Bob van de Water, and Sebastian J. Pomplun

Boosting Cell Entry and Bioactivity of Synthetic Transcription Factors via reCHEMbinant Protein Engineering

Jan Pascal Kahler, Brecht D. Ellenbroek, Vera E. van der Noord, Bob van de Water, Sebastian J. Pomplun*

Corresponding Author

*Sebastian J. Pomplun; Leiden University, 2333 CC Leiden, The Netherlands; Oncode Institute, 3521 AL Utrecht, The Netherlands; https://orcid.org/0000-0003-0905-8551; s.j.pomplun@lacdr.leidenuniv.nl

Authors

Jan Pascal Kahler; Leiden University, 2333 CC Leiden, The Netherlands; Oncode Institute, 3521 AL Utrecht, The Netherlands; https://orcid.org/0000-0001-9997-9404

Brecht D. Ellenbroek; Leiden University, 2333 CC Leiden, The Netherlands; Oncode Institute, 3521 AL Utrecht, The Netherlands; https://orcid.org/0000-0001-9987-9825

Vera E. van der Noord; Leiden University, 2333 CC Leiden, The Netherlands; https://orcid.org/0000-0002-9894-3239

Bob van de Water; Leiden University, 2333 CC Leiden, The Netherlands; https://orcid.org/0000-0002-5839-2380

Table of contents

List of abbreviations 4

Supplementary Figures 5

Figure S1. Predicted structure of ArtiMYC. 5

Figure S2. Independent reporter gene assay triplicates. 6

Figure S3. Control experiments to validate reporter gene assay data 7

Figure S4. Synthesis and characterization of NucleoMYC 8

Images of uncut EMSA gels 9

Figure S5. Uncut EMSA gel image of Omomyc 1 9

Figure S6. Uncut EMSA gel image of ArgiMyc 2 10

Figure S7. Uncut EMSA gel image of ArtiMyc 3 11

Figure S8. Uncut EMSA gel image of HeloMyc-1421 8 12

Figure S9. Uncut EMSA gel image of HeloMyc-714 9 13

Figure S10. Uncut EMSA gel image of HeloMyc-37 10 14

Figure S11. Uncut EMSA gel image of HeloMyc-711 11 15

Figure S12. Uncut EMSA gel image of NuceloMYC 16 16

Figure S13. Uncut EMSA gel image of NuceloMYC 17 16

Supplemental Methods 17

General 17

LC-MS 17

Synthesis 17

Stapling reactions 17

Stapling with i, i+7 staple (4,4′-Bis(bromomethyl)biphenyl) 17

Figure S14. LCMS traces of i, i+7 stapled proteins 18

Stapling with i, i+4 staple (α,α′-Dibromo-m-xylene) 19

Figure S15. LCMS traces of i, i+4 stapled proteins 19

Cys-capping of 4 with benzyl bromide 19

Figure S16. LCMS trace of BenzoMYC 20

FITC labelling 20

Labelling Omomyc 20

Figure S17. LC-MS spectrum with total ion count, extracted ion count and deconvoluted mass of FITC-labelled OmoMyc (12). 20

Labelling HeloMYC-1421 20

Figure S18. LC-MS spectrum with total ion count, extracted ion count and deconvoluted mass of FITC-labelled HeloMyc-1421 (13). 21

Serum stability 21

Figure S19. HeloMYC-1421 displays improved serum stability compared to Omomyc 21
Synthesis of probe 18 22
Figure S20. Synthesis scheme and LCMS trace and spectrum of probe 18. 22
Synthesis of probe 19 23
Figure S21. Synthesis scheme and LCMS trace and spectrum of probe 19 23
Synthesis of protein conjugates 20 and 21 24
Protein expression and characterization 24
Site-directed mutagenesis 24
Primers used for site-directed mutagenesis 25
Primers used for sequencing 25
Expression of different Omomyc variants 25
Purification of His-tagged proteins 25
General protocol for buffer exchange 26
Tag cleavage with enterokinase and subsequent purification 26
Electromobility shift assay (EMSA) 26
Circular Dichroism Spectroscopy 26
Cell culture and cell assays 27
MYC reporter gene assay 27
MYC reporter assay control for direct luciferase inhibition 27
Cell proliferation assay 27
Protein sequences 28
Plasmid sequences 29
RNA-seq genes up genes down 38
Figure S22. Volcano plot of differentially expressed genes. 38
Overview of all produced miniproteins 50
References 53

List of abbreviations

MeCN	Acetonitrile
SPPS	Solid-phase peptide synthesis
AUC	Area under the curve
CV	Column volume
DPBS	Dulbecco's phosphate buffered saline
EDTA	Ethylenediaminetetraacetic acid
EK	Enterokinase
EMSA	Electrophoretic mobility shift assay
FITC	Fluorescein isothiocyanate
HPLC	High-performance liquid chromatography
IS	Internal standard
LC-MS	Liquid chromatography-mass spectrometry
NEAA	Non-essential amino acid
PBS	Phosphate buffered saline
PDB	Protein data bank
RT	Room temperature
RP-FC	Reverse phase flash chromatography
SDS-PAGE	Sodium dodecyl sulphate polyacrylamide gel electrophoresis
SPPS	Solid-phase peptide synthesis
TBE	Tris borate EDTA buffer
TCEP	Tris(2-carboxyethyl)phosphine
TFA	Trifluoroacetic acid
TIC	Total-ion chromatogram
XIC	Extracted-ion chromatogram

Supplementary Figures

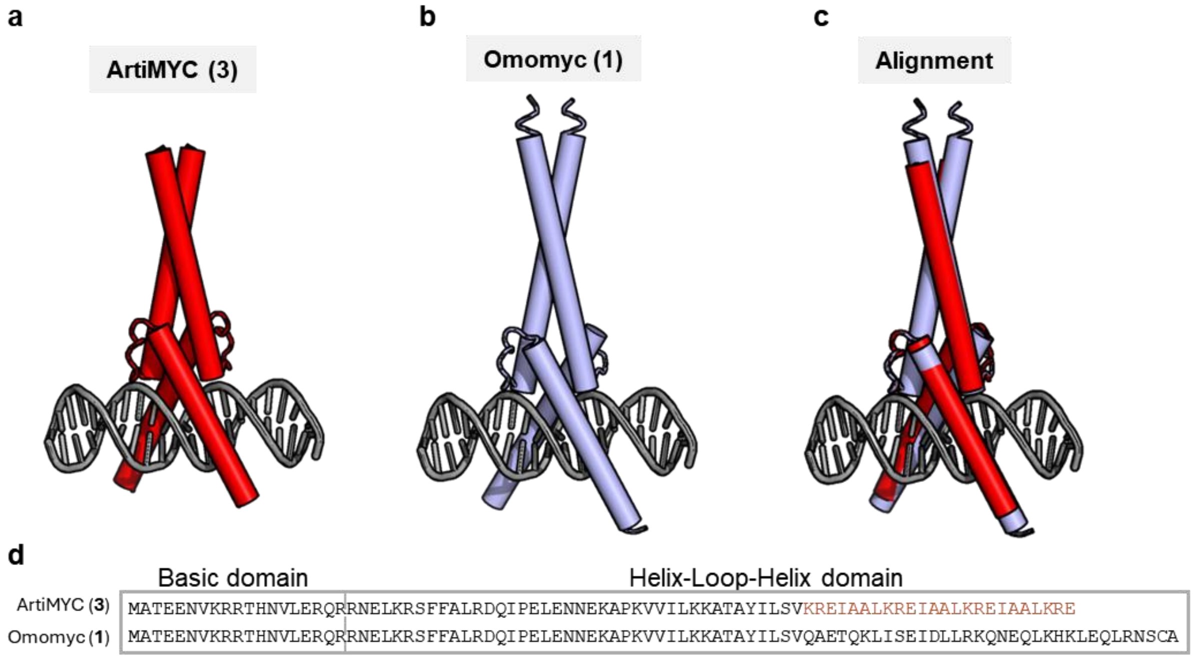

Figure S1. Predicted structure of ArtiMYC. Folding of Omomyc (**1**) and ArtiMYC (**3**) is very similar. (**a-c**) Structures of ArtiMYC (**3**, a) and Omomyc (**1**, b) aligned (c). (**d**) The sequences of ArtiMYC (**3**) and Omomyc (**1**) with the artificial coiled-coil highlighted. Structure of ArtiMYC (**3**) was predicted using AlphaFold[1] and aligned to Omomyc (**1**) using Pymol. DNA shown in all panels is DNA from alignment with Omomyc (**1**).

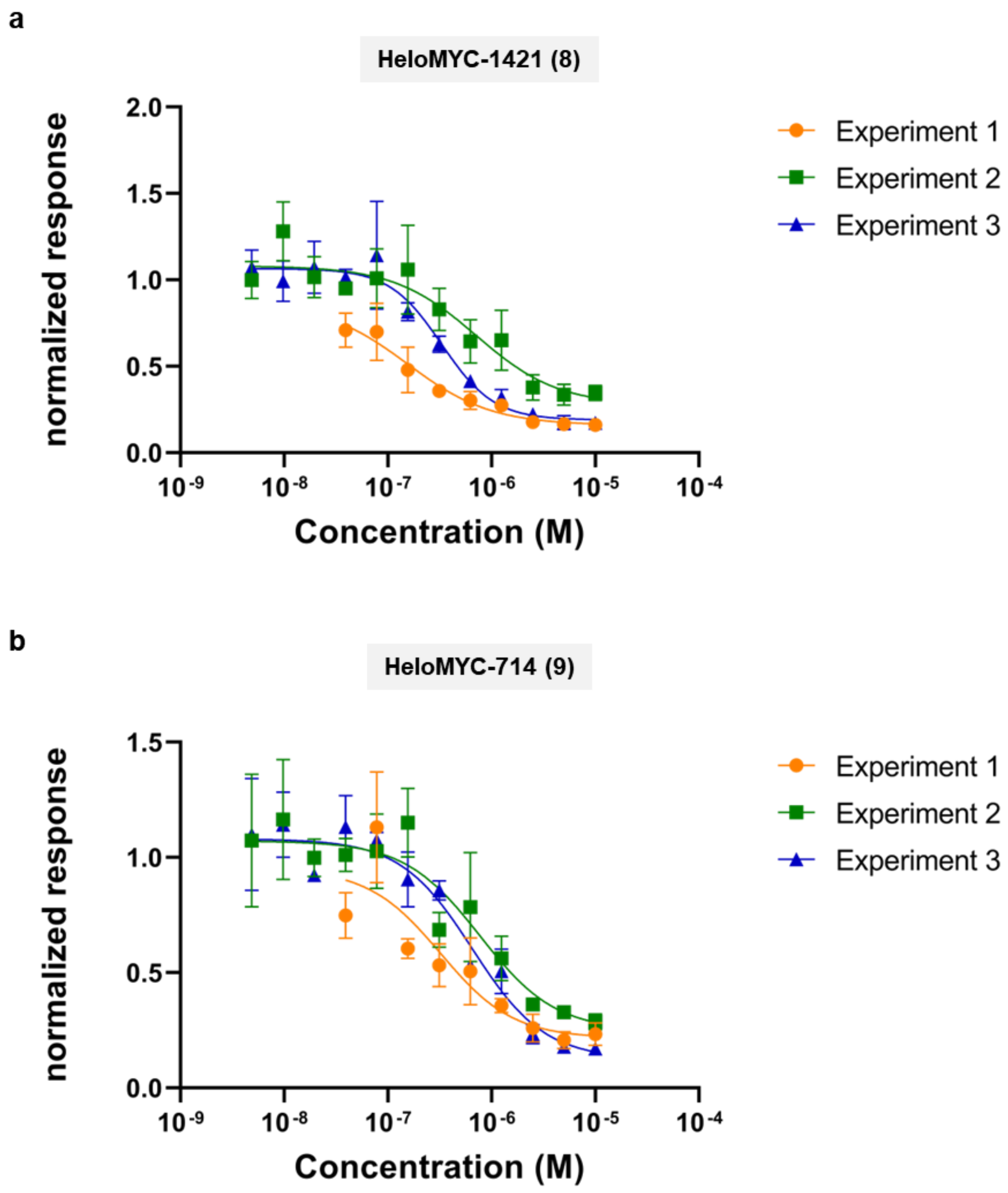

Figure S2. Independent reporter gene assay triplicates. Results of three independent MYC reporter gene assay experiments to determine the EC_{50} of HeloMYC-1421 (**8**, **a**) and HeloMYC-714 (**9**, **b**). Results shown have no outliers removed and might differ slightly from the one curve shown in the main paper figure 4b.

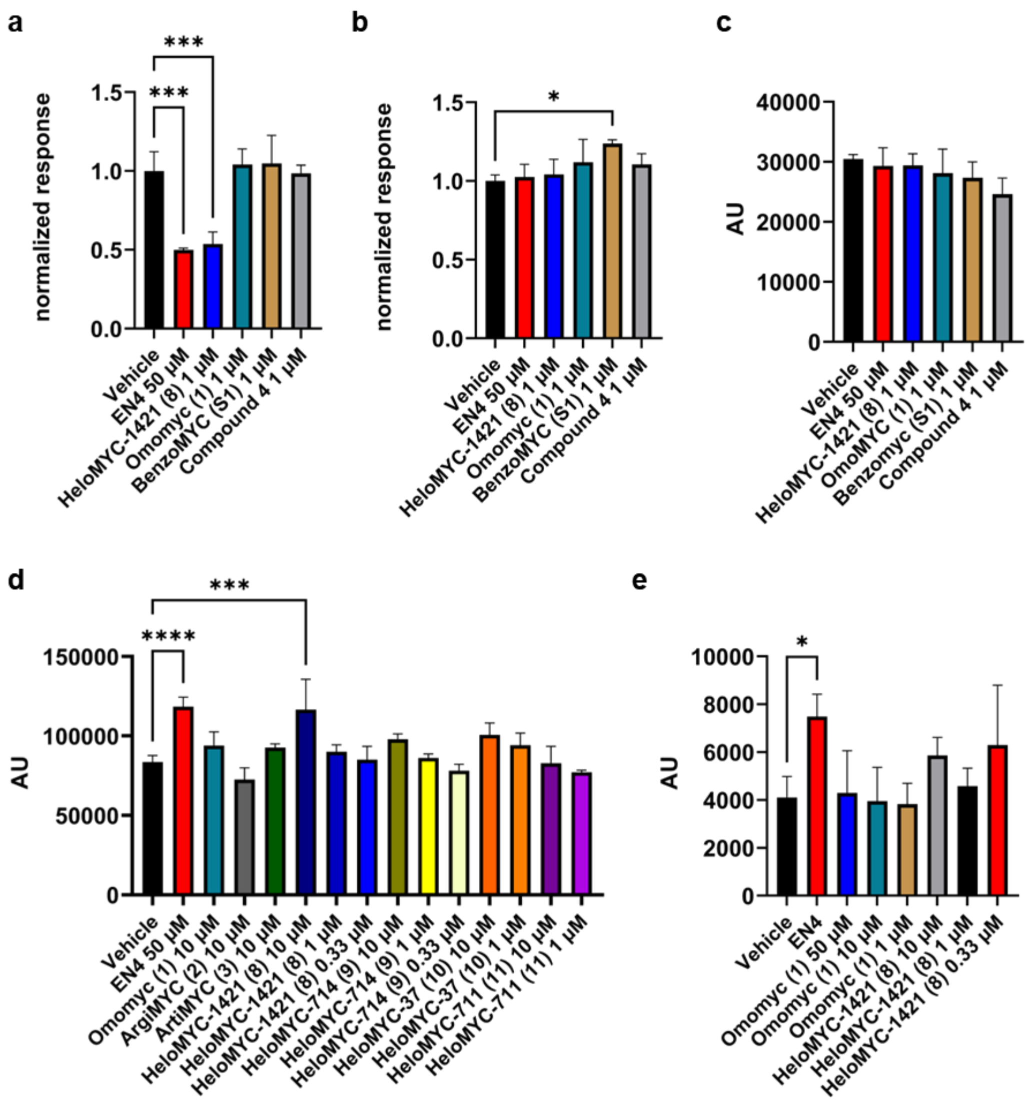

Figure S3. Control experiments to validate reporter gene assay data. **(a)** Positive control experiment shows effect of HeloMYC-1421 and EN4 on luciferase expression. HEK293T cells were transfected with a plasmid in which not only renilla luciferase expression but also firefly luciferase expression is under control of the CMV promoter instead of a MYC-responsive element. **(b)** Miniproteins are no direct luciferase inhibitors. HEK293T cells were transfected with the same plasmid as in panel a but treated with the indicated miniproteins shortly before cell lysis and addition of luciferase substrate to rule out any effect on gene transcription and expression. (**c-e**) Miniproteins have negligible effect on renilla luciferase expression under control of the CMV promoter. Shown is the renilla luciferase signal used for signal normalization of the experiments shown in figure S3a (panel c) and figure 4a and b (panels d and e, respectively) in the main text. **Statistics.** A one way ANOVA was performed to compare the effect of miniprotein treatment on firefly or renilla luciferase expression showing that there was a (a, b, d, e) or no (c) statistical difference between treatments (a) F (5, 12) = 18.73, P<0.0001; (b) F (5, 12) = 3.115, P=0.0496; (c) F (5, 12) = 1.780, P=0.1915; (d) F (14, 30) = 8.662, P<0.0001; (e) F (7, 16) = 2.889, P=0.0373.

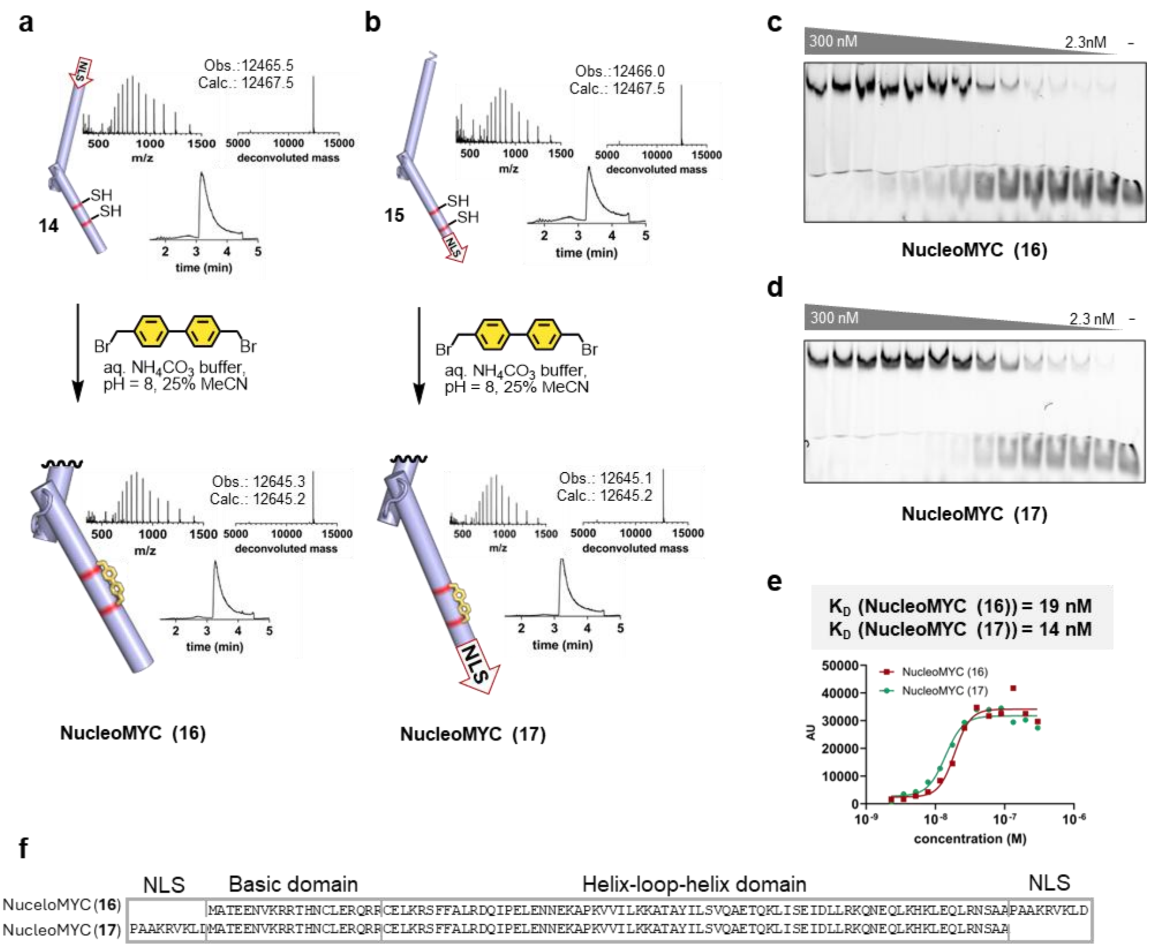

Figure S4. Synthesis and characterization of NucleoMYC. NucleoMYC containing a nuclear localization sequence (NLS) fused to HeloMYC-1421 binds to E-Box DNA. (**a, b**) An NLS sequence can be fused C- or N-terminally to the HeloMYC-1421 sequence, expressed and subsequently stapled, yielding NucleoMYC proteins **16** and **17**. (**c, d, e**) Both NucleoMYC proteins bind to E-BOX DNA in an EMSA with a K_D of 19 and 14 nM, respectively. (**f**) The sequence of NLS-containing NucleoMYC proteins.

Images of uncut EMSA gels

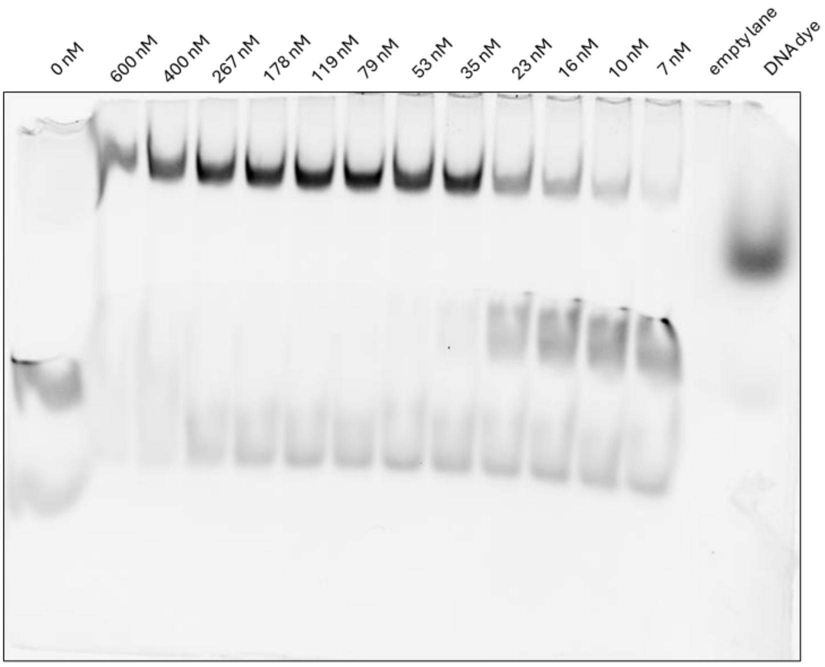

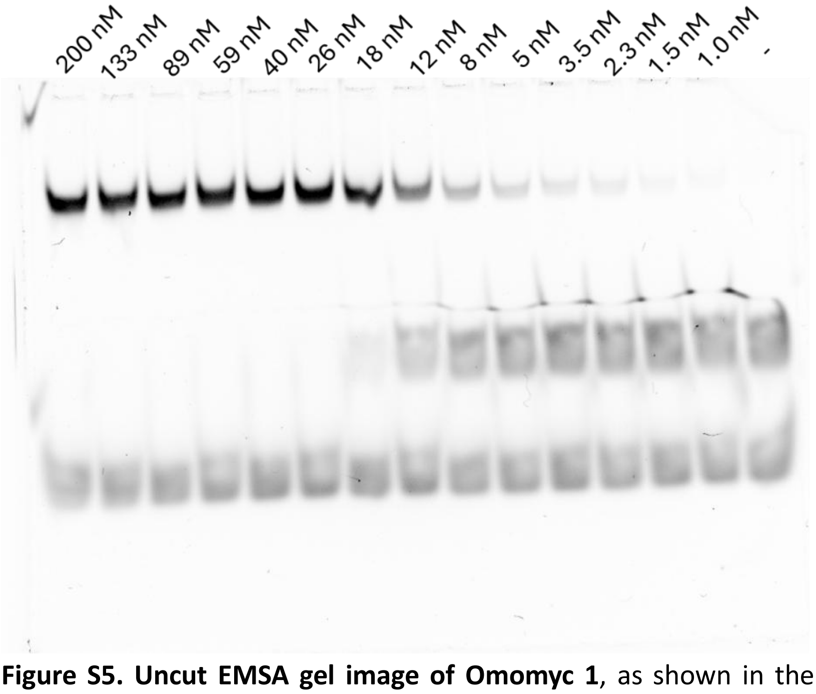

Figure S5. Uncut EMSA gel image of Omomyc 1, as shown in the main text in Figure 3a (up) and independent replicate (down).

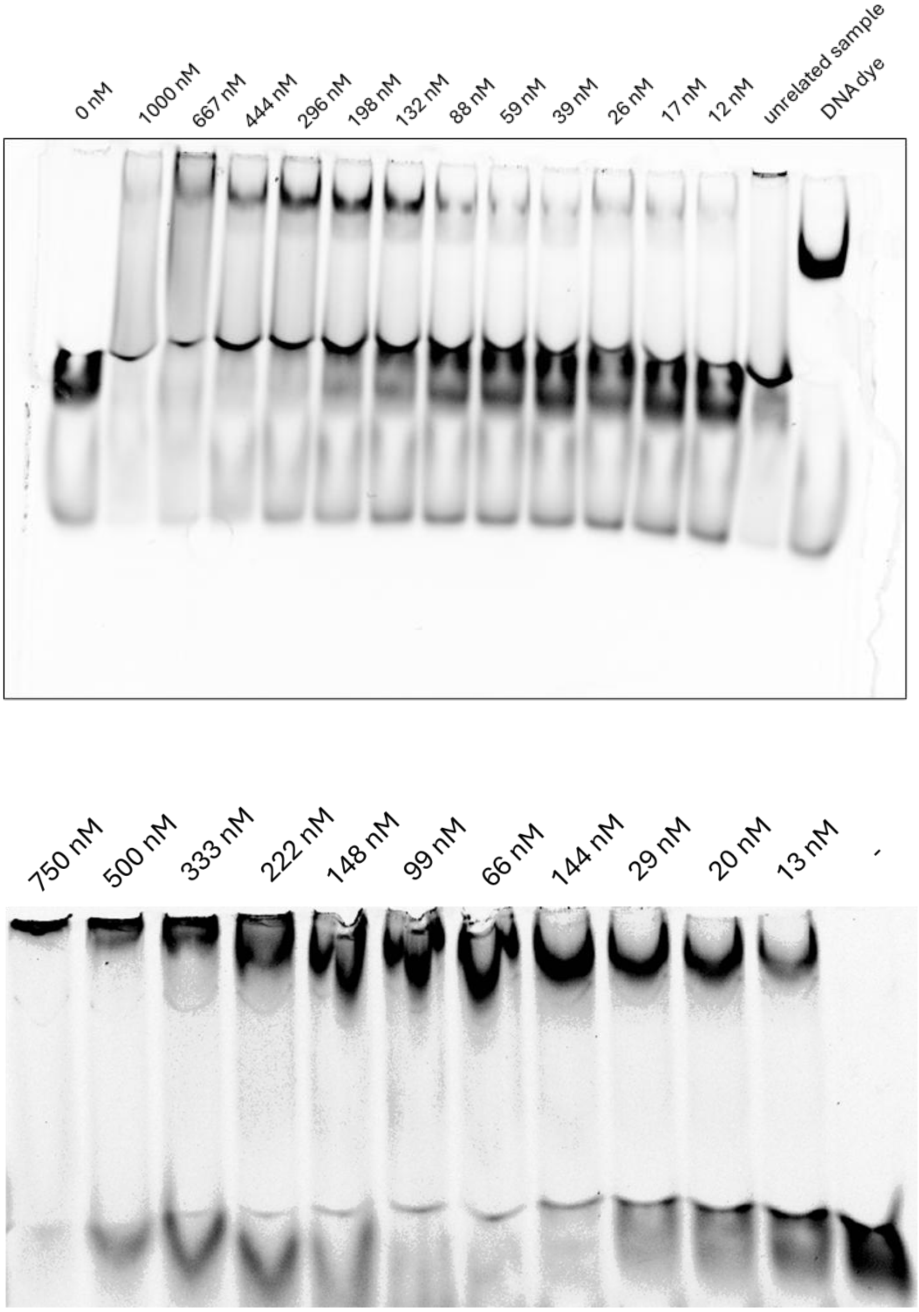

Figure S6. Uncut EMSA gel image of ArgiMyc 2, as shown in the main text in Figure 3a (up) and independent replicate (down).

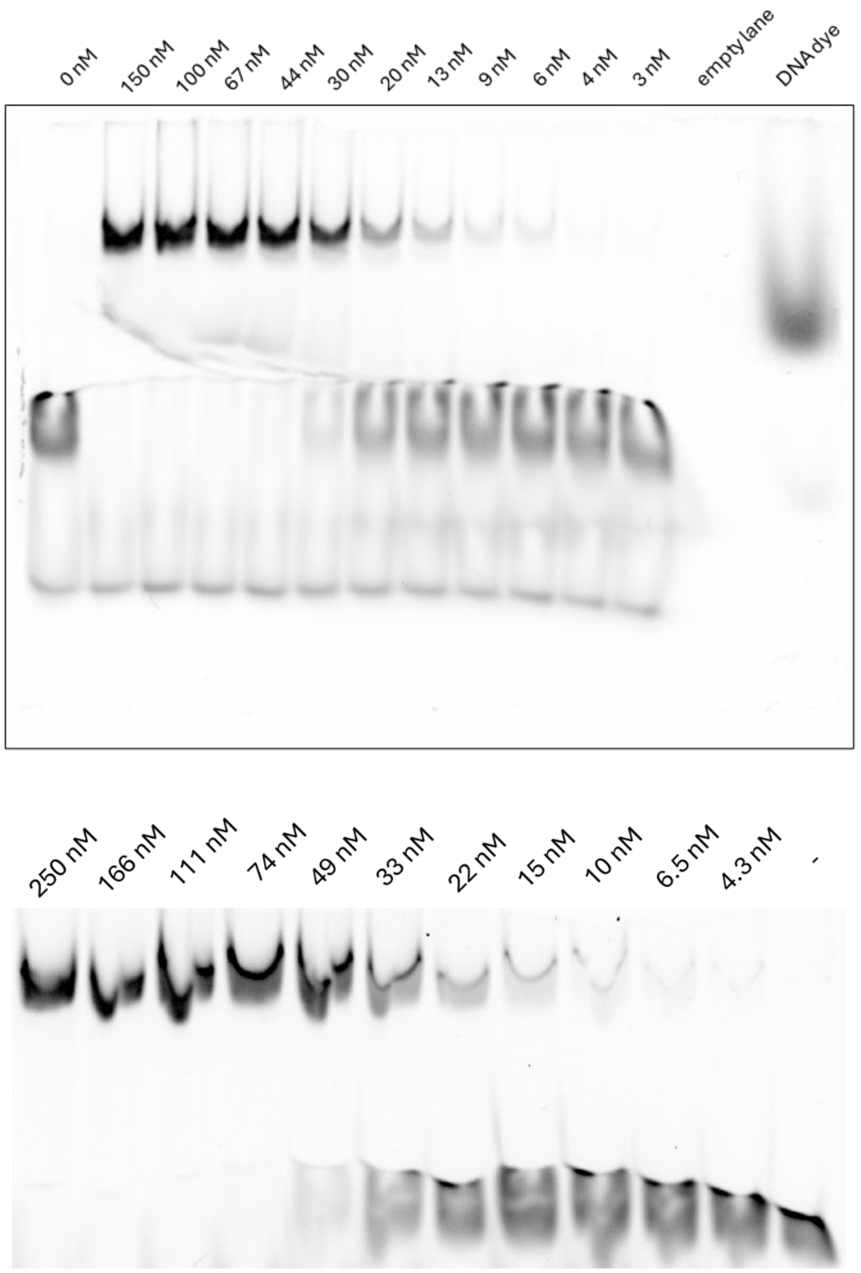

Figure S7. Uncut EMSA gel image of ArtiMyc 3, as shown in the main text in Figure 3a (up) and independent replicate (down).

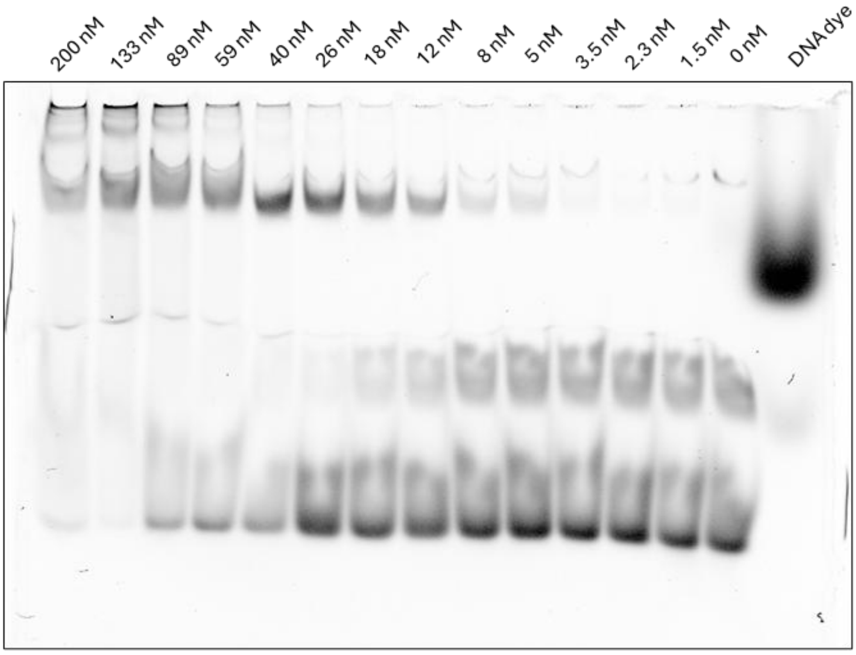

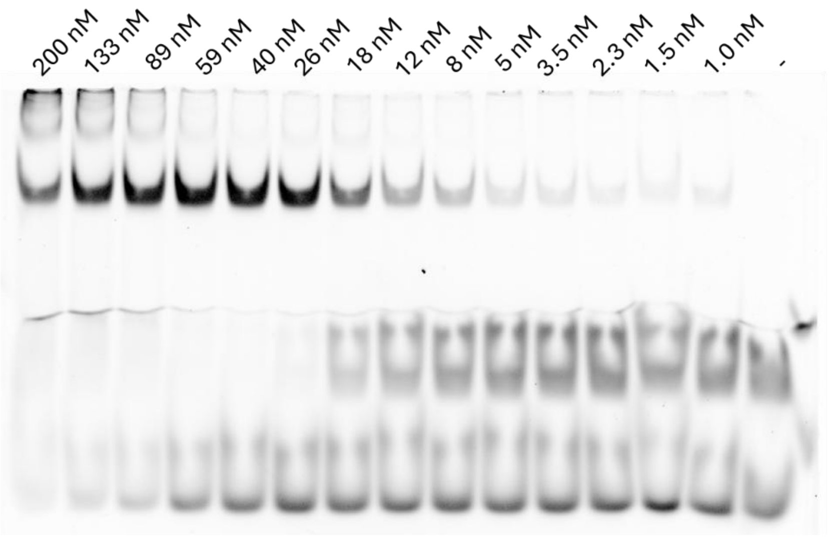

Figure S8. Uncut EMSA gel image of HeloMyc-1421 8, as shown in the main text in Figure 3a (up) and independent replicate (down).

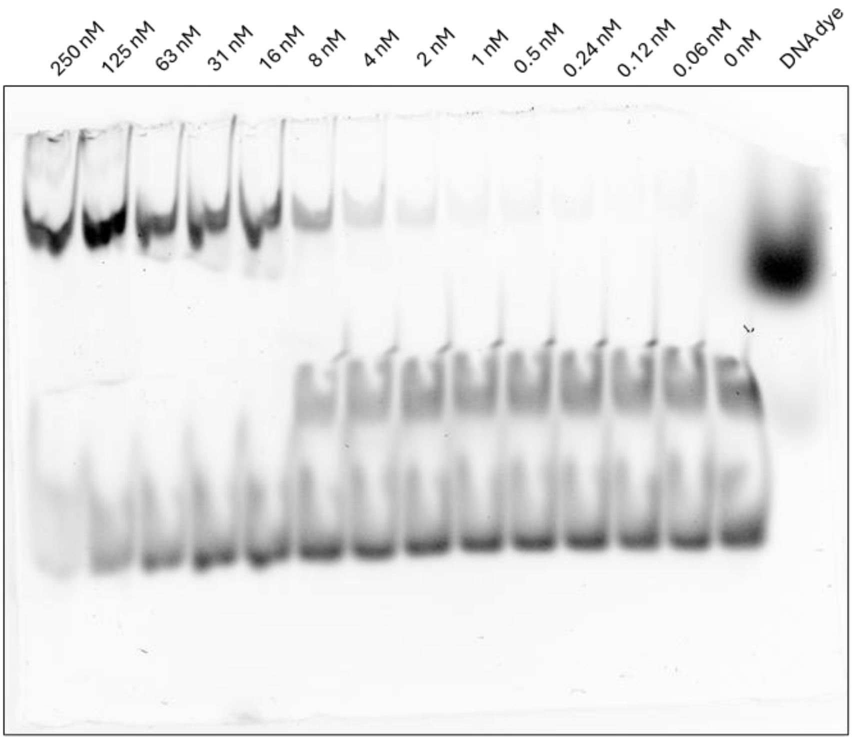

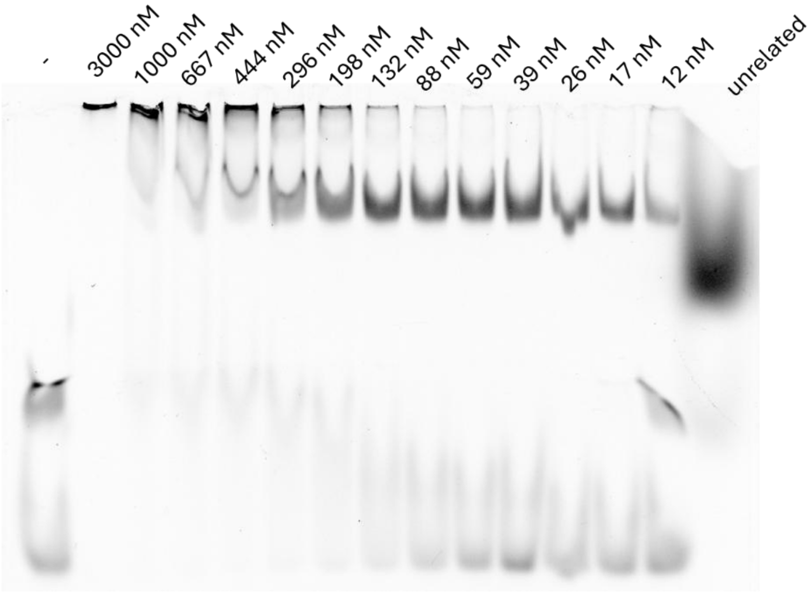

Figure S9. Uncut EMSA gel image of HeloMyc-714 9, as shown in the main text in Figure 3a (up) and independent replicate (down).

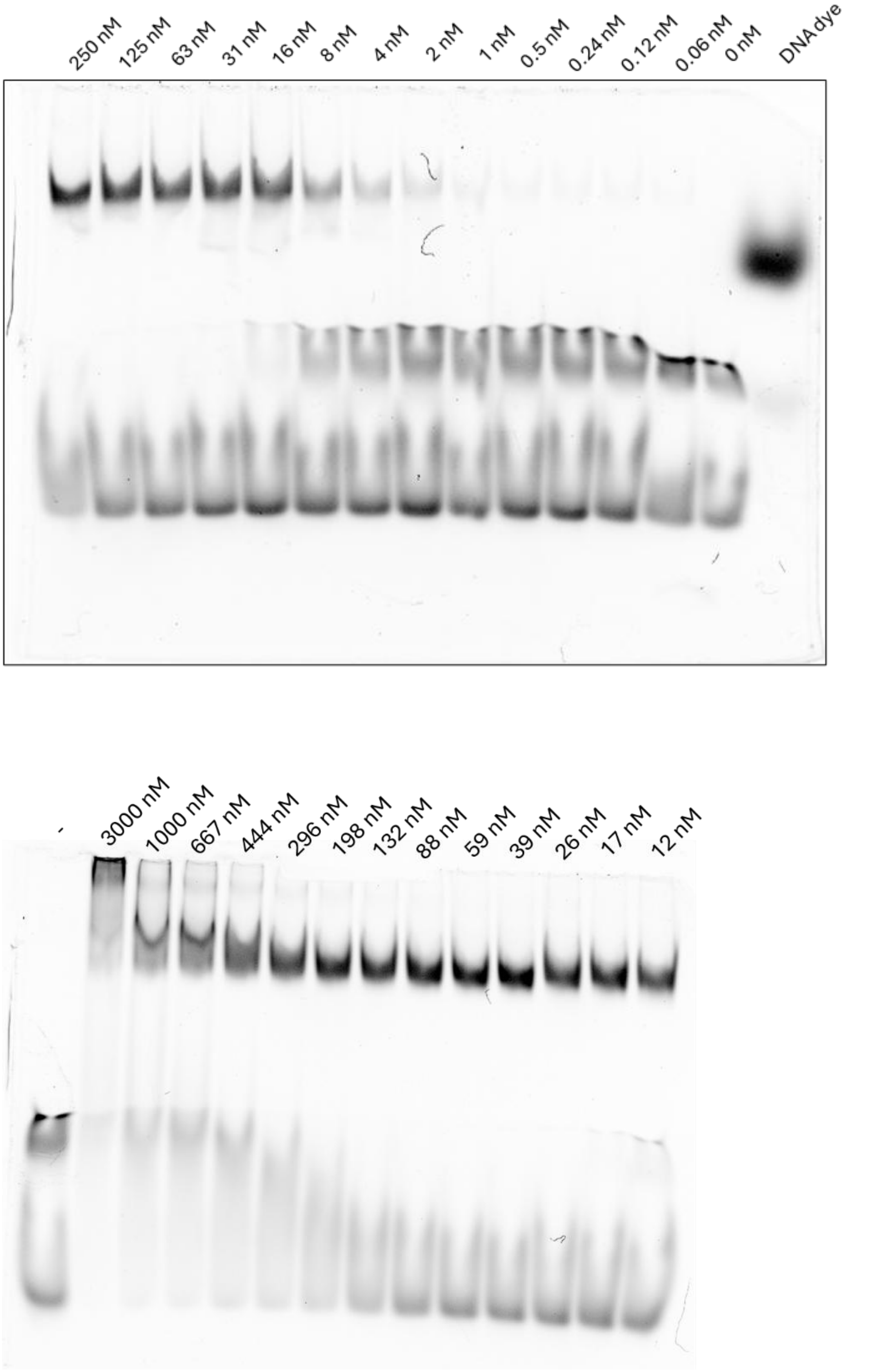

Figure S10. Uncut EMSA gel image of HeloMyc-37 10, as shown in the main text in Figure 3a (up) and independent replicate (down).

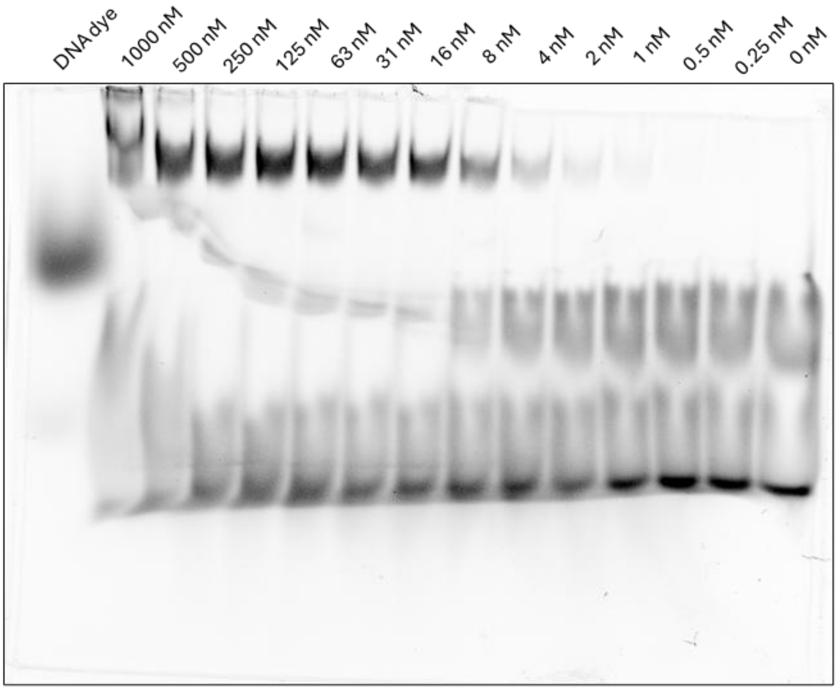

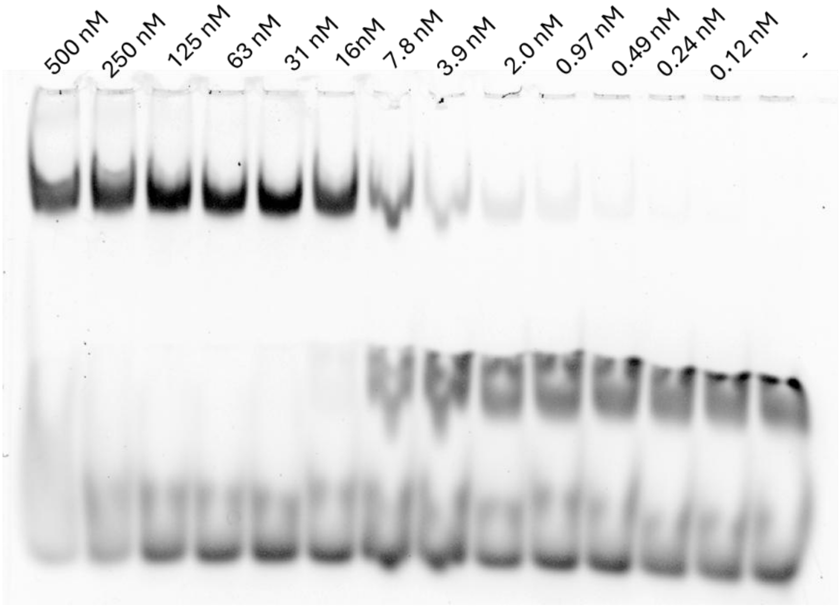

Figure S11. Uncut EMSA gel image of HeloMyc-711 11, as shown in the main text in Figure 3a (up) and independent replicate (down).

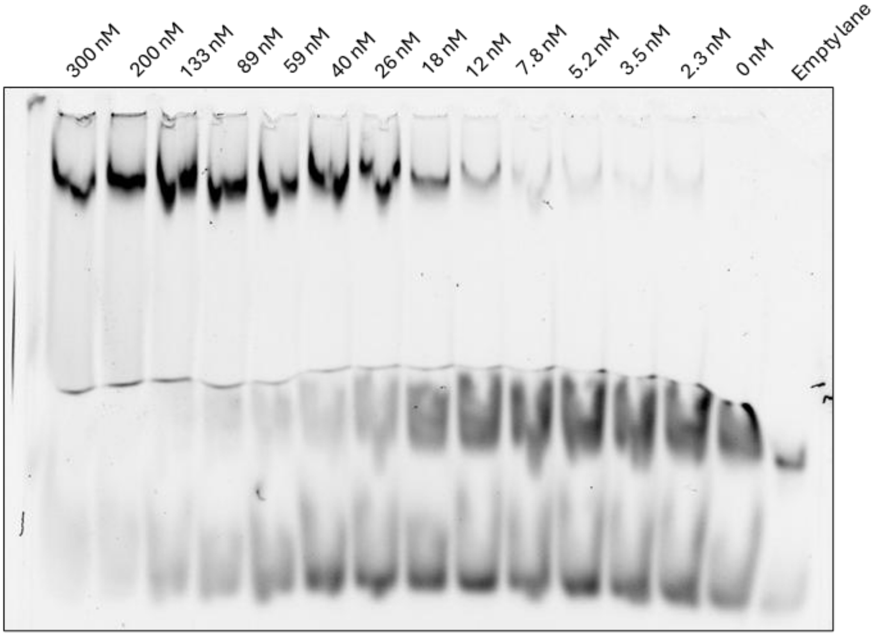

Figure S12. Uncut EMSA gel image of NuceloMYC 16, as shown in the SI in Figure S4c.

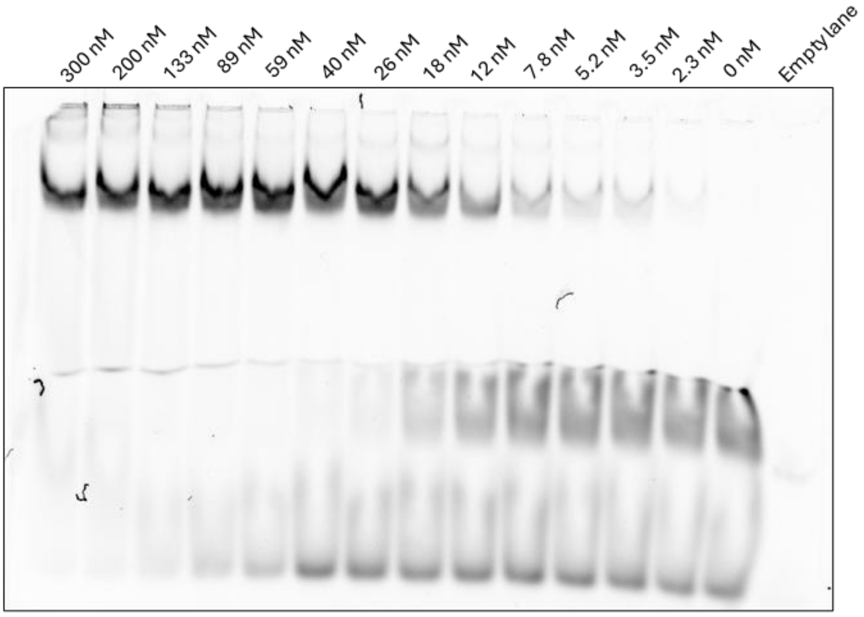

Figure S13. Uncut EMSA gel image of NuceloMYC 17, as shown in the SI in Figure S4d.

Supplemental Methods

General

All reactions were carried out using commercially available reagents, unless noted otherwise. Reagents and solvents were purchased from Sigma-Aldrich (Merck), Fisher Scientific, or VWR chemicals.

Plasmid pET-30a with 6-his-tagged Omomyc was donated by the group of prof. dr. Eilers (University of Würzburg, Germany) and sequenced before being used. Primers for site-directed DNA mutagenesis were purchased from IDT (Leuven, Belgium). Plasmids with ArgiMYC, ArtiMYC and NucleoMYCs were obtained from genscript.

LC-MS

LC-MS chromatograms and associated mass spectra were acquired using a Shimazu LCMS-2020 system (Method A) or, for high resolution mass spectrometry data, a Sciex X500b QTOF ESI-QToF mass spectrometer coupled to a Shimadzu Nexera UHPLC LC40DX3 (Method B). Mobile phases used for LC-MS analysis are solvent A (0.1% formic acid in water) and solvent B (0.1% formic acid in acetonitrile).

The following LCMS methods were used:

Method A

Column: Kinetex® 2.6µm XB-C18 100 Å LC Column (50 x 3 mm) UV detector: 214 nm.

LC Method: 0% solvent B over 1 min, followed by a linear gradient 0% to 70% solvent B over 10 min, followed by 70% solvent B over 1.5 min, followed by 70% to 0% solvent B over 4.5, flowrate 0.55 mL/min

Method B (high resolution)

Column: Phenomenex Synergi™ 4 µm Fusion-RP 80 Å LC Column (50 x 2 mm)

LC Method: 0% solvent B over 1 min, followed by a linear gradient 0% to 60% solvent B over 3.5 min, followed by a linear gradient 60% to 95% solvent B over 0.1 min, followed by 95% solvent B over 0.4 min, followed by a linear gradient 95% to 0% solvent B over 0.5 min, followed by 0% solvent B over 1.5 min, flowrate 0.5 mL/min.

MS parameters: General parameters: Method duration: 5 min; Total scan time: 0.276 sec; Estimated cycles: 1086; Intact protein mode: False; Decrease detector voltage: False; Large protein (>70 kDa): False; Ion Source: Source name: TurboIonSpray; Curtain gas: 35 psi; Ion source gas 1: 60 psi; Ion source gas 2: 60 psi; Temperature: 500 ℃; Experiment: Scan type: TOF MS; Polarity: Positive; Spray voltage: 5500 V; CAD gas: 7; Time bins to sum: 4; Channel 1-4: True; TOF start mass: 350 Da; TOF stop mass 1500 Da; Accumulation time: 0.25; Declustering potential: 80V; Declustering potential spread: 0 V; Collision energy: 10V; Collision energy spread: 0 V; Override Qjet RF value: False

Method C (high resolution)

Column: Phenomenex Synergi™ 4 µm Fusion-RP 80 Å LC Column (50 x 2 mm) or Aeris™ 3.6 µm Widepore XB-C18.

LC Method: 0% solvent B over 1 min, followed by a linear gradient 0% to 90% solvent B over 6 min, followed by a 90% solvent B over 2 min, followed by a linear gradient of 90% to 0% solvent B over 0.5 min, followed by 0% solvent B over 1.5 min, flowrate 0.5 mL/min.

Synthesis

Stapling reactions

Stapling with i, i+7 staple (4,4'-Bis(bromomethyl)biphenyl)

The respective protein (1 eq., **4** or **5**) was dissolved in water (2 mM, e.g. 30 mg of **5** in 1.08mL) and then diluted to 100 µM into stapling buffer (15.12 mL for **5**, NH_4HCO_3, 100 mM, pH= 8). 4,4'-Bis(bromomethyl)biphenyl was dissolved at 4x the final concentration in MeCN (500 µM) and then

added (1.25 eq., 5.4 mL for HeloMYC-1421) to the protein in stapling buffer. Final concentrations used were 100 µM (1 eq.) protein and 125 µM (1.25 eq.) staple. Final MeCN content was 25%.
When the stapling reaction was completed as seen by LC-MS the product was purified using a Biotage® Selekt Flash Purification System equipped with a Biotage® Sfär C18 D - Duo 10 g column on a gradient of water in MeCN (0-100%,with a flat gradient between 15% and 79%), both containing 0.1% TFA. The product containing fractions were lyophilized yielding the proteins as TFA salts.

Yield HeloMYC-1421 (**8**): 20.7 mg (69%)
Yield HeloMYC-714 (**9**): 1.94 mg (67%)
Yield NucleoMYC (**16)**: 3.02 mg (60%)
Yield NucleoMYC (**17**): 3.71 mg (46%)

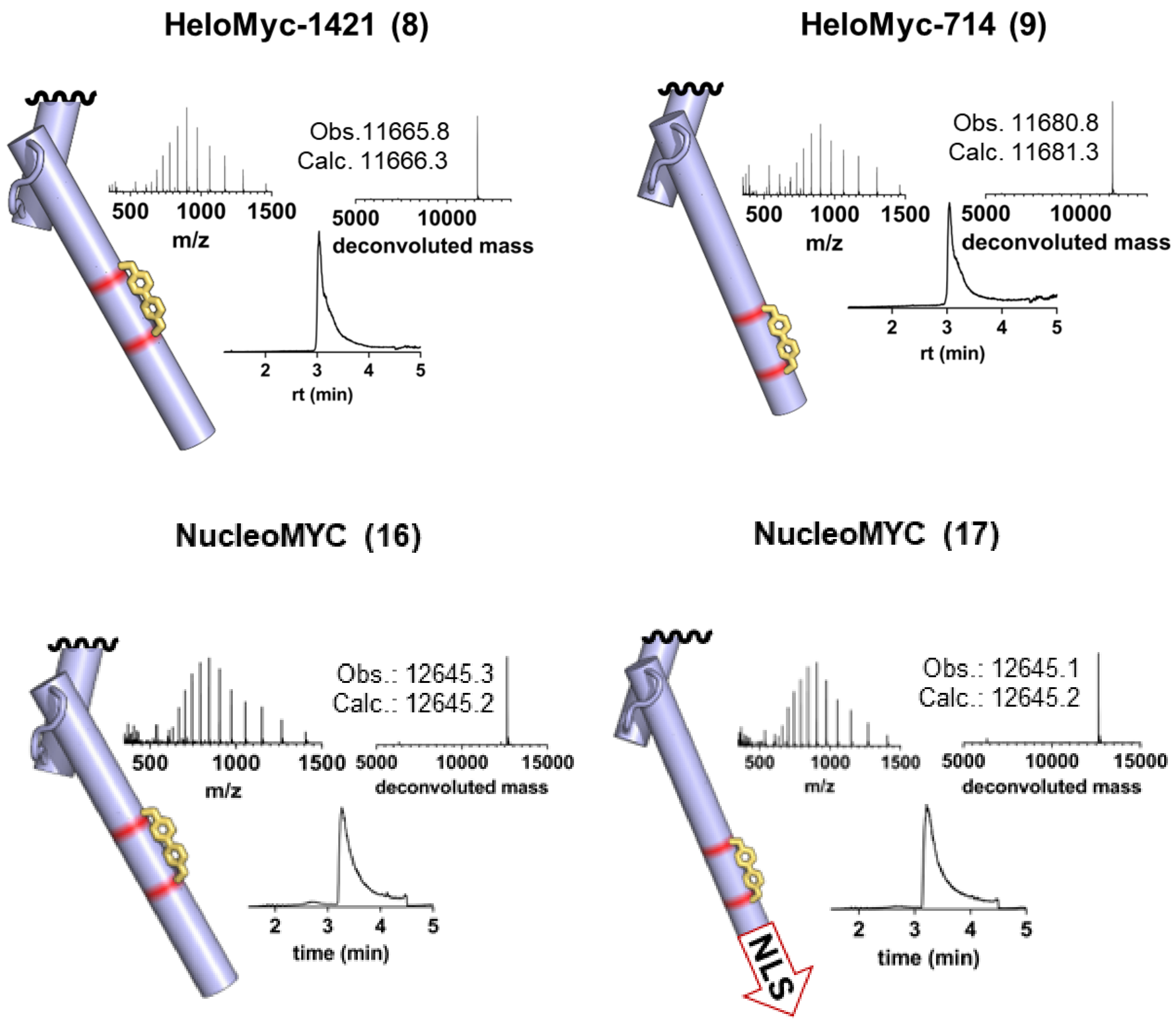

Figure S14. LCMS traces of i, i+7 stapled proteins

Stapling with i, i+4 staple (α,α′-Dibromo-m-xylene)
The respective protein (1 eq., **6** or **7**) was dissolved in water (e.g. 1.96 mg of **7** in 70.53 µL) and then diluted into stapling buffer (973 µL for **7**, NH_4HCO_3, 100 mM, pH= 8). TCEP was dissolved at 100x the final concentration in water (20 mM) and added to the stapling reaction (2 eq., 14.1 µL). α,α′-Dibromo-m-xylene was dissolved at 4x the final concentration in MeCN (500 µM) and then added (1.25 eq., 353 µL for HeloMYC-711) to the protein in stapling buffer. Final concentrations used were 100 µM (1 eq.) protein, 125 µM (1.25 eq.) staple and 200 µM (2 eq.) TCEP. Final MeCN content was 25%.
When the stapling reaction was completed as seen by LC-MS the product was purified using a Biotage® Selekt Flash Purification System equipped with a Biotage® Sfär C18 D - Duo 10 g column on a gradient of water in MeCN (0-100%,with a flat gradient between 15% and 79%), both containing 0.1% TFA. The product containing fractions were lyophilized yielding the proteins as TFA salts.

Yield HeloMYC-37 (**10**): 0.27 mg (38%)
Yield HeloMYC-711 (**11**): 0.32 mg (26%)

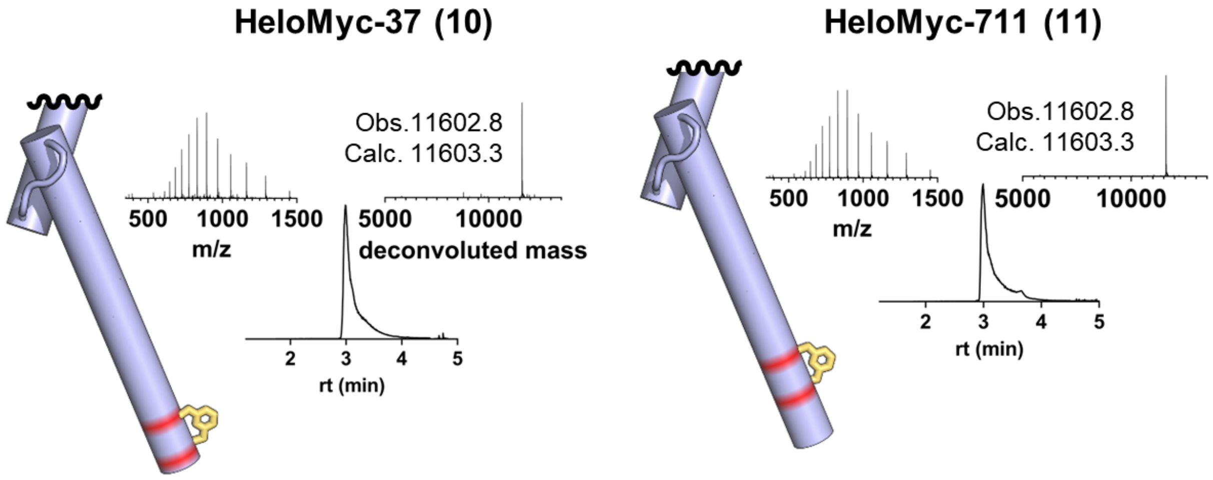

Figure S15**. LCMS traces of i, i+4 stapled proteins**

Cys-capping of 4 with benzyl bromide
Protein **4** (2.8 mg, 202 nmol, 1eq.) was dissolved at 20x the final concentration in water (101 µL, 2 mM) and then diluted into stapling buffer (NH_4HCO_3, 100 mM, pH= 8). Benzyl bromide was dissolved at 4x the final concentration in MeCN (1 mM) and then added to the protein in stapling buffer (505 µL, 2.5 eq.). Final concentrations used were 100 µM (1 eq.) for the protein and 250 µM (2.5 eq.) for benzyl bromide. Final MeCN content was 25%.
When the stapling reaction was completed as seen by LC-MS the product was purified using a Biotage® Selekt Flash Purification System equipped with a Biotage® Sfär C18 D - Duo 10g column on a gradient of water in ACN, both containing 0.1% TFA. The product containing fractions were lyophilized yielding BenzoMYC (**S1**) (1.06 mg, 75 nmol, 37%) as TFA salt. In addition to the main peak which corresponds to the desired product, a second deconvoluted mass corresponding to an additional benzyl substitution is detected in the product.

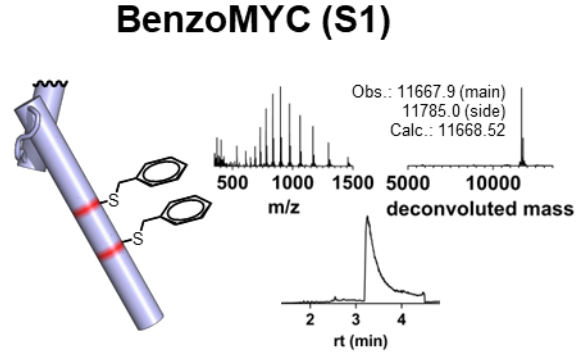

Figure S16**. LCMS trace of BenzoMYC**

FITC labelling

Labelling Omomyc

OmoMyc (4.19 mg, 300 nmol, 1 eq.) was dissolved in carbonate buffer (0.1 M, pH 9.1, 4.19 mL) to a concentration of 1 mg/mL and cooled to 0 °C. Subsequently, a solution of FITC in dmso (1.7 mM, 159 µg, 240 µL, 408 nmol, 1.36 eq.) was added slowly over the course of approximately 4 h. The crude product was then purified by reverse phase column chromatography using a Biotage® Selekt Flash Purification System with a Biotage® Sfär C18 D - Duo 100 Å 30 µm column 10 g applying a gradient of 0-100% MeCN in water (0-100% with a flat gradient between 20% and 48%) yielding FITC-labelled Omomyc-F (**12**) (0.93 mg, 65 nmol, 22%) as TFA salt after lyophilization.

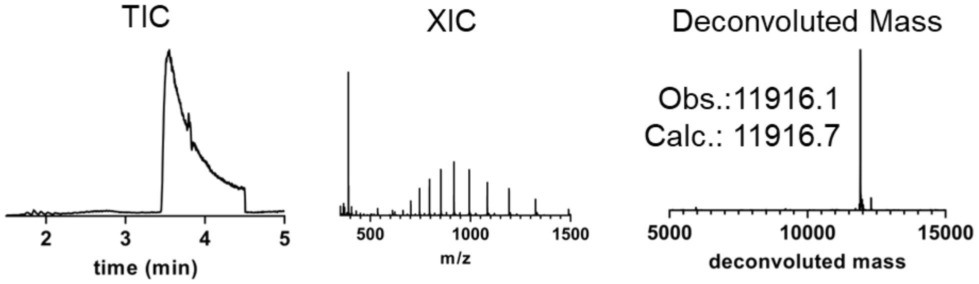

Figure S17**. LC-MS spectrum with total ion count, extracted ion count and deconvoluted mass of FITC-labelled OmoMyc (12).**

Labelling HeloMYC-1421

HeloMYC-1421 (1.35 mg, 96 nmol, 1 eq.) was dissolved in carbonate buffer (0.1 M pH 9.1, 1.35 mL) to a concentration of 1mg/mL and cooled to 0°C. Subsequently, a solution of FITC in dmso (1.56 mM, 67.7 µL, 106 nmol, 1.1 eq.) was added over the course of 4.5 h. The crude product was then purified by reverse phase column chromatography using a Biotage® Selekt Flash Purification System with a Biotage® Sfär C18 D - Duo 100 Å 30 µm column 10 g applying a gradient of 0-100% MeCN in water (0-100% with a flat gradient between 20% and 48%), yielding FITC-labelled HeloMYC-1421-F (**13)** (0.59 mg, 41 nmol, 43%) after lyophilization.

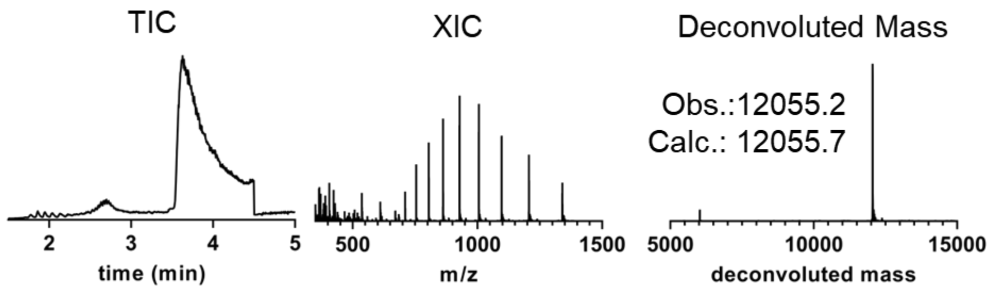

Figure S18**. LC-MS spectrum with total ion count, extracted ion count and deconvoluted mass of FITC-labelled HeloMyc-1421 (13).**

Serum stability

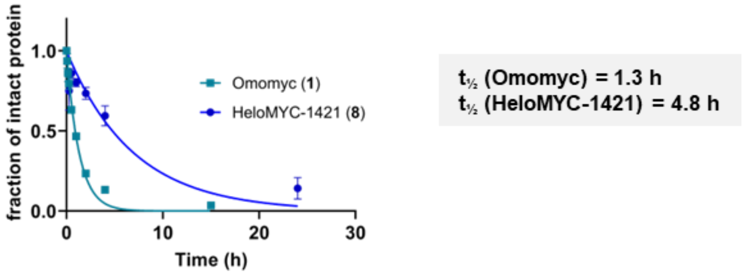

Figure S19. HeloMYC-1421 displays improved serum stability compared to Omomyc.

For serum stability assays 1 mM protein stocks were diluted to a final concentration of 60 µM in 10% human serum in DPBS. The mixture was vortexed immediately after protein addition and 5 µL aliquots were mixed with 5 µL of 20% TFA in water (t = 0) to quench the human serum, resulting in protein precipitating. Subsequently, the protein serum solution was incubated at 37 °C using a BIO-RAD T100™ Thermal Cycler with the lid set to 95 °C. At indicated timepoints 5 µL aliquots were mixed with 5 µL of 20% TFA in water. The resulting pellet was then diluted 5.5x with additional DPBS to redissolve. This solution was analyzed according to the high resolution LCMS protocol (Method C). As internal standard (IS), the extracted-ion chromatogram (XIC) from m/z = 1233.52 was used belonging to human serum albumin. After measurement, the XIC obtained from the highest intensity peak belonging to each protein and IS were extracted from the total-ion chromatogram (TIC). Using Graphpad Prism 9, the area under the curve (AUC) from each timepoint was calculated, normalized against the AUC of the IS followed by normalization against t = 0 and plotted. Next, a nonlinear regression – One phase decay analysis was performed to obtain $t_{½}$ with a plateau constant equal to 0 and Y_0 set to 1. The assay was performed in triplicates. For Omomyc one set of outliers in the measurement was excluded.

Synthesis of probe 18

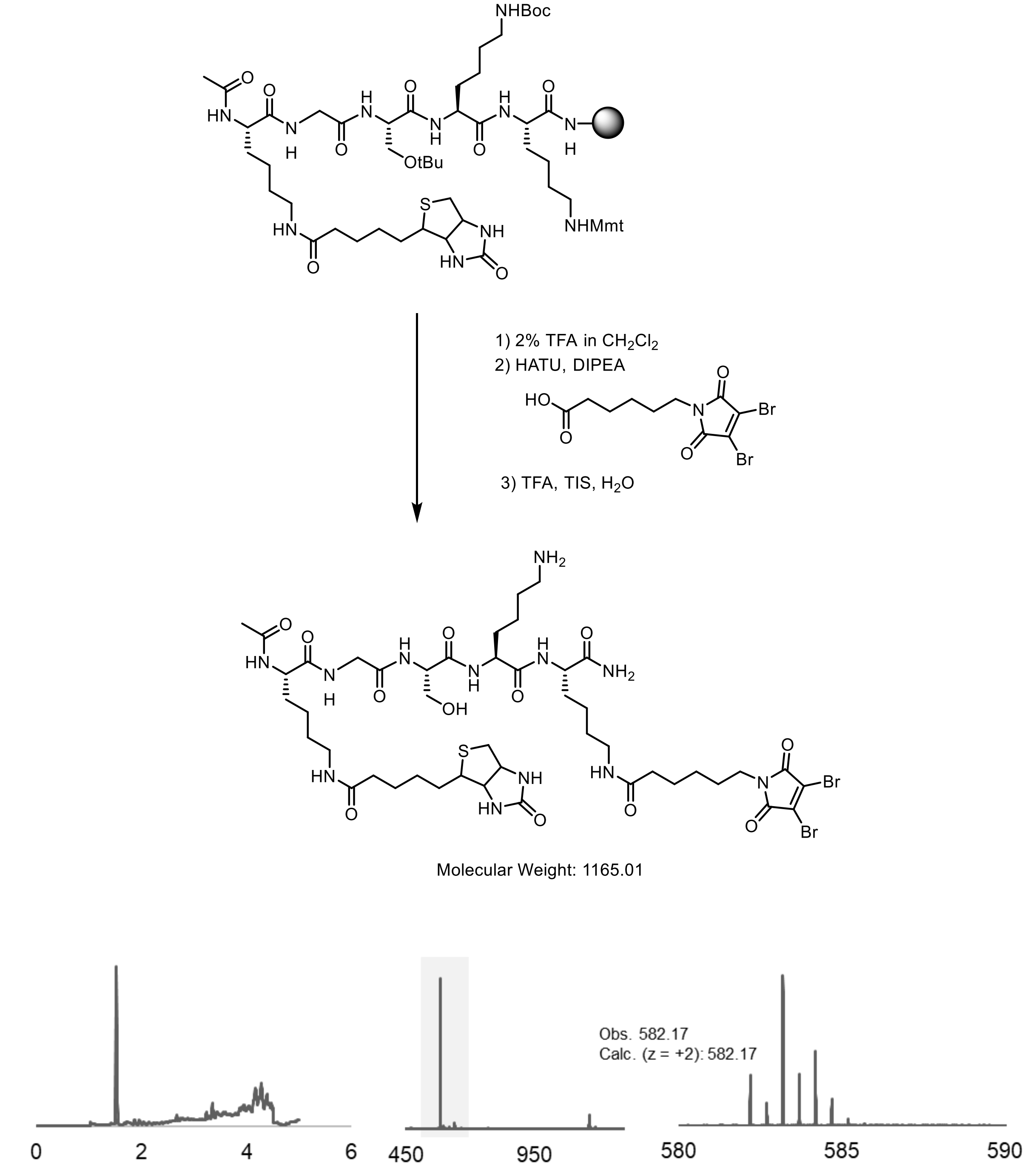

Figure S20. Synthesis scheme and LCMS trace and spectrum of probe 18.

Fmoc-Rink-amide-Protide resin (100 mg, 0.6 mmol/g loading) was incubated with piperidine (20% in DMF, 2 mL) for 10 minutes and then washed with DMF (5 x 3 mL). A HATU solution in DMF (0.4 M) was prepared and each amino acid building block was dissolved in this solution (appropriate amount to result in final amino acid concentration of 0.4 M, 500 µL). For each coupling cycle DIPEA (100 µL) was

added to the amino acid HATU mix (500 µL) for preactivation. After 30 seconds this solution was added to the resin, stirred and then incubated for 15 minutes. The coupling mixture was drained under vacuum and the resin washed with DMF (3 x 3 mL), before Fmoc removal with piperidine (20% in DMF, 2 mL) for 10 minutes followed by washes with DMF (5 x 3 mL). As last linear building block we incorporate Fmoc-Lys(Biotin)-OH, followed by Fmoc removal. The peptide was then acetylated via treatment with acetic anhydride (10% in DMF with 10% DIPEA, 2 mL, 10 minutes). After washes with DMF (5 x 3 mL) and CH_2Cl_2 (3 x 3 mL), the resin was incubated with TFA (1% in CH_2Cl_2, 1 mL, 5 x 2 min) to remove the Mmt protecting group. Dibromomaleimide-hexanoic acid (74 mg) was dissolved in DMF containing 0.4 M HATU (500 µL), activated with DIPEA (100 µL) and then added to the peptidyl resin and incubated for 30 minutes. The resin was washed with DMF (5 x 3 mL) and CH_2Cl_2 (3 x 3 mL) before cleavage and global deprotection with TFA + 5% water, 60 minutes. The probe was precipitated with ice cold ether analyzed by LCMS and used without further purification.

Synthesis of probe 19

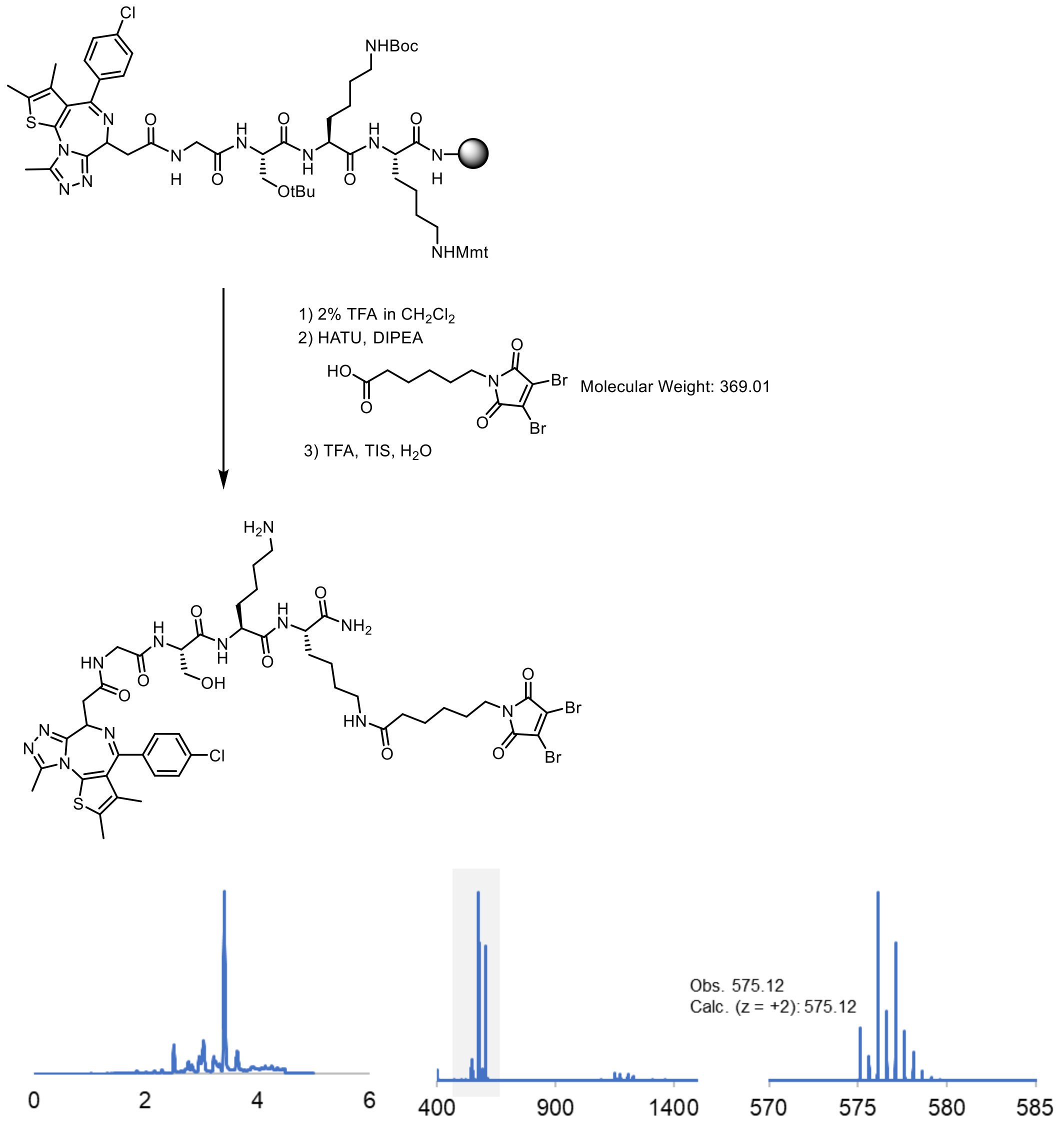

Figure S21. Synthesis scheme and LCMS trace and spectrum of probe 19.

The synthesis follows the same procedure as detailed above. As last linear building block JQ1-acid was incorporated, under the same coupling conditions as detailed for all amino acid building blocks.

Synthesis of protein conjugates 20 and 21

The protein (2 mg, 140 nmol) was dissolved in stapling buffer (500 µL, NH_4HCO_3, 100 mM, pH= 8), and then diluted with MeCN (250 µL). TCEP was dissolved in water (20 mM) and added to the stapling reaction (2 eq., 14.0 µL). Probe (18 or 19) was dissolved in MeCN (10 mM) and then added (2 eq., 28 µL) to the protein in stapling buffer. The reaction was incubated for 60 min.
When the stapling reaction was completed as determined by LC-MS the product was purified using a size exclusion (cut off 7 kDa) zeba spin column.

Protein expression and characterization

Site-directed mutagenesis

For site-directed mutagenesis the QuikChange II site-directed mutagenesis kit (Agilent Technologies) was used and primers were designed using the QuikChange® Primer Design Program provided by Agilent Technologies. Mutagenesis was performed according to the manufacturer's protocol In brief, PCR reactions were prepared using pET-30a with Omomyc as template (0.5 µL, ~25 ng), forward and reverse primers (0.6 µL each, 10 µM), MQ water (18.8 µL) as well as the contents provided by the kit: NTP mix (1 µL), 10x reaction buffer (2.5 µL) and *PfuUltra* high fidelity DNA polymerase. In some cases, when the formation of primer dimers was seen, primer concentration was reduced and 1 µL of dmso was added. PCR was performed for 22 cycles (95 °C, 30s; 55 °C, 1 minute; 68 °C, 10 minutes) followed by *Dpn I* restriction for 2 hours at 37 °C (addition of 0.5 µL at 10 U/µL)

Mutated plasmids were then incorporated into XL-1 blue competent cells. Cells were thawed on ice and 8 µL of the *Dpn I*-treated DNA were added to 50 µL of cells. The mixture was incubated on ice for 30 minutes, followed by a 45 second heat pulse at 42 °C and incubation on ice for another 2 minutes. Subsequently, 0.5 mL SOC-medium were added and the cells shaken at 250 rpm, 37 °C for 1 hour. For selection, 100 µL of the mixture were plated on one half of an LB agar plate containing gentamicin and kanamycin. The rest of the cells was spun down, the supernatant removed except for 100 µL, the cells resuspended in these 100 µL and plated on the other half of the LB agar plate. The plate was incubated at 37 °C overnight.

About 5-10 colonies were picked from selection and grown overnight in 5 mL LB media supplemented with gentamicin and kanamycin and plasmid DNA was isolated using the QIAprep Spin Miniprep kit (Qiagen, Venlo, NL) according to the manufacturer's protocol. The isolated plasmid DNA was sequenced using a sanger sequencing service and analyzed using Benchling.

Primers used for site-directed mutagenesis

Mutation	Primer sequence 5'-3' (forward, mutation in bold)
C91A	caaacttgaacagctacggaactct**gct**gcgtaaggactc
T3C	gatatcggatccatggcg**tg**cgaggagaatgtcaagag
V7C	ccatggcgaccgaggagaat**tg**caagaggcgaacac
V7C T11C	ggcgaccgaggagaat**tg**caagaggcga**tgc**cacaacgtcttggagc
V14C	gaggcgaacacacaac**tg**cttggagcgccagagg
N21C	gagcgccagaggagg**tg**cgagctaaaacggag

Primers used for sequencing

Name	Sequence 5'-3'
T7 terbis – sequencing reverse	aaccccctcaagacccg
pET upstream – sequencing forward	gatgcgtccggcgtagag

Expression of different Omomyc variants

Plasmids used were either obtained from site-directed mutagenesis of pET-30a with the omomyc gene sequence (for stapled variants termed HeloMYC) or genes as well as insertion of them by express cloning were ordered from GenScript and the plasmids used as delivered (for artificial coiled-coil and mutations in the coiled-coil termed ArtiMYC and ArgiMyc as well as NLS-HeloMYC fusions, see annex for plasmid sequences).

Plasmids were transformed into competent ArcticXpress DE3 RIL cells by heat shock according to the manufacturer's protocol. Briefly, 2 µL of 10% β mercaptoethanol were mixed with 100 µL of competent cell suspension thawed on ice and incubated for 10 minutes on ice. Next, 25 ng of plasmid DNA were added and the cells incubated for another 30 minutes on ice. The cells were then heat-shocked in a water bath for 20 seconds at 42 °C and subsequently incubated on ice for 2 minutes followed by the addition of 0.9 mL SOC media and incubation at 37 °C and 250 rpm for 1 h. Cells were then pelleted by centrifugation, 0.9 mL of the supernatant decanted and the pellet resuspended in the remaining 100 µL of media. Cells were plated for selection on LB agar with kanamycin and gentamicin and incubated at 37 °C overnight.

Single colonies were picked from the plate and cultured overnight at 37 °C and 180 rpm in 100 mL of LB media containing kanamycin and gentamycin. Next, 3x2 L of LB media containing kanamycin and gentamycin in 5 L Erlenmeyer flasks were inoculated with 25 mL of preculture and grown at 37 °C and 180 rpm until an OD of 0.8 was reached. The temperature was then set to 14 °C and protein expression induced by addition of IPTG to a final concentration of 100 µM. Protein expression was conducted overnight for 18 h after which the cells were harvested by centrifugation (5,000g, 4 °C, 12 min.), the pellet resuspended in lysis buffer (20 mM Tris-HCl pH 8, 0.5 M NaCl, 10 mM imidazole, 3 mM $MgCl_2$, freshly added 1 cOmplete EDTA-free protease inhibitor cocktail tablet per 50 mL of buffer and 0.05-0.1% DNase) and the cells lysed by pressure lysis. Cell debris was removed by ultracentrifugation (35,000 rpm, 4°C, 45 min.)

Purification of His-tagged proteins

After ultracentrifugation, the supernatant was purified using an ÄKTA start protein purification system equipped with a 5 mL HisTrap HP His-tag protein purification column (Cytiva). After washing out unbound protein with wash buffer (20 mM Tris-HCl pH 8, 0.5 M NaCl, 10 mM imidazole) the protein was eluted using a gradient of 10 mM to 500 mM imidazole in the same buffer over 40-50 column volumes of buffer. Fractions with protein were analyzed for protein content and identity by LC-MS.

General protocol for buffer exchange

The combined fractions obtained from Ni-column purification were incubated with 5 mM TCEP for 1h to break any possible formed disulfide bonds. The buffer was then exchanged by subjecting the protein to column chromatography on a Biotage® Selekt Flash Purification System equipped with a Biotage® Sfär C18 D - Duo 25g or 50g column and a stepwise gradient of 0 % MeCN in water followed by 50 to 100% MeCN in water). The combined fractions containing the protein were lyophilized, yielding the His-tagged or final protein as TFA salt.

Tag cleavage with enterokinase and subsequent purification

Lyophilized protein was dissolved at 2 mg/mL in EK cleavage buffer (200 mM Tris-HCl, pH 7.4, 0.5 M NaCl, 20 mM $CaCl_2$) and after addition of 10 u/mL enterokinase the protein was incubated overnight. The protein was then purified using an ÄKTA start protein purification system equipped with a 5 mL HisTrap HP His tag protein purification column (Cytiva) applying a gradient of 10 mM to 500 mM imidazole in elution buffer (20 mM Tris-HCl pH 8, 0.5 M NaCl, 10-500 mM imidazole). The fractions were analyzed by LC-MS and the buffer of the combined protein containing fractions was exchanged as described above, yielding the final proteins as TFA salts after lyophilization.

Electromobility shift assay (EMSA)

For EMSAs, proteins were serial diluted with water to a final volume of 10 µL. Subsequently, 5 µL of 4x EMSA buffer (final buffer concentration: 20 mM HEPES pH 8.0, 150 mM NaCl, 5% glycerol, 1 mM EDTA, 2 mM $MgCl_2$, 0.5 mg/mL of BSA, 1 mM DTT and 0.05% NP-40) followed by 5 µL of 4x FAM-labelled DNA construct (IRD700-ACC CCA C**CA CGT G**GT GCC T, final concentration 4 nM) were added.

The samples were incubated for 30 minutes at room temperature, placed on ice and incubated for another 15 minutes. Then 15 µL of the samples were loaded onto a 10% native acrylamide TBE gel which was pre run before for 1 h at 75 V at 4 °C in 0.5x TBE. Samples were run for 20 minutes at 120 V followed by 40 minutes at 100 V at 4 °C in 0.5x TBE and subsequently scanned on a Bio-Rad ChemiDoc MP machine. Bound protein signal was quantified using ImageJ and KD values were obtained using the inhibitor concentration vs. response variable slope model of nonlinear regression in GraphPad Prism 9.0.0.and the IC50 value reported as KD.

Circular Dichroism Spectroscopy

For CD measurements 1 mM protein stocks were diluted to 20 µM in Dulbecco's phosphate buffered saline (DPBS) to a final volume of 200 µL. For DNA containing samples, a 1 mM DNA stock in water was heated for 5 min to 95 °C, let cool down to RT and an equimolar amount of DNA was added to the protein samples dedicated to be measured with DNA. Additionally, a blank measurement with or without DNA was measured. Circular Dichroism (CD) samples were measured at 37 °C using a Jasco J-8151 CD spectrometer with a 1 mm path length quartz cuvette. The following parameters were used for a full wavelength scan: wavelength = 260-200 nm; Data pitch = 1 nm; scanning mode = continuous; scanning speed = 100 nm/min; response = 1, BW = 1, accumulation = 5. For CD melting curves the same samples were cooled down to 5 °C and slowly heated to 90 °C while measuring at 222 nm, with a heating speed of 2 °C/min.

For CD analysis, the blank measurement was subtracted from each spectrum and the mean residue molar ellipticity (θ, deg cm^2 dmol) was calculated using the following equation[2]:

$$[\theta] = \frac{100 * \theta_{obs}}{c * n * l}$$

With θ_{obs} in mdeg, concentration (c) in mM, peptide bonds (n) and path length of cuvette in cm.

Cell culture and cell assays
Cells were cultured at 37°C in 5% CO_2 atmosphere. Cell lines were cultured in ATCC recommended media and split twice a week before confluency was reached.

MYC reporter gene assay
The reporter gene assay was performed using Cignal reporter assay (CCS-012L, Qiagen). In brief, HEK293T cells were harvested and resuspended in OptiMEM media containing 5% FBS and 1% non-essential amino acids (NEAA) as well as penicillin/streptavidin. 40,000 cells were seeded per well in a 96-well plate and 50 µL transfection cocktail of either cignal reporter or positive or negative control reporter along with attractene transfection reagent in OptiMEM without additives was added and the cells incubated overnight. Next, media was changed to assay media (OptiMEM, 0.5% FBS, 1% NEAA, penicillin/streptavidin) and the cells were incubated for 8 hours after which the media was replaced by 75 µL assay media containing the different proteins at the required concentration and the cells were incubated with the proteins for 24 hours.
Luciferase assay was then performed using a luciferase assay kit (E2940, Promega). Cells were lysed by addition of 75 µL of DualGlo luciferase assay reagent and incubated for 15 minutes after which Luciferase luminescence was measured on a Perkin Elmer EnVision 2104 Multilabel Reader. Subsequently, 75 µL of DualGlo Stop & Glo reagent were added and the renilla luciferase luminescence measured after 15 minutes of incubation time.
Signal was normalized against cell number by calculating the ratio of firefly and renilla luminescence and eventually these signals were normalized against the untreated control. All experiments were done in technical triplicates. Data was analyzed using GraphPad Prism 9.0.0. applying the model of inhibitor concentration vs. response with variable slope model for nonlinear regression.

MYC reporter assay control for direct luciferase inhibition
For the control experiment testing direct luciferase inhibition by proteins, the cells were incubated for 24 hours in assay media and the media replaced by assay media containing the proteins directly before performing the luciferase assay as described above.

Cell proliferation assay
Cells were seeded in 100 µL of their respective media at 1500 cells/well (Figure 5d) or 2000 cells/well (Figure 5e) in a white opaque 96-well plate and let attach overnight. Media was then changed to 100 µL of media with the required protein at the appropriate concentration, the plate was covered with a membrane to prevent media evaporation and the cells were incubated with the proteins for 72 hours. Cell proliferation was then assessed using CellTiter-Glo (Promega) reading luminescence on a Perkin Elmer EnVision 2104 Multilabel Reader. All experiments were done in technical triplicates. Data was analyzed using GraphPad Prism 9.0.0.

Live cell microscopy with FITC-labelled HeloMYC and Omomyc

Hela cells were harvested and seeded at 12'000 cells/well in a 96-well plate and left to adhere overnight (4.5h in the case of 24h compound treatment). Cells were then treated with 5µM Omomyc-FITC (12) or HeloMYC-1421-FITC (13) for the desired time in full media. Subsequently, the media was aspirated cells were stained for 10 minutes with 1µg/mL Hoechst in DPBS, followed by three washes with full

media. Live cells were imaged on a Nikon Ti2 microscope equipped with a Plan Apo VC 20x DIC N2 air objective using a 405nm laser for Hoechst and a 488nm laser for fluorescein excitation.

Quantification of signal in the green channel

For signal quantification the Hoechst channel was used and ROIs were determined by thresholding from 100-255. The ROIs were then dilated by 5 units to also cover the area surrounding the nucleus and a mask was created. The mask was applied to the FITC channel and the mean fluorescence measured per image. For each condition the mean of the mean fluorescence of the no treatment control was subtracted from the individual mean fluorescence and the values normalized to the desired condition.

RNA sequencing and GSEA

In a 12-well plate 50,000 HeLa cells/well were seeded. The next day the cells were treated with either 10 µM HeloMYC-1421 or vehicle in MEM media supplemented with 10% FBS, 1% Pen/Strep and Glutamax and incubated for 72 hours. Total RNA was isolated using Qiagen RNeasy Plus mini kit and dissolved in RNAse free water. Samples were shipped to and sequencing as well as differential gene expression analysis was performed by Novogene GmbH (Planegg, Germany). Gene set enrichment analysis (GSEA) was performed with the GSEA desktop application (Broad Institute) version 4.4.0. Pre-ranked GSEA was conducted using log2FC as ranking metric and 10,000 permutations were performed.

Protein sequences

Enterokinase cleavage sites shown with |

Omomyc

MHHHHHHSSGLVPRGSGMKETAAAKFERQHMDSPDLGTDDDDK|AMADIGSMATEENVKRRTHNVLER
QRRNELKRSFFALRDQIPELENNEKAPKVVILKKATAYILSVQAETQKLISEIDLLRKQNEQLKHKLE
QLRNSCA

Omomyc T3C V7C C91A

MHHHHHHSSGLVPRGSGMKETAAAKFERQHMDSPDLGTDDDDK|AMADIGSMA**C**EEN**C**KRRTHNVLER
QRRNELKRSFFALRDQIPELENNEKAPKVVILKKATAYILSVQAETQKLISEIDLLRKQNEQLKHKLE
QLRNS**A**A

Omomyc V7C T11C C91A

MHHHHHHSSGLVPRGSGMKETAAAKFERQHMDSPDLGTDDDDK|AMADIGSMATEEN**C**KRR**C**HNVLER
QRRNELKRSFFALRDQIPELENNEKAPKVVILKKATAYILSVQAETQKLISEIDLLRKQNEQLKHKLE
QLRNS**A**A

Omomyc V7C V14C C91A

MHHHHHHSSGLVPRGSGMKETAAAKFERQHMDSPDLGTDDDDK|AMADIGSMATEEN**C**KRRTHN**C**LER
QRRNELKRSFFALRDQIPELENNEKAPKVVILKKATAYILSVQAETQKLISEIDLLRKQNEQLKHKLE
QLRNS**A**A

Omomyc V14C N21C C91A

MHHHHHHSSGLVPRGSGMKETAAAKFERQHMDSPDLGTDDDDK|AMADIGSMATEENVKRRTHN**C**LER
QRR**C**ELKRSFFALRDQIPELENNEKAPKVVILKKATAYILSVQAETQKLISEIDLLRKQNEQLKHKLE
QLRNS**A**A

Omomyc Q64R D71R Q86R C91A - ArgiMyc
MHHHHHHSSGLVPRGSGMKETAAAKFERQHMDSPDLGTDDDDK|AMADIGSMATEENVKRRTHNVLER
QRRNELKRSFFALRDQIPELENNEKAPKVVILKKATAYILSVQAET**R**KLISEI**R**LLRKQNEQLKHKLE
RLRNS**A**A

ArtiMyc
MHHHHHHSSGLVPRGSGMKETAAAKFERQHMDSPDLGTDDDDK|AMADIGSMATEENVKRRTHNVLER
QRRNELKRSFFALRDQIPELENNEKAPKVVILKKATAYILSV**KREIAALKREIAALKREIAALKRE**

NucleoMYC 16
MHHHHHHSSGLVPRGSGMKETAAAKFERQHMDSPDLGTDDDDK|AMADIGSMATEENVKRRTHN**C**LER
QRR**C**ELKRSFFALRDQIPELENNEKAPKVVILKKATAYILSVQAETQKLISEIDLLRKQNEQLKHKLE
QLRNS**A**A**PAAKRVKLD**

NucleoMYC 17
MHHHHHHSSGLVPRGSGMKETAAAKFERQHMDSPDLGTDDDDK|AMADIGS**PAAKRVKLD**MATEENVK
RRTHN**C**LERQRR**C**ELKRSFFALRDQIPELENNEKAPKVVILKKATAYILSVQAETQKLISEIDLLRKQ
NEQLKHKLEQLRNS**A**A

Plasmid sequences
pET-30a with Omomyc
TCAGAGGTTTTCACCGTCATCACCGAAACGCGCGAGGCAGCTGCGGTAAAGCTCATCAGCGTGGTCGT
GAAGCGATTCACAGATGTCTGCCTGTTCATCCGCGTCCAGCTCGTTGAGTTTCTCCAGAAGCGTTAAT
GTCTGGCTTCTGATAAAGCGGGCCATGTTAAGGGCGGTTTTTTCCTGTTTGGTCACTGATGCCTCCGT
GTAAGGGGGATTTCTGTTCATGGGGGTAATGATACCGATGAAACGAGAGAGGATGCTCACGATACGGG
TTACTGATGATGAACATGCCCGGTTACTGGAACGTTGTGAGGGTAAACAACTGGCGGTATGGATGCGG
CGGGACCAGAGAAAAATCACTCAGGGTCAATGCCAGCGCTTCGTTAATACAGATGTAGGTGTTCCACA
GGGTAGCCAGCAGCATCCTGCGATGCAGATCCGGAACATAATGGTGCAGGGCGCTGACTTCCGCGTTT
CCAGACTTTACGAAACACGGAAACCGAAGACCATTCATGTTGTTGCTCAGGTCGCAGACGTTTTGCAG
CAGCAGTCGCTTCACGTTCGCTCGCGTATCGGTGATTCATTCTGCTAACCAGTAAGGCAACCCCGCCA
GCCTAGCCGGGTCCTCAACGACAGGAGCACGATCATGCGCACCCGTGGGGCCGCCATGCCGGCGATAA
TGGCCTGCTTCTCGCCGAAACGTTTGGTGGCGGGACCAGTGACGAAGGCTTGAGCGAGGGCGTGCAAG
ATTCCGAATACCGCAAGCGACAGGCCGATCATCGTCGCGCTCCAGCGAAAGCGGTCCTCGCCGAAAAT
GACCCAGAGCGCTGCCGGCACCTGTCCTACGAGTTGCATGATAAAGAAGACAGTCATAAGTGCGGCGA
CGATAGTCATGCCCCGCGCCCACCGGAAGGAGCTGACTGGGTTGAAGGCTCTCAAGGGCATCGGTCGA
GATCCCGGTGCCTAATGAGTGAGCTAACTTACATTAATTGCGTTGCGCTCACTGCCCGCTTTCCAGTC
GGGAAACCTGTCGTGCCAGCTGCATTAATGAATCGGCCAACGCGCGGGGAGAGGCGGTTTGCGTATTG
GGCGCCAGGGTGGTTTTTCTTTTCACCAGTGAGACGGGCAACAGCTGATTGCCCTTCACCGCCTGGCC
CTGAGAGAGTTGCAGCAAGCGGTCCACGCTGGTTTGCCCCAGCAGGCGAAAATCCTGTTTGATGGTGG
TTAACGGCGGGATATAACATGAGCTGTCTTCGGTATCGTCGTATCCCACTACCGAGATGTCCGCACCA
ACGCGCAGCCCGGACTCGGTAATGGCGCGCATTGCGCCCAGCGCCATCTGATCGTTGGCAACCAGCAT
CGCAGTGGGAACGATGCCCTCATTCAGCATTTGCATGGTTTGTTGAAAACCGGACATGGCACTCCAGT
CGCCTTCCCGTTCCGCTATCGGCTGAATTTGATTGCGAGTGAGATATTTATGCCAGCCAGCCAGACGC
AGACGCGCCGAGACAGAACTTAATGGGCCCGCTAACAGCGCGATTTGCTGGTGACCCAATGCGACCAG
ATGCTCCACGCCCAGTCGCGTACCGTCTTCATGGGAGAAAATAATACTGTTGATGGGTGTCTGGTCAG
AGACATCAAGAAATAACGCCGGAACATTAGTGCAGGCAGCTTCCACAGCAATGGCATCCTGGTCATCC
AGCGGATAGTTAATGATCAGCCCACTGACGCGTTGCGCGAGAAGATTGTGCACCGCCGCTTTACAGGC
TTCGACGCCGCTTCGTTCTACCATCGACACCACCACGCTGGCACCCAGTTGATCGGCGCGAGATTTAA
TCGCCGCGACAATTTGCGACGGCGCGTGCAGGGCCAGACTGGAGGTGGCAACGCCAATCAGCAACGAC
TGTTTGCCCGCCAGTTGTTGTGCCACGCGGTTGGGAATGTAATTCAGCTCCGCCATCGCCGCTTCCAC

TTTTTCCCGCGTTTTCGCAGAAACGTGGCTGGCCTGGTTCACCACGCGGGAAACGGTCTGATAAGAGA
CACCGGCATACTCTGCGACATCGTATAACGTTACTGGTTTCACATTCACCACCCTGAATTGACTCTCT
TCCGGGCGCTATCATGCCATACCGCGAAAGGTTTTGCGCCATTCGATGGTGTCCGGGATCTCGACGCT
CTCCCTTATGCGACTCCTGCATTAGGAAGCAGCCCAGTAGTAGGTTGAGGCCGTTGAGCACCGCCGCC
GCAAGGAATGGTGCATGCAAGGAGATGGCGCCCAACAGTCCCCCGGCCACGGGGCCTGCCACCATACC
CACGCCGAAACAAGCGCTCATGAGCCCGAAGTGGCGAGCCCGATCTTCCCCATCGGTGATGTCGGCGA
TATAGGCGCCAGCAACCGCACCTGTGGCGCCGGTGATGCCGGCCACGATGCGTCCGGCGTAGAGGATC
GAGATCGATCTCGATCCCGCGAAATTAATACGACTCACTATAGGGGAATTGTGAGCGGATAACAATTC
CCCTCTAGAAATAATTTTGTTTAACTTTAAGAAGGAGATATACATATGCACCATCATCATCATCATTC
TTCTGGTCTGGTGCCACGCGGTTCTGGTATGAAAGAAACCGCTGCTGCTAAATTCGAACGCCAGCACA
TGGACAGCCCAGATCTGGGTACCGACGACGACGACAAGGCCATGGCTGATATCGGATCCATGGCGACC
GAGGAGAATGTCAAGAGGCGAACACACAACGTCTTGGAGCGCCAGAGGAGGAACGAGCTAAAACGGAG
CTTTTTTGCCCTGCGTGACCAGATCCCGGAGTTGGAAAACAATGAAAAGGCCCCCAAGGTAGTTATCC
TTAAAAAAGCCACAGCATACATCCTGTCCGTCCAAGCAGAGACGCAAAAGCTCATTTCTGAAATCGAC
TTGTTGCGGAAACAAAACGAACAGTTGAAACACAAACTTGAACAGCTACGGAACTCTTGTGCGTAAGG
ACTCGAGCACCACCACCACCACCACTGAGATCCGGCTGCTAACAAAGCCCGAAAGGAAGCTGAGTTGG
CTGCTGCCACCGCTGAGCAATAACTAGCATAACCCCTTGGGGCCTCTAAACGGGTCTTGAGGGGTTTT
TTGCTGAAAGGAGGAACTATATCCGGATTGGCGAATGGGACGCGCCCTGTAGCGGCGCATTAAGCGCG
GCGGGTGTGGTGGTTACGCGCAGCGTGACCGCTACACTTGCCAGCGCCCTAGCGCCCGCTCCTTTCGC
TTTCTTCCCTTCCTTTCTCGCCACGTTCGCCGGCTTTCCCCGTCAAGCTCTAAATCGGGGGCTCCCTT
TAGGGTTCCGATTTAGTGCTTTACGGCACCTCGACCCCAAAAAACTTGATTAGGGTGATGGTTCACGT
AGTGGGCCATCGCCCTGATAGACGGTTTTTCGCCCTTTGACGTTGGAGTCCACGTTCTTTAATAGTGG
ACTCTTGTTCCAAACTGGAACAACACTCAACCCTATCTCGGTCTATTCTTTTGATTTATAAGGGATTT
TGCCGATTTCGGCCTATTGGTTAAAAAATGAGCTGATTTAACAAAAATTTAACGCGAATTTTAACAAA
ATATTAACGTTTACAATTTCAGGTGGCACTTTTCGGGGAAATGTGCGCGGAACCCCTATTTGTTTATT
TTTCTAAATACATTCAAATATGTATCCGCTCATGAATTAATTCTTAGAAAAACTCATCGAGCATCAAA
TGAAACTGCAATTTATTCATATCAGGATTATCAATACCATATTTTTGAAAAAGCCGTTTCTGTAATGA
AGGAGAAAACTCACCGAGGCAGTTCCATAGGATGGCAAGATCCTGGTATCGGTCTGCGATTCCGACTC
GTCCAACATCAATACAACCTATTAATTTCCCCTCGTCAAAAATAAGGTTATCAAGTGAGAAATCACCA
TGAGTGACGACTGAATCCGGTGAGAATGGCAAAAGTTTATGCATTTCTTTCCAGACTTGTTCAACAGG
CCAGCCATTACGCTCGTCATCAAAATCACTCGCATCAACCAAACCGTTATTCATTCGTGATTGCGCCT
GAGCGAGACGAAATACGCGATCGCTGTTAAAAGGACAATTACAAACAGGAATCGAATGCAACCGGCGC
AGGAACACTGCCAGCGCATCAACAATATTTTCACCTGAATCAGGATATTCTTCTAATACCTGGAATGC
TGTTTTCCCGGGGATCGCAGTGGTGAGTAACCATGCATCATCAGGAGTACGGATAAAATGCTTGATGG
TCGGAAGAGGCATAAATTCCGTCAGCCAGTTTAGTCTGACCATCTCATCTGTAACATCATTGGCAACG
CTACCTTTGCCATGTTTCAGAAACAACTCTGGCGCATCGGGCTTCCCATACAATCGATAGATTGTCGC
ACCTGATTGCCCGACATTATCGCGAGCCCATTTATACCCATATAAATCAGCATCCATGTTGGAATTTA
ATCGCGGCCTAGAGCAAGACGTTTCCCGTTGAATATGGCTCATAACACCCCTTGTATTACTGTTTATG
TAAGCAGACAGTTTTATTGTTCATGACCAAAATCCCTTAACGTGAGTTTTCGTTCCACTGAGCGTCAG
ACCCCGTAGAAAAGATCAAAGGATCTTCTTGAGATCCTTTTTTTCTGCGCGTAATCTGCTGCTTGCAA
ACAAAAAAACCACCGCTACCAGCGGTGGTTTGTTTGCCGGATCAAGAGCTACCAACTCTTTTTCCGAA
GGTAACTGGCTTCAGCAGAGCGCAGATACCAAATACTGTCCTTCTAGTGTAGCCGTAGTTAGGCCACC
ACTTCAAGAACTCTGTAGCACCGCCTACATACCTCGCTCTGCTAATCCTGTTACCAGTGGCTGCTGCC
AGTGGCGATAAGTCGTGTCTTACCGGGTTGGACTCAAGACGATAGTTACCGGATAAGGCGCAGCGGTC
GGGCTGAACGGGGGGTTCGTGCACACAGCCCAGCTTGGAGCGAACGACCTACACCGAACTGAGATACC
TACAGCGTGAGCTATGAGAAAGCGCCACGCTTCCCGAAGGGAGAAAGGCGGACAGGTATCCGGTAAGC
GGCAGGGTCGGAACAGGAGAGCGCACGAGGGAGCTTCCAGGGGGAAACGCCTGGTATCTTTATAGTCC
TGTCGGGTTTCGCCACCTCTGACTTGAGCGTCGATTTTTGTGATGCTCGTCAGGGGGGCGGAGCCTAT
GGAAAAACGCCAGCAACGCGGCCTTTTTACGGTTCCTGGCCTTTTGCTGGCCTTTTGCTCACATGTTC
TTTCCTGCGTTATCCCCTGATTCTGTGGATAACCGTATTACCGCCTTTGAGTGAGCTGATACCGCTCG
CCGCAGCCGAACGACCGAGCGCAGCGAGTCAGTGAGCGAGGAAGCGGAAGAGCGCCTGATGCGGTATT
TTCTCCTTACGCATCTGTGCGGTATTTCACACCGCATATATGGTGCACTCTCAGTACAATCTGCTCTG

ATGCCGCATAGTTAAGCCAGTATACACTCCGCTATCGCTACGTGACTGGGTCATGGCTGCGCCCCGAC
ACCCGCCAACACCCGCTGACGCGCCCTGACGGGCTTGTCTGCTCCCGGCATCCGCTTACAGACAAGCT
GTGACCGTCTCCGGGAGCTGCATGTG

pET-30a with artificial coiled-coil

TGGCGAATGGGACGCGCCCTGTAGCGGCGCATTAAGCGCGGCGGGTGTGGTGGTTACGCGCAGCGTGA
CCGCTACACTTGCCAGCGCCCTAGCGCCCGCTCCTTTCGCTTTCTTCCCTTCCTTTCTCGCCACGTTC
GCCGGCTTTCCCCGTCAAGCTCTAAATCGGGGGCTCCCTTTAGGGTTCCGATTTAGTGCTTTACGGCA
CCTCGACCCCAAAAAACTTGATTAGGGTGATGGTTCACGTAGTGGGCCATCGCCCTGATAGACGGTTT
TTCGCCCTTTGACGTTGGAGTCCACGTTCTTTAATAGTGGACTCTTGTTCCAAACTGGAACAACACTC
AACCCTATCTCGGTCTATTCTTTTGATTTATAAGGGATTTTGCCGATTTCGGCCTATTGGTTAAAAAA
TGAGCTGATTTAACAAAAATTTAACGCGAATTTTAACAAAATATTAACGTTTACAATTTCAGGTGGCA
CTTTTCGGGGAAATGTGCGCGGAACCCCTATTTGTTTATTTTTCTAAATACATTCAAATATGTATCCG
CTCATGAATTAATTCTTAGAAAAACTCATCGAGCATCAAATGAAACTGCAATTTATTCATATCAGGAT
TATCAATACCATATTTTTGAAAAAGCCGTTTCTGTAATGAAGGAGAAAACTCACCGAGGCAGTTCCAT
AGGATGGCAAGATCCTGGTATCGGTCTGCGATTCCGACTCGTCCAACATCAATACAACCTATTAATTT
CCCCTCGTCAAAAATAAGGTTATCAAGTGAGAAATCACCATGAGTGACGACTGAATCCGGTGAGAATG
GCAAAAGTTTATGCATTTCTTTCCAGACTTGTTCAACAGGCCAGCCATTACGCTCGTCATCAAAATCA
CTCGCATCAACCAAACCGTTATTCATTCGTGATTGCGCCTGAGCGAGACGAAATACGCGATCGCTGTT
AAAAGGACAATTACAAACAGGAATCGAATGCAACCGGCGCAGGAACACTGCCAGCGCATCAACAATAT
TTTCACCTGAATCAGGATATTCTTCTAATACCTGGAATGCTGTTTTCCCGGGGATCGCAGTGGTGAGT
AACCATGCATCATCAGGAGTACGGATAAAATGCTTGATGGTCGGAAGAGGCATAAATTCCGTCAGCCA
GTTTAGTCTGACCATCTCATCTGTAACATCATTGGCAACGCTACCTTTGCCATGTTTCAGAAACAACT
CTGGCGCATCGGGCTTCCCATACAATCGATAGATTGTCGCACCTGATTGCCCGACATTATCGCGAGCC
CATTTATACCCATATAAATCAGCATCCATGTTGGAATTTAATCGCGGCCTAGAGCAAGACGTTTCCCG
TTGAATATGGCTCATAACACCCCTTGTATTACTGTTTATGTAAGCAGACAGTTTTATTGTTCATGACC
AAAATCCCTTAACGTGAGTTTTCGTTCCACTGAGCGTCAGACCCCGTAGAAAAGATCAAAGGATCTTC
TTGAGATCCTTTTTTTCTGCGCGTAATCTGCTGCTTGCAAACAAAAAAACCACCGCTACCAGCGGTGG
TTTGTTTGCCGGATCAAGAGCTACCAACTCTTTTTCCGAAGGTAACTGGCTTCAGCAGAGCGCAGATA
CCAAATACTGTCCTTCTAGTGTAGCCGTAGTTAGGCCACCACTTCAAGAACTCTGTAGCACCGCCTAC
ATACCTCGCTCTGCTAATCCTGTTACCAGTGGCTGCTGCCAGTGGCGATAAGTCGTGTCTTACCGGGT
TGGACTCAAGACGATAGTTACCGGATAAGGCGCAGCGGTCGGGCTGAACGGGGGGTTCGTGCACACAG
CCCAGCTTGGAGCGAACGACCTACACCGAACTGAGATACCTACAGCGTGAGCTATGAGAAAGCGCCAC
GCTTCCCGAAGGGAGAAAGGCGGACAGGTATCCGGTAAGCGGCAGGGTCGGAACAGGAGAGCGCACGA
GGGAGCTTCCAGGGGGAAACGCCTGGTATCTTTATAGTCCTGTCGGGTTTCGCCACCTCTGACTTGAG
CGTCGATTTTTGTGATGCTCGTCAGGGGGGCGGAGCCTATGGAAAAACGCCAGCAACGCGGCCTTTTT
ACGGTTCCTGGCCTTTTGCTGGCCTTTTGCTCACATGTTCTTTCCTGCGTTATCCCCTGATTCTGTGG
ATAACCGTATTACCGCCTTTGAGTGAGCTGATACCGCTCGCCGCAGCCGAACGACCGAGCGCAGCGAG
TCAGTGAGCGAGGAAGCGGAAGAGCGCCTGATGCGGTATTTTCTCCTTACGCATCTGTGCGGTATTTC
ACACCGCATATATGGTGCACTCTCAGTACAATCTGCTCTGATGCCGCATAGTTAAGCCAGTATACACT
CCGCTATCGCTACGTGACTGGGTCATGGCTGCGCCCCGACACCCGCCAACACCCGCTGACGCGCCCTG
ACGGGCTTGTCTGCTCCCGGCATCCGCTTACAGACAAGCTGTGACCGTCTCCGGGAGCTGCATGTGTC
AGAGGTTTTCACCGTCATCACCGAAACGCGCGAGGCAGCTGCGGTAAAGCTCATCAGCGTGGTCGTGA
AGCGATTCACAGATGTCTGCCTGTTCATCCGCGTCCAGCTCGTTGAGTTTCTCCAGAAGCGTTAATGT
CTGGCTTCTGATAAAGCGGGCCATGTTAAGGGCGGTTTTTTCCTGTTTGGTCACTGATGCCTCCGTGT
AAGGGGGATTTCTGTTCATGGGGGTAATGATACCGATGAAACGAGAGAGGATGCTCACGATACGGGTT
ACTGATGATGAACATGCCCGGTTACTGGAACGTTGTGAGGGTAAACAACTGGCGGTATGGATGCGGCG
GGACCAGAGAAAAATCACTCAGGGTCAATGCCAGCGCTTCGTTAATACAGATGTAGGTGTTCCACAGG
GTAGCCAGCAGCATCCTGCGATGCAGATCCGGAACATAATGGTGCAGGGCGCTGACTTCCGCGTTTCC
AGACTTTACGAAACACGGAAACCGAAGACCATTCATGTTGTTGCTCAGGTCGCAGACGTTTTGCAGCA
GCAGTCGCTTCACGTTCGCTCGCGTATCGGTGATTCATTCTGCTAACCAGTAAGGCAACCCCGCCAGC

CTAGCCGGGTCCTCAACGACAGGAGCACGATCATGCGCACCCGTGGGGCCGCCATGCCGGCGATAATG
GCCTGCTTCTCGCCGAAACGTTTGGTGGCGGGACCAGTGACGAAGGCTTGAGCGAGGGCGTGCAAGAT
TCCGAATACCGCAAGCGACAGGCCGATCATCGTCGCGCTCCAGCGAAAGCGGTCCTCGCCGAAAATGA
CCCAGAGCGCTGCCGGCACCTGTCCTACGAGTTGCATGATAAAGAAGACAGTCATAAGTGCGGCGACG
ATAGTCATGCCCCGCGCCCACCGGAAGGAGCTGACTGGGTTGAAGGCTCTCAAGGGCATCGGTCGAGA
TCCCGGTGCCTAATGAGTGAGCTAACTTACATTAATTGCGTTGCGCTCACTGCCCGCTTTCCAGTCGG
GAAACCTGTCGTGCCAGCTGCATTAATGAATCGGCCAACGCGCGGGGAGAGGCGGTTTGCGTATTGGG
CGCCAGGGTGGTTTTTCTTTTCACCAGTGAGACGGGCAACAGCTGATTGCCCTTCACCGCCTGGCCCT
GAGAGAGTTGCAGCAAGCGGTCCACGCTGGTTTGCCCCAGCAGGCGAAAATCCTGTTTGATGGTGGTT
AACGGCGGGATATAACATGAGCTGTCTTCGGTATCGTCGTATCCCACTACCGAGATGTCCGCACCAAC
GCGCAGCCCGGACTCGGTAATGGCGCGCATTGCGCCCAGCGCCATCTGATCGTTGGCAACCAGCATCG
CAGTGGGAACGATGCCCTCATTCAGCATTTGCATGGTTTGTTGAAAACCGGACATGGCACTCCAGTCG
CCTTCCCGTTCCGCTATCGGCTGAATTTGATTGCGAGTGAGATATTTATGCCAGCCAGCCAGACGCAG
ACGCGCCGAGACAGAACTTAATGGGCCCGCTAACAGCGCGATTTGCTGGTGACCCAATGCGACCAGAT
GCTCCACGCCCAGTCGCGTACCGTCTTCATGGGAGAAAATAATACTGTTGATGGGTGTCTGGTCAGAG
ACATCAAGAAATAACGCCGGAACATTAGTGCAGGCAGCTTCCACAGCAATGGCATCCTGGTCATCCAG
CGGATAGTTAATGATCAGCCCACTGACGCGTTGCGCGAGAAGATTGTGCACCGCCGCTTTACAGGCTT
CGACGCCGCTTCGTTCTACCATCGACACCACCACGCTGGCACCCAGTTGATCGGCGCGAGATTTAATC
GCCGCGACAATTTGCGACGGCGCGTGCAGGGCCAGACTGGAGGTGGCAACGCCAATCAGCAACGACTG
TTTGCCCGCCAGTTGTTGTGCCACGCGGTTGGGAATGTAATTCAGCTCCGCCATCGCCGCTTCCACTT
TTTCCCGCGTTTTCGCAGAAACGTGGCTGGCCTGGTTCACCACGCGGGAAACGGTCTGATAAGAGACA
CCGGCATACTCTGCGACATCGTATAACGTTACTGGTTTCACATTCACCACCCTGAATTGACTCTCTTC
CGGGCGCTATCATGCCATACCGCGAAAGGTTTTGCGCCATTCGATGGTGTCCGGGATCTCGACGCTCT
CCCTTATGCGACTCCTGCATTAGGAAGCAGCCCAGTAGTAGGTTGAGGCCGTTGAGCACCGCCGCCGC
AAGGAATGGTGCATGCAAGGAGATGGCGCCCAACAGTCCCCCGGCCACGGGGCCTGCCACCATACCCA
CGCCGAAACAAGCGCTCATGAGCCCGAAGTGGCGAGCCCGATCTTCCCCATCGGTGATGTCGGCGATA
TAGGCGCCAGCAACCGCACCTGTGGCGCCGGTGATGCCGGCCACGATGCGTCCGGCGTAGAGGATCGA
GATCGATCTCGATCCCGCGAAATTAATACGACTCACTATAGGGGAATTGTGAGCGGATAACAATTCCC
CTCTAGAAATAATTTTGTTTAACTTTAAGAAGGAGATATACATATGCACCATCATCATCATCATTCTT
CTGGTCTGGTGCCACGCGGTTCTGGTATGAAAGAAACCGCTGCTGCTAAATTCGAACGCCAGCACATG
GACAGCCCAGATCTGGGTACCGACGACGACGACAAGGCCATGGCTGATATCGGATCCATGGCTACAGA
GGAAAATGTAAAAAGAAGGACGCACAACGTTCTGGAGCGCCAGCGTCGTAATGAACTGAAGCGTTCCT
TCTTTGCATTACGTGACCAAATTCCGGAACTGGAGAACAACGAAAAAGCTCCGAAAGTGGTGATTCTG
AAGAAAGCTACCGCATACATTCTGAGCGTTAAACGTGAGATCGCGGCGTTGAAGCGCGAAATCGCGGC
GTTGAAGCGCGAGATCGCCGCGCTCAAGAGAGAGTAAGGACTCGAGCACCACCACCACCACCACTGAG
ATCCGGCTGCTAACAAAGCCCGAAAGGAAGCTGAGTTGGCTGCTGCCACCGCTGAGCAATAACTAGCA
TAACCCCTTGGGGCCTCTAAACGGGTCTTGAGGGGTTTTTTGCTGAAAGGAGGAACTATATCCGGAT

pET-30a with natural zipper domain Q64R D71R Q86R

TAAGCAGACAGTTTTATTGTTCATGACCAAAATCCCTTAACGTGAGTTTTCGTTCCACTGAGCGTCAG
ACCCCGTAGAAAAGATCAAAGGATCTTCTTGAGATCCTTTTTTTCTGCGCGTAATCTGCTGCTTGCAA
ACAAAAAAACCACCGCTACCAGCGGTGGTTTGTTTGCCGGATCAAGAGCTACCAACTCTTTTTCCGAA
GGTAACTGGCTTCAGCAGAGCGCAGATACCAAATACTGTCCTTCTAGTGTAGCCGTAGTTAGGCCACC
ACTTCAAGAACTCTGTAGCACCGCCTACATACCTCGCTCTGCTAATCCTGTTACCAGTGGCTGCTGCC
AGTGGCGATAAGTCGTGTCTTACCGGGTTGGACTCAAGACGATAGTTACCGGATAAGGCGCAGCGGTC
GGGCTGAACGGGGGGTTCGTGCACACAGCCCAGCTTGGAGCGAACGACCTACACCGAACTGAGATACC
TACAGCGTGAGCTATGAGAAAGCGCCACGCTTCCCGAAGGGAGAAAGGCGGACAGGTATCCGGTAAGC
GGCAGGGTCGGAACAGGAGAGCGCACGAGGGAGCTTCCAGGGGGAAACGCCTGGTATCTTTATAGTCC
TGTCGGGTTTCGCCACCTCTGACTTGAGCGTCGATTTTTGTGATGCTCGTCAGGGGGGCGGAGCCTAT
GGAAAAACGCCAGCAACGCGGCCTTTTTACGGTTCCTGGCCTTTTGCTGGCCTTTTGCTCACATGTTC
TTTCCTGCGTTATCCCCTGATTCTGTGGATAACCGTATTACCGCCTTTGAGTGAGCTGATACCGCTCG

CCGCAGCCGAACGACCGAGCGCAGCGAGTCAGTGAGCGAGGAAGCGGAAGAGCGCCTGATGCGGTATT
TTCTCCTTACGCATCTGTGCGGTATTTCACACCGCATATATGGTGCACTCTCAGTACAATCTGCTCTG
ATGCCGCATAGTTAAGCCAGTATACACTCCGCTATCGCTACGTGACTGGGTCATGGCTGCGCCCCGAC
ACCCGCCAACACCCGCTGACGCGCCCTGACGGGCTTGTCTGCTCCCGGCATCCGCTTACAGACAAGCT
GTGACCGTCTCCGGGAGCTGCATGTGTCAGAGGTTTTCACCGTCATCACCGAAACGCGCGAGGCAGCT
GCGGTAAAGCTCATCAGCGTGGTCGTGAAGCGATTCACAGATGTCTGCCTGTTCATCCGCGTCCAGCT
CGTTGAGTTTCTCCAGAAGCGTTAATGTCTGGCTTCTGATAAAGCGGGCCATGTTAAGGGCGGTTTTT
TCCTGTTTGGTCACTGATGCCTCCGTGTAAGGGGGATTTCTGTTCATGGGGGTAATGATACCGATGAA
ACGAGAGAGGATGCTCACGATACGGGTTACTGATGATGAACATGCCCGGTTACTGGAACGTTGTGAGG
GTAAACAACTGGCGGTATGGATGCGGCGGGACCAGAGAAAAATCACTCAGGGTCAATGCCAGCGCTTC
GTTAATACAGATGTAGGTGTTCCACAGGGTAGCCAGCAGCATCCTGCGATGCAGATCCGGAACATAAT
GGTGCAGGGCGCTGACTTCCGCGTTTCCAGACTTTACGAAACACGGAAACCGAAGACCATTCATGTTG
TTGCTCAGGTCGCAGACGTTTTGCAGCAGCAGTCGCTTCACGTTCGCTCGCGTATCGGTGATTCATTC
TGCTAACCAGTAAGGCAACCCCGCCAGCCTAGCCGGGTCCTCAACGACAGGAGCACGATCATGCGCAC
CCGTGGGGCCGCCATGCCGGCGATAATGGCCTGCTTCTCGCCGAAACGTTTGGTGGCGGGACCAGTGA
CGAAGGCTTGAGCGAGGGCGTGCAAGATTCCGAATACCGCAAGCGACAGGCCGATCATCGTCGCGCTC
CAGCGAAAGCGGTCCTCGCCGAAAATGACCCAGAGCGCTGCCGGCACCTGTCCTACGAGTTGCATGAT
AAAGAAGACAGTCATAAGTGCGGCGACGATAGTCATGCCCCGCGCCCACCGGAAGGAGCTGACTGGGT
TGAAGGCTCTCAAGGGCATCGGTCGAGATCCCGGTGCCTAATGAGTGAGCTAACTTACATTAATTGCG
TTGCGCTCACTGCCCGCTTTCCAGTCGGGAAACCTGTCGTGCCAGCTGCATTAATGAATCGGCCAACG
CGCGGGGAGAGGCGGTTTGCGTATTGGGCGCCAGGGTGGTTTTTCTTTTCACCAGTGAGACGGGCAAC
AGCTGATTGCCCTTCACCGCCTGGCCCTGAGAGAGTTGCAGCAAGCGGTCCACGCTGGTTTGCCCCAG
CAGGCGAAAATCCTGTTTGATGGTGGTTAACGGCGGGATATAACATGAGCTGTCTTCGGTATCGTCGT
ATCCCACTACCGAGATGTCCGCACCAACGCGCAGCCCGGACTCGGTAATGGCGCGCATTGCGCCCAGC
GCCATCTGATCGTTGGCAACCAGCATCGCAGTGGGAACGATGCCCTCATTCAGCATTTGCATGGTTTG
TTGAAAACCGGACATGGCACTCCAGTCGCCTTCCCGTTCCGCTATCGGCTGAATTTGATTGCGAGTGA
GATATTTATGCCAGCCAGCCAGACGCAGACGCGCCGAGACAGAACTTAATGGGCCCGCTAACAGCGCG
ATTTGCTGGTGACCCAATGCGACCAGATGCTCCACGCCCAGTCGCGTACCGTCTTCATGGGAGAAAAT
AATACTGTTGATGGGTGTCTGGTCAGAGACATCAAGAAATAACGCCGGAACATTAGTGCAGGCAGCTT
CCACAGCAATGGCATCCTGGTCATCCAGCGGATAGTTAATGATCAGCCCACTGACGCGTTGCGCGAGA
AGATTGTGCACCGCCGCTTTACAGGCTTCGACGCCGCTTCGTTCTACCATCGACACCACCACGCTGGC
ACCCAGTTGATCGGCGCGAGATTTAATCGCCGCGACAATTTGCGACGGCGCGTGCAGGGCCAGACTGG
AGGTGGCAACGCCAATCAGCAACGACTGTTTGCCCGCCAGTTGTTGTGCCACGCGGTTGGGAATGTAA
TTCAGCTCCGCCATCGCCGCTTCCACTTTTTCCCGCGTTTTCGCAGAAACGTGGCTGGCCTGGTTCAC
CACGCGGGAAACGGTCTGATAAGAGACACCGGCATACTCTGCGACATCGTATAACGTTACTGGTTTCA
CATTCACCACCCTGAATTGACTCTCTTCCGGGCGCTATCATGCCATACCGCGAAAGGTTTTGCGCCAT
TCGATGGTGTCCGGGATCTCGACGCTCTCCCTTATGCGACTCCTGCATTAGGAAGCAGCCCAGTAGTA
GGTTGAGGCCGTTGAGCACCGCCGCCGCAAGGAATGGTGCATGCAAGGAGATGGCGCCCAACAGTCCC
CCGGCCACGGGGCCTGCCACCATACCCACGCCGAAACAAGCGCTCATGAGCCCGAAGTGGCGAGCCCG
ATCTTCCCCATCGGTGATGTCGGCGATATAGGCGCCAGCAACCGCACCTGTGGCGCCGGTGATGCCGG
CCACGATGCGTCCGGCGTAGAGGATCGAGATCGATCTCGATCCCGCGAAATTAATACGACTCACTATA
GGGGAATTGTGAGCGGATAACAATTCCCCTCTAGAAATAATTTTGTTTAACTTTAAGAAGGAGATATA
CATATGCACCATCATCATCATCATTCTTCTGGTCTGGTGCCACGCGGTTCTGGTATGAAAGAAACCGC
TGCTGCTAAATTCGAACGCCAGCACATGGACAGCCCAGATCTGGGTACCGACGACGACGACAAGGCCA
TGGCTGATATCGGATCCATGGCTACAGAGGAAAATGTAAAAAGAAGGACGCATAACGTGCTGGAGCGC
CAGCGTCGTAATGAGCTCAAACGTTCTTTTTTCGCCCTGAGAGACCAAATCCCGGAATTGGAGAACAA
CGAAAAAGCTCCGAAAGTTGTGATCTTAAAGAAGGCGACCGCATACATCCTGAGCGTTCAGGCGGAAA
CCCGTAAGCTGATTAGCGAAATTCGTTTGCTGCGCAAGCAAAACGAGCAGCTGAAGCACAAATTGGAG
CGTCTGCGCAATTCCGCGGCGTAAGGACTCGAGCACCACCACCACCACCACTGAGATCCGGCTGCTAA
CAAAGCCCGAAAGGAAGCTGAGTTGGCTGCTGCCACCGCTGAGCAATAACTAGCATAACCCCTTGGGG
CCTCTAAACGGGTCTTGAGGGGTTTTTTGCTGAAAGGAGGAACTATATCCGGATTGGCGAATGGGACG
CGCCCTGTAGCGGCGCATTAAGCGCGGCGGGTGTGGTGGTTACGCGCAGCGTGACCGCTACACTTGCC

AGCGCCCTAGCGCCCGCTCCTTTCGCTTTCTTCCCTTCCTTTCTCGCCACGTTCGCCGGCTTTCCCCG
TCAAGCTCTAAATCGGGGGCTCCCTTTAGGGTTCCGATTTAGTGCTTTACGGCACCTCGACCCCAAAA
AACTTGATTAGGGTGATGGTTCACGTAGTGGGCCATCGCCCTGATAGACGGTTTTTCGCCCTTTGACG
TTGGAGTCCACGTTCTTTAATAGTGGACTCTTGTTCCAAACTGGAACAACACTCAACCCTATCTCGGT
CTATTCTTTTGATTTATAAGGGATTTTGCCGATTTCGGCCTATTGGTTAAAAAATGAGCTGATTTAAC
AAAAATTTAACGCGAATTTTAACAAAATATTAACGTTTACAATTTCAGGTGGCACTTTTCGGGGAAAT
GTGCGCGGAACCCCTATTTGTTTATTTTTCTAAATACATTCAAATATGTATCCGCTCATGAATTAATT
CTTAGAAAAACTCATCGAGCATCAAATGAAACTGCAATTTATTCATATCAGGATTATCAATACCATAT
TTTTGAAAAAGCCGTTTCTGTAATGAAGGAGAAAACTCACCGAGGCAGTTCCATAGGATGGCAAGATC
CTGGTATCGGTCTGCGATTCCGACTCGTCCAACATCAATACAACCTATTAATTTCCCCTCGTCAAAAA
TAAGGTTATCAAGTGAGAAATCACCATGAGTGACGACTGAATCCGGTGAGAATGGCAAAAGTTTATGC
ATTTCTTTCCAGACTTGTTCAACAGGCCAGCCATTACGCTCGTCATCAAAATCACTCGCATCAACCAA
ACCGTTATTCATTCGTGATTGCGCCTGAGCGAGACGAAATACGCGATCGCTGTTAAAAGGACAATTAC
AAACAGGAATCGAATGCAACCGGCGCAGGAACACTGCCAGCGCATCAACAATATTTTCACCTGAATCA
GGATATTCTTCTAATACCTGGAATGCTGTTTTCCCGGGGATCGCAGTGGTGAGTAACCATGCATCATC
AGGAGTACGGATAAAATGCTTGATGGTCGGAAGAGGCATAAATTCCGTCAGCCAGTTTAGTCTGACCA
TCTCATCTGTAACATCATTGGCAACGCTACCTTTGCCATGTTTCAGAAACAACTCTGGCGCATCGGGC
TTCCCATACAATCGATAGATTGTCGCACCTGATTGCCCGACATTATCGCGAGCCCATTTATACCCATA
TAAATCAGCATCCATGTTGGAATTTAATCGCGGCCTAGAGCAAGACGTTTCCCGTTGAATATGGCTCA
TAACACCCCTTGTATTACTGTTTATG

pET30a with NucleoMYC 16

TGGCGAATGGGACGCGCCCTGTAGCGGCGCATTAAGCGCGGCGGGTGTGGTGGTTACGCGCAGCGTGA
CCGCTACACTTGCCAGCGCCCTAGCGCCCGCTCCTTTCGCTTTCTTCCCTTCCTTTCTCGCCACGTTC
GCCGGCTTTCCCCGTCAAGCTCTAAATCGGGGGCTCCCTTTAGGGTTCCGATTTAGTGCTTTACGGCA
CCTCGACCCCAAAAAACTTGATTAGGGTGATGGTTCACGTAGTGGGCCATCGCCCTGATAGACGGTTT
TTCGCCCTTTGACGTTGGAGTCCACGTTCTTTAATAGTGGACTCTTGTTCCAAACTGGAACAACACTC
AACCCTATCTCGGTCTATTCTTTTGATTTATAAGGGATTTTGCCGATTTCGGCCTATTGGTTAAAAAA
TGAGCTGATTTAACAAAAATTTAACGCGAATTTTAACAAAATATTAACGTTTACAATTTCAGGTGGCA
CTTTTCGGGGAAATGTGCGCGGAACCCCTATTTGTTTATTTTTCTAAATACATTCAAATATGTATCCG
CTCATGAATTAATTCTTAGAAAAACTCATCGAGCATCAAATGAAACTGCAATTTATTCATATCAGGAT
TATCAATACCATATTTTTGAAAAAGCCGTTTCTGTAATGAAGGAGAAAACTCACCGAGGCAGTTCCAT
AGGATGGCAAGATCCTGGTATCGGTCTGCGATTCCGACTCGTCCAACATCAATACAACCTATTAATTT
CCCCTCGTCAAAAATAAGGTTATCAAGTGAGAAATCACCATGAGTGACGACTGAATCCGGTGAGAATG
GCAAAAGTTTATGCATTTCTTTCCAGACTTGTTCAACAGGCCAGCCATTACGCTCGTCATCAAAATCA
CTCGCATCAACCAAACCGTTATTCATTCGTGATTGCGCCTGAGCGAGACGAAATACGCGATCGCTGTT
AAAAGGACAATTACAAACAGGAATCGAATGCAACCGGCGCAGGAACACTGCCAGCGCATCAACAATAT
TTTCACCTGAATCAGGATATTCTTCTAATACCTGGAATGCTGTTTTCCCGGGGATCGCAGTGGTGAGT
AACCATGCATCATCAGGAGTACGGATAAAATGCTTGATGGTCGGAAGAGGCATAAATTCCGTCAGCCA
GTTTAGTCTGACCATCTCATCTGTAACATCATTGGCAACGCTACCTTTGCCATGTTTCAGAAACAACT
CTGGCGCATCGGGCTTCCCATACAATCGATAGATTGTCGCACCTGATTGCCCGACATTATCGCGAGCC
CATTTATACCCATATAAATCAGCATCCATGTTGGAATTTAATCGCGGCCTAGAGCAAGACGTTTCCCG
TTGAATATGGCTCATAACACCCCTTGTATTACTGTTTATGTAAGCAGACAGTTTTATTGTTCATGACC
AAAATCCCTTAACGTGAGTTTTCGTTCCACTGAGCGTCAGACCCCGTAGAAAAGATCAAAGGATCTTC
TTGAGATCCTTTTTTTCTGCGCGTAATCTGCTGCTTGCAAACAAAAAAACCACCGCTACCAGCGGTGG
TTTGTTTGCCGGATCAAGAGCTACCAACTCTTTTTCCGAAGGTAACTGGCTTCAGCAGAGCGCAGATA
CCAAATACTGTCCTTCTAGTGTAGCCGTAGTTAGGCCACCACTTCAAGAACTCTGTAGCACCGCCTAC
ATACCTCGCTCTGCTAATCCTGTTACCAGTGGCTGCTGCCAGTGGCGATAAGTCGTGTCTTACCGGGT
TGGACTCAAGACGATAGTTACCGGATAAGGCGCAGCGGTCGGGCTGAACGGGGGGTTCGTGCACACAG
CCCAGCTTGGAGCGAACGACCTACACCGAACTGAGATACCTACAGCGTGAGCTATGAGAAAGCGCCAC
GCTTCCCGAAGGGAGAAAGGCGGACAGGTATCCGGTAAGCGGCAGGGTCGGAACAGGAGAGCGCACGA
GGGAGCTTCCAGGGGGAAACGCCTGGTATCTTTATAGTCCTGTCGGGTTTCGCCACCTCTGACTTGAG

CGTCGATTTTTGTGATGCTCGTCAGGGGGGCGGAGCCTATGGAAAAACGCCAGCAACGCGGCCTTTTT
ACGGTTCCTGGCCTTTTGCTGGCCTTTTGCTCACATGTTCTTTCCTGCGTTATCCCCTGATTCTGTGG
ATAACCGTATTACCGCCTTTGAGTGAGCTGATACCGCTCGCCGCAGCCGAACGACCGAGCGCAGCGAG
TCAGTGAGCGAGGAAGCGGAAGAGCGCCTGATGCGGTATTTTCTCCTTACGCATCTGTGCGGTATTTC
ACACCGCATATATGGTGCACTCTCAGTACAATCTGCTCTGATGCCGCATAGTTAAGCCAGTATACACT
CCGCTATCGCTACGTGACTGGGTCATGGCTGCGCCCCGACACCCGCCAACACCCGCTGACGCGCCCTG
ACGGGCTTGTCTGCTCCCGGCATCCGCTTACAGACAAGCTGTGACCGTCTCCGGGAGCTGCATGTGTC
AGAGGTTTTCACCGTCATCACCGAAACGCGCGAGGCAGCTGCGGTAAAGCTCATCAGCGTGGTCGTGA
AGCGATTCACAGATGTCTGCCTGTTCATCCGCGTCCAGCTCGTTGAGTTTCTCCAGAAGCGTTAATGT
CTGGCTTCTGATAAAGCGGGCCATGTTAAGGGCGGTTTTTTCCTGTTTGGTCACTGATGCCTCCGTGT
AAGGGGGATTTCTGTTCATGGGGGTAATGATACCGATGAAACGAGAGAGGATGCTCACGATACGGGTT
ACTGATGATGAACATGCCCGGTTACTGGAACGTTGTGAGGGTAAACAACTGGCGGTATGGATGCGGCG
GGACCAGAGAAAAATCACTCAGGGTCAATGCCAGCGCTTCGTTAATACAGATGTAGGTGTTCCACAGG
GTAGCCAGCAGCATCCTGCGATGCAGATCCGGAACATAATGGTGCAGGGCGCTGACTTCCGCGTTTCC
AGACTTTACGAAACACGGAAACCGAAGACCATTCATGTTGTTGCTCAGGTCGCAGACGTTTTGCAGCA
GCAGTCGCTTCACGTTCGCTCGCGTATCGGTGATTCATTCTGCTAACCAGTAAGGCAACCCCGCCAGC
CTAGCCGGGTCCTCAACGACAGGAGCACGATCATGCGCACCCGTGGGGCCGCCATGCCGGCGATAATG
GCCTGCTTCTCGCCGAAACGTTTGGTGGCGGGACCAGTGACGAAGGCTTGAGCGAGGGCGTGCAAGAT
TCCGAATACCGCAAGCGACAGGCCGATCATCGTCGCGCTCCAGCGAAAGCGGTCCTCGCCGAAAATGA
CCCAGAGCGCTGCCGGCACCTGTCCTACGAGTTGCATGATAAAGAAGACAGTCATAAGTGCGGCGACG
ATAGTCATGCCCCGCGCCCACCGGAAGGAGCTGACTGGGTTGAAGGCTCTCAAGGGCATCGGTCGAGA
TCCCGGTGCCTAATGAGTGAGCTAACTTACATTAATTGCGTTGCGCTCACTGCCCGCTTTCCAGTCGG
GAAACCTGTCGTGCCAGCTGCATTAATGAATCGGCCAACGCGCGGGGAGAGGCGGTTTGCGTATTGGG
CGCCAGGGTGGTTTTTCTTTTCACCAGTGAGACGGGCAACAGCTGATTGCCCTTCACCGCCTGGCCCT
GAGAGAGTTGCAGCAAGCGGTCCACGCTGGTTTGCCCCAGCAGGCGAAAATCCTGTTTGATGGTGGTT
AACGGCGGGATATAACATGAGCTGTCTTCGGTATCGTCGTATCCCACTACCGAGATGTCCGCACCAAC
GCGCAGCCCGGACTCGGTAATGGCGCGCATTGCGCCCAGCGCCATCTGATCGTTGGCAACCAGCATCG
CAGTGGGAACGATGCCCTCATTCAGCATTTGCATGGTTTGTTGAAAACCGGACATGGCACTCCAGTCG
CCTTCCCGTTCCGCTATCGGCTGAATTTGATTGCGAGTGAGATATTTATGCCAGCCAGCCAGACGCAG
ACGCGCCGAGACAGAACTTAATGGGCCCGCTAACAGCGCGATTTGCTGGTGACCCAATGCGACCAGAT
GCTCCACGCCCAGTCGCGTACCGTCTTCATGGGAGAAAATAATACTGTTGATGGGTGTCTGGTCAGAG
ACATCAAGAAATAACGCCGGAACATTAGTGCAGGCAGCTTCCACAGCAATGGCATCCTGGTCATCCAG
CGGATAGTTAATGATCAGCCCACTGACGCGTTGCGCGAGAAGATTGTGCACCGCCGCTTTACAGGCTT
CGACGCCGCTTCGTTCTACCATCGACACCACCACGCTGGCACCCAGTTGATCGGCGCGAGATTTAATC
GCCGCGACAATTTGCGACGGCGCGTGCAGGGCCAGACTGGAGGTGGCAACGCCAATCAGCAACGACTG
TTTGCCCGCCAGTTGTTGTGCCACGCGGTTGGGAATGTAATTCAGCTCCGCCATCGCCGCTTCCACTT
TTTCCCGCGTTTTCGCAGAAACGTGGCTGGCCTGGTTCACCACGCGGGAAACGGTCTGATAAGAGACA
CCGGCATACTCTGCGACATCGTATAACGTTACTGGTTTCACATTCACCACCCTGAATTGACTCTCTTC
CGGGCGCTATCATGCCATACCGCGAAAGGTTTTGCGCCATTCGATGGTGTCCGGGATCTCGACGCTCT
CCCTTATGCGACTCCTGCATTAGGAAGCAGCCCAGTAGTAGGTTGAGGCCGTTGAGCACCGCCGCCGC
AAGGAATGGTGCATGCAAGGAGATGGCGCCCAACAGTCCCCCGGCCACGGGGCCTGCCACCATACCCA
CGCCGAAACAAGCGCTCATGAGCCCGAAGTGGCGAGCCCGATCTTCCCCATCGGTGATGTCGGCGATA
TAGGCGCCAGCAACCGCACCTGTGGCGCCGGTGATGCCGGCCACGATGCGTCCGGCGTAGAGGATCGA
GATCGATCTCGATCCCGCGAAATTAATACGACTCACTATAGGGGAATTGTGAGCGGATAACAATTCCC
CTCTAGAAATAATTTTGTTTAACTTTAAGAAGGAGATATACATATGCACCATCATCATCATCATTCTT
CTGGTCTGGTGCCACGCGGTTCTGGTATGAAAGAAACCGCTGCTGCTAAATTCGAACGCCAGCACATG
GACAGCCCAGATCTGGGTACCGACGACGACGACAAGGCCATGGCTGATATCGGATCCATGGCTACAGA
GGAAAATGTAAAAAGAAGGACGCATAACTGCCTGGAGCGTCAGCGTCGTTGTGAACTGAAGCGCTCTT
TTTTCGCCCTGCGCGATCAGATTCCGGAATTGGAGAACAACGAGAAGGCTCCGAAAGTTGTTATTCTG
AAGAAGGCGACCGCATACATCCTCTCCGTGCAAGCGGAAACCCAGAAACTGATCAGCGAGATCGACCT
GCTGCGTAAACAGAATGAACAATTAAAGCACAAGTTGGAGCAACTGCGTAATAGCGCGGCTCCGGCAG
CGAAACGCGTGAAATTGGACTAAGGACTCGAGCACCACCACCACCACCACTGAGATCCGGCTGCTAAC

AAAGCCCGAAAGGAAGCTGAGTTGGCTGCTGCCACCGCTGAGCAATAACTAGCATAACCCCTTGGGGC
CTCTAAACGGGTCTTGAGGGGTTTTTTGCTGAAAGGAGGAACTATATCCGGAT

pET30a with NucleoMyc 17

TGGCGAATGGGACGCGCCCTGTAGCGGCGCATTAAGCGCGGCGGGTGTGGTGGTTACGCGCAGCGTGA
CCGCTACACTTGCCAGCGCCCTAGCGCCCGCTCCTTTCGCTTTCTTCCCTTCCTTTCTCGCCACGTTC
GCCGGCTTTCCCCGTCAAGCTCTAAATCGGGGGCTCCCTTTAGGGTTCCGATTTAGTGCTTTACGGCA
CCTCGACCCCAAAAAACTTGATTAGGGTGATGGTTCACGTAGTGGGCCATCGCCCTGATAGACGGTTT
TTCGCCCTTTGACGTTGGAGTCCACGTTCTTTAATAGTGGACTCTTGTTCCAAACTGGAACAACACTC
AACCCTATCTCGGTCTATTCTTTTGATTTATAAGGGATTTTGCCGATTTCGGCCTATTGGTTAAAAAA
TGAGCTGATTTAACAAAAATTTAACGCGAATTTTAACAAAATATTAACGTTTACAATTTCAGGTGGCA
CTTTTCGGGGAAATGTGCGCGGAACCCCTATTTGTTTATTTTTCTAAATACATTCAAATATGTATCCG
CTCATGAATTAATTCTTAGAAAAACTCATCGAGCATCAAATGAAACTGCAATTTATTCATATCAGGAT
TATCAATACCATATTTTTGAAAAAGCCGTTTCTGTAATGAAGGAGAAAACTCACCGAGGCAGTTCCAT
AGGATGGCAAGATCCTGGTATCGGTCTGCGATTCCGACTCGTCCAACATCAATACAACCTATTAATTT
CCCCTCGTCAAAAATAAGGTTATCAAGTGAGAAATCACCATGAGTGACGACTGAATCCGGTGAGAATG
GCAAAAGTTTATGCATTTCTTTCCAGACTTGTTCAACAGGCCAGCCATTACGCTCGTCATCAAAATCA
CTCGCATCAACCAAACCGTTATTCATTCGTGATTGCGCCTGAGCGAGACGAAATACGCGATCGCTGTT
AAAAGGACAATTACAAACAGGAATCGAATGCAACCGGCGCAGGAACACTGCCAGCGCATCAACAATAT
TTTCACCTGAATCAGGATATTCTTCTAATACCTGGAATGCTGTTTTCCCGGGGATCGCAGTGGTGAGT
AACCATGCATCATCAGGAGTACGGATAAAATGCTTGATGGTCGGAAGAGGCATAAATTCCGTCAGCCA
GTTTAGTCTGACCATCTCATCTGTAACATCATTGGCAACGCTACCTTTGCCATGTTTCAGAAACAACT
CTGGCGCATCGGGCTTCCCATACAATCGATAGATTGTCGCACCTGATTGCCCGACATTATCGCGAGCC
CATTTATACCCATATAAATCAGCATCCATGTTGGAATTTAATCGCGGCCTAGAGCAAGACGTTTCCCG
TTGAATATGGCTCATAACACCCCTTGTATTACTGTTTATGTAAGCAGACAGTTTTATTGTTCATGACC
AAAATCCCTTAACGTGAGTTTTCGTTCCACTGAGCGTCAGACCCCGTAGAAAAGATCAAAGGATCTTC
TTGAGATCCTTTTTTTCTGCGCGTAATCTGCTGCTTGCAAACAAAAAAACCACCGCTACCAGCGGTGG
TTTGTTTGCCGGATCAAGAGCTACCAACTCTTTTTCCGAAGGTAACTGGCTTCAGCAGAGCGCAGATA
CCAAATACTGTCCTTCTAGTGTAGCCGTAGTTAGGCCACCACTTCAAGAACTCTGTAGCACCGCCTAC
ATACCTCGCTCTGCTAATCCTGTTACCAGTGGCTGCTGCCAGTGGCGATAAGTCGTGTCTTACCGGGT
TGGACTCAAGACGATAGTTACCGGATAAGGCGCAGCGGTCGGGCTGAACGGGGGGTTCGTGCACACAG
CCCAGCTTGGAGCGAACGACCTACACCGAACTGAGATACCTACAGCGTGAGCTATGAGAAAGCGCCAC
GCTTCCCGAAGGGAGAAAGGCGGACAGGTATCCGGTAAGCGGCAGGGTCGGAACAGGAGAGCGCACGA
GGGAGCTTCCAGGGGGAAACGCCTGGTATCTTTATAGTCCTGTCGGGTTTCGCCACCTCTGACTTGAG
CGTCGATTTTTGTGATGCTCGTCAGGGGGGCGGAGCCTATGGAAAAACGCCAGCAACGCGGCCTTTTT
ACGGTTCCTGGCCTTTTGCTGGCCTTTTGCTCACATGTTCTTTCCTGCGTTATCCCCTGATTCTGTGG
ATAACCGTATTACCGCCTTTGAGTGAGCTGATACCGCTCGCCGCAGCCGAACGACCGAGCGCAGCGAG
TCAGTGAGCGAGGAAGCGGAAGAGCGCCTGATGCGGTATTTTCTCCTTACGCATCTGTGCGGTATTTC
ACACCGCATATATGGTGCACTCTCAGTACAATCTGCTCTGATGCCGCATAGTTAAGCCAGTATACACT
CCGCTATCGCTACGTGACTGGGTCATGGCTGCGCCCCGACACCCGCCAACACCCGCTGACGCGCCCTG
ACGGGCTTGTCTGCTCCCGGCATCCGCTTACAGACAAGCTGTGACCGTCTCCGGGAGCTGCATGTGTC
AGAGGTTTTCACCGTCATCACCGAAACGCGCGAGGCAGCTGCGGTAAAGCTCATCAGCGTGGTCGTGA
AGCGATTCACAGATGTCTGCCTGTTCATCCGCGTCCAGCTCGTTGAGTTTCTCCAGAAGCGTTAATGT
CTGGCTTCTGATAAAGCGGGCCATGTTAAGGGCGGTTTTTTCCTGTTTGGTCACTGATGCCTCCGTGT
AAGGGGGATTTCTGTTCATGGGGGTAATGATACCGATGAAACGAGAGAGGATGCTCACGATACGGGTT
ACTGATGATGAACATGCCCGGTTACTGGAACGTTGTGAGGGTAAACAACTGGCGGTATGGATGCGGCG
GGACCAGAGAAAAATCACTCAGGGTCAATGCCAGCGCTTCGTTAATACAGATGTAGGTGTTCCACAGG
GTAGCCAGCAGCATCCTGCGATGCAGATCCGGAACATAATGGTGCAGGGCGCTGACTTCCGCGTTTCC
AGACTTTACGAAACACGGAAACCGAAGACCATTCATGTTGTTGCTCAGGTCGCAGACGTTTTGCAGCA
GCAGTCGCTTCACGTTCGCTCGCGTATCGGTGATTCATTCTGCTAACCAGTAAGGCAACCCCGCCAGC
CTAGCCGGGTCCTCAACGACAGGAGCACGATCATGCGCACCCGTGGGGCCGCCATGCCGGCGATAATG
GCCTGCTTCTCGCCGAAACGTTTGGTGGCGGGACCAGTGACGAAGGCTTGAGCGAGGGCGTGCAAGAT

TCCGAATACCGCAAGCGACAGGCCGATCATCGTCGCGCTCCAGCGAAAGCGGTCCTCGCCGAAAATGA
CCCAGAGCGCTGCCGGCACCTGTCCTACGAGTTGCATGATAAAGAAGACAGTCATAAGTGCGGCGACG
ATAGTCATGCCCCGCGCCCACCGGAAGGAGCTGACTGGGTTGAAGGCTCTCAAGGGCATCGGTCGAGA
TCCCGGTGCCTAATGAGTGAGCTAACTTACATTAATTGCGTTGCGCTCACTGCCCGCTTTCCAGTCGG
GAAACCTGTCGTGCCAGCTGCATTAATGAATCGGCCAACGCGCGGGGAGAGGCGGTTTGCGTATTGGG
CGCCAGGGTGGTTTTTCTTTTCACCAGTGAGACGGGCAACAGCTGATTGCCCTTCACCGCCTGGCCCT
GAGAGAGTTGCAGCAAGCGGTCCACGCTGGTTTGCCCCAGCAGGCGAAAATCCTGTTTGATGGTGGTT
AACGGCGGGATATAACATGAGCTGTCTTCGGTATCGTCGTATCCCACTACCGAGATGTCCGCACCAAC
GCGCAGCCCGGACTCGGTAATGGCGCGCATTGCGCCCAGCGCCATCTGATCGTTGGCAACCAGCATCG
CAGTGGGAACGATGCCCTCATTCAGCATTTGCATGGTTTGTTGAAAACCGGACATGGCACTCCAGTCG
CCTTCCCGTTCCGCTATCGGCTGAATTTGATTGCGAGTGAGATATTTATGCCAGCCAGCCAGACGCAG
ACGCGCCGAGACAGAACTTAATGGGCCCGCTAACAGCGCGATTTGCTGGTGACCCAATGCGACCAGAT
GCTCCACGCCCAGTCGCGTACCGTCTTCATGGGAGAAAATAATACTGTTGATGGGTGTCTGGTCAGAG
ACATCAAGAAATAACGCCGGAACATTAGTGCAGGCAGCTTCCACAGCAATGGCATCCTGGTCATCCAG
CGGATAGTTAATGATCAGCCCACTGACGCGTTGCGCGAGAAGATTGTGCACCGCCGCTTTACAGGCTT
CGACGCCGCTTCGTTCTACCATCGACACCACCACGCTGGCACCCAGTTGATCGGCGCGAGATTTAATC
GCCGCGACAATTTGCGACGGCGCGTGCAGGGCCAGACTGGAGGTGGCAACGCCAATCAGCAACGACTG
TTTGCCCGCCAGTTGTTGTGCCACGCGGTTGGGAATGTAATTCAGCTCCGCCATCGCCGCTTCCACTT
TTTCCCGCGTTTTCGCAGAAACGTGGCTGGCCTGGTTCACCACGCGGGAAACGGTCTGATAAGAGACA
CCGGCATACTCTGCGACATCGTATAACGTTACTGGTTTCACATTCACCACCCTGAATTGACTCTCTTC
CGGGCGCTATCATGCCATACCGCGAAAGGTTTTGCGCCATTCGATGGTGTCCGGGATCTCGACGCTCT
CCCTTATGCGACTCCTGCATTAGGAAGCAGCCCAGTAGTAGGTTGAGGCCGTTGAGCACCGCCGCCGC
AAGGAATGGTGCATGCAAGGAGATGGCGCCCAACAGTCCCCCGGCCACGGGGCCTGCCACCATACCCA
CGCCGAAACAAGCGCTCATGAGCCCGAAGTGGCGAGCCCGATCTTCCCCATCGGTGATGTCGGCGATA
TAGGCGCCAGCAACCGCACCTGTGGCGCCGGTGATGCCGGCCACGATGCGTCCGGCGTAGAGGATCGA
GATCGATCTCGATCCCGCGAAATTAATACGACTCACTATAGGGGAATTGTGAGCGGATAACAATTCCC
CTCTAGAAATAATTTTGTTTAACTTTAAGAAGGAGATATACATATGCACCATCATCATCATCATTCTT
CTGGTCTGGTGCCACGCGGTTCTGGTATGAAAGAAACCGCTGCTGCTAAATTCGAACGCCAGCACATG
GACAGCCCAGATCTGGGTACCGACGACGACGACAAGGCCATGGCTGATATCGGATCCCCCGCTGCAAA
GAGGGTAAAACTAGATATGGCGACGGAAGAGAATGTTAAACGTCGTACCCATAACTGCCTGGAGCGCC
AGCGTCGTTGTGAATTAAAGCGCTCCTTCTTTGCCCTGAGAGACCAAATTCCGGAATTGGAGAACAAC
GAGAAAGCTCCGAAGGTGGTGATCCTGAAGAAGGCGACCGCATACATCTTGTCTGTTCAGGCTGAAAC
CCAGAAATTGATCAGCGAAATTGATCTGCTGCGTAAACAGAATGAGCAACTGAAGCACAAACTGGAGC
AACTGCGCAACAGCGCGGCGTAAGGACTCGAGCACCACCACCACCACCACTGAGATCCGGCTGCTAAC
AAAGCCCGAAAGGAAGCTGAGTTGGCTGCTGCCACCGCTGAGCAATAACTAGCATAACCCCTTGGGGC
CTCTAAACGGGTCTTGAGGGGTTTTTTGCTGAAAGGAGGAACTATATCCGGAT

RNA-seq genes up genes down

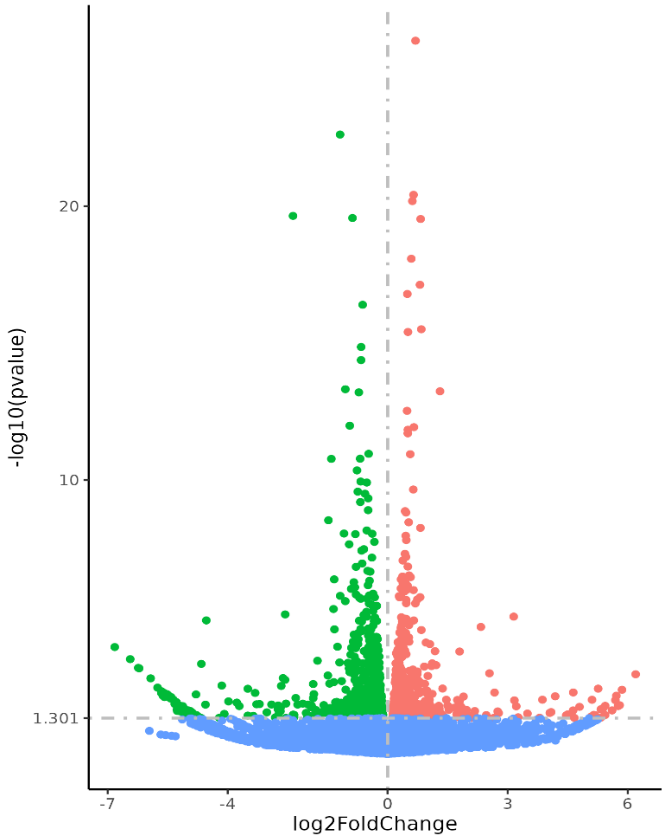

Figure S22. Volcano plot of differentially expressed genes.

Genes down log2FoldChange < -0.5

gene_name	log2FoldChange	pvalue
AC133644.3	-6.823077314	0.00012648
MAB21L3	-6.436453731	0.00034981
AL627230.4	-6.234855054	0.00072943
LINC00601	-6.21271341	0.00075696
SSX1	-5.925706604	0.00175689
AL159169.2	-5.752963154	0.00384909
AC026367.3	-5.657104079	0.00607032
AP003469.4	-5.655896714	0.00519773
AC024243.1	-5.619709486	0.00754697
MYH3	-5.601539684	0.00588883
CATIP	-5.579758467	0.00849843
AC009812.4	-5.568463514	0.00625731
KLB	-5.509164211	0.00675599
DNAH10	-5.474445244	0.00758093
ZNF890P	-5.437053093	0.00915722
AL022393.1	-5.427427385	0.00952639
LYG1	-5.419397055	0.00912231
APOB	-5.413533758	0.00850945
AL158166.1	-5.359135472	0.01053074
AC116036.2	-5.322642817	0.0151583
AL121936.1	-5.279125534	0.01311465
KRT8P26	-5.269954006	0.02458203
ATP4A	-5.260596577	0.0272528
AC013565.1	-5.21740305	0.01733951
BEST4	-5.196637809	0.02789982
NLRP10	-5.191345313	0.01944899
ARL4AP5	-5.189825031	0.01787569
SLC5A4-AS1	-5.154904559	0.01817848
TRIM17	-5.124026933	0.01815877
AC007228.1	-5.111284195	0.02127711
HNRNPUP1	-5.095740113	0.02148121
ARHGAP11B	-5.088435258	0.03565102
AFAP1-AS1	-5.066082169	0.03646272
AC243654.3	-5.065913631	0.03964371
TMEM100	-5.035051743	0.04020139
MYO5BP2	-5.028990802	0.02687124
METTL7B	-5.005455357	0.02728866
AC244153.1	-5.004121018	0.0257989
KCNH4	-4.997528301	0.04410829
S1PR1	-4.996579971	0.04240506
RNVU1-6	-4.987472654	0.03093016
AC007336.1	-4.971821235	0.04407257
AC132008.1	-4.969844241	0.02664209
LINC01058	-4.946236412	0.03207616
OR6A2	-4.944691069	0.03051809
PTPRO	-4.942328998	0.0271397

FRMD4A	-4.941219091	0.02791558
NUTM2E	-4.919019052	0.04814238
TRPM6	-4.911745016	0.02992029
C6orf58	-4.910031359	0.02960241
GRAP2	-4.910031359	0.02960241
TEX37	-4.900769569	0.03661038
SNORA26	-4.894719738	0.03131858
AL731563.3	-4.882472973	0.03178364
AC037487.3	-4.880273232	0.03449713
CD36	-4.833996658	0.03705016
LENEP	-4.820281111	0.03916944
APOL3	-4.817807404	0.0393379
AL356740.3	-4.791342018	0.0068193
AC034243.1	-4.784117302	0.03854083
AC104596.1	-4.749965729	0.04217158
MNX1-AS2	-4.736027003	0.04635112
IMPDH1P4	-4.718001978	0.04741436
AL354892.3	-4.712851983	0.04719181
AC245100.1	-4.661379242	0.04986696
SMARCE1P3	-4.660012754	0.04941212
LHFPL5	-4.659033062	0.00052177
PTAFR	-4.554700298	0.01604995
FP236383.2	-4.533964221	1.34E-05
AC093326.1	-4.211940216	0.04866115
EDNRA	-4.147823648	0.00322558
OR2A7	-4.084415279	0.0350563
HBA2	-3.978797301	0.01225056
AC016588.2	-3.770669943	0.01357857
HLA-F	-3.709702521	0.01816173
AC018638.4	-3.497710483	0.04050155
TRAF1	-3.49679868	0.00423507
AL358777.1	-3.36034452	0.04118198
PI3	-3.313487504	0.00599925
LRRC34	-3.277347525	0.01557834
AC063952.1	-3.203396888	0.01531539
NXPH3	-3.019867211	0.03068907
CORIN	-2.903680568	0.01587982
USP3-AS1	-2.811444415	0.04725969
DNMT3L	-2.742015766	0.02364116
MEIG1	-2.741207086	0.03922512
RFTN2	-2.737043352	0.01725878
SERTAD4-AS1	-2.681949154	0.02797983
EFCAB6	-2.67398705	0.01997705
SPRY4	-2.661182485	0.00333368
FP236383.3	-2.615874772	0.00171948
NEUROG2	-2.566116345	0.00199282
SERTAD4	-2.562398901	8.13E-06
UNC5CL	-2.543017621	0.0488332

TSKS	-2.541864132	0.01469488
DKK1	-2.36619129	2.26E-20
FAM225A	-2.364882672	0.03740973
PHF24	-2.34071168	0.04620236
SMARCE1P5	-2.339120504	0.03802661
SAA1	-2.298231869	0.00916015
GEM	-2.252112681	0.01596017
MT1F	-2.121566252	0.01474996
AC090517.2	-2.03877191	0.03227104
LINC01004	-2.007074457	0.04512284
AC097468.3	-1.993911805	0.03935994
CYB5R2	-1.990214832	0.01593475
TEX36	-1.914547757	0.01926329
SPOCK1	-1.904530103	0.01739968
AC103706.1	-1.878822262	0.02102861
HRAT92	-1.862644578	0.00933139
ETV4	-1.862197462	0.00283995
DNAH1	-1.845524488	0.00663056
AF196972.1	-1.841235478	0.03963697
PRDM13	-1.819178448	0.04962717
ANK1	-1.790071583	0.02543068
NDUFV2	-1.788010485	0.03091798
AC005696.4	-1.777128225	0.04682497
AL139384.1	-1.77658303	0.0370108
STC1	-1.754440305	0.00039977
TCF4	-1.730675177	0.03877902
C5	-1.692636354	0.03757608
CCSER1	-1.632216673	0.03022917
GPR3	-1.59746016	0.01665819
LGALS9C	-1.574859741	0.03206643
ZC3H12B	-1.571278576	0.01975488
GVQW2	-1.540745979	0.03114012
TCF7	-1.495753308	0.001386
ETV5	-1.481304541	2.97E-09
MYEOV	-1.453314771	0.00273712
AC025287.2	-1.437654984	0.02134603
IGSF10	-1.408409342	0.04277855
SEMA3D	-1.407768198	1.69E-11
CHL1	-1.354739749	5.16E-06
REN	-1.346885889	0.02202237
TRMT9B	-1.346530626	0.00242486
THBS1	-1.33747151	4.27E-07
ARG2	-1.331754821	2.85E-05
AC004231.1	-1.326404561	0.0307798
AL391988.1	-1.30790627	0.0131902
SCARA5	-1.291192146	0.04880855
AL359504.2	-1.274325303	0.01274497
CYP19A1	-1.254136786	0.00012303
KIAA1324	-1.217152549	0.02384015

ALG1L9P	-1.212532459	0.03579732
LINC01132	-1.202953527	0.00046359
BEX2	-1.19414841	0.03272788
DUSP4	-1.190556284	2.41E-23
VIT	-1.187502509	1.71E-06
SCNN1A	-1.186244739	0.02917604
NDUFA4L2	-1.181179037	0.01968206
SYPL2	-1.165019961	0.013662
CT45A5	-1.153966209	0.02511605
FOSL1	-1.150045048	0.00221869
NECAB2	-1.129178604	0.02974641
SLC7A11-AS1	-1.11837856	0.02024614
TRIB2	-1.111374192	0.0207541
EDN1	-1.101721716	0.04812593
ADGRG3	-1.091666218	9.05E-09
HIST1H1E	-1.089409432	0.04772062
ZNF585B	-1.087828363	0.01459835
PRKCH	-1.087577977	0.01813899
LGALS9B	-1.071226701	0.02513551
VEGFC	-1.065152142	0.02444679
NEDD9	-1.059175102	2.67E-06
ID1	-1.058081262	4.85E-14
FAM222A	-1.049006539	0.00456254
PALM2	-1.028948377	0.0334626
SAMD4A	-1.014985205	0.01021362
AC097478.1	-0.994912859	0.00047942
SLC16A14	-0.981443446	0.02310357
CA9	-0.977474357	0.00053481
LAMP3	-0.965169765	0.00076239
FGF18	-0.964617077	0.01553523
ADAM12	-0.963004046	2.23E-08
AC245100.3	-0.962330039	0.02348515
RPS15AP1	-0.960031021	0.03530569
CT45A1	-0.947899817	0.0010485
IER3	-0.947200346	1.04E-12
ATP8B1	-0.935905603	0.02923081
DKK3	-0.926364878	0.01925602
N4BP3	-0.923817532	0.00023947
MASP1	-0.917256279	0.00673221
SPRED2	-0.912096669	9.49E-07
ABCC3	-0.906556844	0.00013532
SERPINE2	-0.87995833	2.70E-20
LONRF2	-0.871736432	0.0433989
NTN4	-0.864362455	0.0107008
PTP4A1	-0.864196675	8.03E-05
MAFF	-0.854361485	0.00047982
MAPK8IP3	-0.85197796	0.01748287
THSD4	-0.846397089	5.43E-07

SPICE1	-0.845881853	0.03928169
HR	-0.84318998	4.44E-05
UBASH3B	-0.841226827	0.00126363
ZEB1-AS1	-0.830213327	0.01867664
TEX19	-0.827769414	0.01549416
SPCS2P4	-0.827494155	0.00028619
UNC5B	-0.822963574	0.00202349
NPNT	-0.820264391	8.30E-07
ECM2	-0.814288657	0.02098935
CAV1	-0.806917432	9.53E-09
ALPP	-0.805748229	0.00057719
PIK3R1	-0.803038871	0.00071041
AC107027.3	-0.802023146	0.0180124
NPAS2	-0.801015815	5.55E-05
SH3RF3	-0.798949044	0.00013877
ARHGAP26	-0.797993991	0.00471067
NDRG1	-0.791961217	0.01129907
TFPI2	-0.791489108	1.49E-07
NRP2	-0.791002518	1.53E-06
IL11	-0.790111287	0.04689674
HMGA2	-0.789644153	0.02570569
COL14A1	-0.783513105	0.0007978
TGFB2	-0.782667132	0.00664278
EFHD1	-0.781240377	0.00199093
C15orf65	-0.778610841	0.03110483
COL18A1	-0.778374321	0.00096016
SH3RF3-AS1	-0.77400416	0.03531098
TNFRSF11B	-0.770052743	0.00041679
TMTC2	-0.768950092	0.00285047
SLC16A6	-0.766725537	4.46E-11
ID3	-0.747273671	2.67E-10
EPHA5	-0.741473872	0.02167319
FGF13	-0.741380587	9.87E-05
PADI3	-0.739206657	0.01023022
IRS1	-0.738517138	3.92E-05
BCAR3	-0.732088071	6.56E-05
DLGAP1-AS2	-0.72948086	0.02804867
SOX18	-0.725815108	0.00244953
SPRED1	-0.721260602	6.30E-14
AC092115.1	-0.718698915	0.03405252
NOMO3	-0.712643746	0.00910663
PDGFC	-0.710909101	1.38E-05
PLAUR	-0.707207738	1.98E-06
PLXNA3	-0.698748524	0.00233131
AKR1B10	-0.693328606	0.02229197
TNFRSF12A	-0.687821575	1.67E-11
PTGS2	-0.683663698	6.41E-10
GUCY1A2	-0.682418613	0.01558111

MALAT1	-0.677010516	2.77E-05
CXCR4	-0.675435017	1.14E-10
ITGA2	-0.672653964	2.18E-06
CA12	-0.668261813	0.04099141
ATOH8	-0.667443808	0.00767001
PTGES	-0.66548163	3.38E-05
AMIGO2	-0.665188952	0.01888355
FBN2	-0.66473746	4.18E-15
DMD	-0.663038917	1.39E-15
PHOSPHO2	-0.662613331	0.03264879
LINC01138	-0.656342577	0.01225939
RAPGEF4	-0.656219914	0.00416593
FAM129A	-0.65267543	3.80E-08
WFDC1	-0.651062798	0.00092378
PAPPA	-0.649646582	1.49E-05
NEBL	-0.640150122	0.0062612
TGM2	-0.63779441	1.12E-07
RNF122	-0.636245406	0.02973216
DCUN1D3	-0.628037746	0.00093293
SCUBE1	-0.626023584	0.00049968
CCND1	-0.625452878	3.98E-17
HIST1H2BH	-0.624926258	0.0330412
AKNA	-0.623512189	0.00182172
COL4A6	-0.617090904	0.00218917
LGSN	-0.613095266	0.00153975
TFEB	-0.613007019	0.0407323
SSTR1	-0.608396723	0.00101418
ZNF738	-0.605635169	0.03748603
LINC02593	-0.60504708	0.04445754
IER5L	-0.596300731	8.42E-06
COL12A1	-0.595806624	3.37E-08
PLEK2	-0.595274502	0.00999557
VDR	-0.590398748	0.00017803
PLAU	-0.590014953	0.00516022
CD163L1	-0.588550445	0.04643405
PPP1R3G	-0.584926141	0.02929887
AC245041.1	-0.579660776	0.03211238
RUNX3	-0.574156089	0.00942457
SLFN12	-0.57394527	0.01524621
LINC01503	-0.573822127	0.02318174
ZNF561-AS1	-0.571258591	0.01346133
INA	-0.570495335	0.0110737
SULF2	-0.569043166	0.00825304
ATP6V0A4	-0.568508736	0.00658665
ST3GAL1	-0.567154423	3.18E-10
SLC16A5	-0.564460505	0.00163996
ATP8A1	-0.56311941	0.02480951
NPTXR	-0.554103977	0.01654421
Z95115.1	-0.548897827	0.01933324

OLA1P1	-0.546487637	0.01838625
CNTNAP3	-0.540642306	0.01140681
GPR156	-0.539477581	0.03042898
SMAD6	-0.533167741	0.00410484
AC004057.1	-0.529280376	6.53E-06
IGSF1	-0.526948946	0.04305741
GAREM1	-0.526430112	0.0411974
SLFN5	-0.524586975	0.00016819
SMAD7	-0.523858852	0.03054824
PCDH7	-0.523240679	1.24E-10
STXBP5	-0.523029153	6.96E-09
PLK2	-0.522831733	9.87E-06
NOV	-0.517554074	2.14E-06
CASC10	-0.513678979	0.04214191
GLP2R	-0.512517235	0.00025492
AC016717.2	-0.511450687	0.00053227
ADORA1	-0.509376232	0.0388819
NDUFB8	-0.507420474	0.00249694
MAP2	-0.504274576	0.00015021
RERG	-0.501240787	2.09E-07

Genes up log2FoldChange > 0.5

gene_name	log2FoldChange	pvalue
AC016877.3	6.198969865	0.001249571
AC124016.2	5.858680073	0.004612042
P2RY1	5.798017357	0.016835439
ODF3L2	5.761452902	0.018143708
ST3GAL1P1	5.716900232	0.007608729
FAM157C	5.693610454	0.011025525
MRVI1	5.609524295	0.026062097
PADI2	5.437470597	0.017760771
TRPC5OS	5.435257867	0.03855444
AC007663.3	5.383202577	0.02186894
MIR3682	5.330851811	0.023796087
AC097381.1	5.330851811	0.023796087
AC083906.5	5.328290596	0.047870654
AC027088.3	5.261193465	0.004198084
AC004024.1	5.182200142	0.038790758
AC008875.1	5.152184073	0.038040476
AC080013.3	5.108228495	0.010676676
GPR87	5.090588702	0.043331132
RPS12P31	4.794940574	0.019356479
AC008555.8	4.665539288	0.048741401
AC091982.1	4.638823887	0.005761081
AL359715.4	4.635233867	0.026177057
SEC22B3	4.499944447	0.03668791
FAM87B	4.46340165	0.039477358
AC020658.5	4.186911721	0.007963957
KCNK9	4.086160766	0.030101428
TBILA	4.052551558	0.032233001
AC090772.1	3.868142971	0.010610985
AC129492.4	3.797752519	0.048234238
CPB2-AS1	3.493638704	0.034025642
AC011498.1	3.261790083	0.046438599
CLPSL1	3.213916725	0.018469174
C10orf111	3.167095898	0.011204546
C9orf152	3.150446074	9.81E-06
MAB21L4	2.773730677	0.032576517
APBA2	2.750037089	0.028418203
IKBKGP1	2.671783233	0.00580805
AC234775.3	2.650611838	0.049020667
TM4SF20	2.544922983	0.001150053
U73166.1	2.463673703	0.038796588
FGB	2.329497515	2.33E-05
ACSBG2	2.177342091	0.027483551
LINC01556	2.175433603	0.042342955
AL139156.2	2.104028396	0.048652432
SEMA5B	2.066030294	0.048456969
SNORA73B	2.044463439	0.041136649

XKR5	1.997016938	0.042766732
CHRM1	1.980070847	0.03885515
AC005839.1	1.972579289	0.025080333
AC138207.4	1.895353648	0.00812957
SIAH2-AS1	1.85447307	0.030733046
AC087071.2	1.85341201	0.021398527
ELANE	1.846230573	0.02786779
RASGRP2	1.831594977	0.01894673
UGT3A1	1.797498328	0.000184357
TUFMP1	1.791709304	0.012302949
NKX3-2	1.789586662	0.04743199
GACAT2	1.78444306	0.020723803
AP000357.2	1.779484144	0.014606382
GNRH1	1.753020735	0.021842169
AL122035.1	1.741272339	0.010870148
ARMC10P1	1.73808095	0.021272606
GPR20	1.721912068	0.0357928
TMEM63C	1.657455777	0.020846018
GOLGA8K	1.529480711	0.024819371
CRYBG2	1.498911752	0.017494135
CCL28	1.465463487	0.010627082
PACSIN1	1.411407924	0.044331073
AC084880.1	1.408301113	0.044358277
MFNG	1.371969438	0.004153696
AC005332.4	1.355628101	0.036821425
SYTL3	1.313155164	0.031880982
FGG	1.307949099	5.77E-14
FAM174B	1.292417518	0.023054655
HGD	1.279161801	0.031715179
C6orf99	1.277764944	0.017709355
PTGES3P3	1.248391958	0.03992468
AC002467.1	1.20949968	0.000580426
SELENOP	1.183184865	0.000179392
SPDEF	1.170312273	0.049909636
TKTL1	1.165771859	0.043072264
GJD3	1.163825202	0.020807479
RRAD	1.163685861	0.025490419
THAP12P7	1.159829768	0.044635904
MISP3	1.114776866	0.006500566
GOLGA2P7	1.11304639	0.000499421
KLHL30	1.09406901	0.000628008
RASGRP3	1.055105314	9.90E-05
GOLGA8B	1.050599117	0.007514039
NEK10	1.043621624	0.010697374
TNNI2	1.042849004	0.014371244
MESP2	1.031709771	0.030809192
RASSF6	1.018970886	0.024810682
KLF2	1.003425406	0.001436611
YTHDF3-AS1	0.99532687	0.00645271

ALOX12	0.986714498	0.020528569
HPN	0.982357402	0.004914099
PLCD4	0.962205004	0.042689092
CALY	0.957135815	8.69E-05
NOXA1	0.942531081	0.012233381
LINC00634	0.940655339	0.021764565
ACTG2	0.936395512	0.011744836
ZNF17	0.926953189	0.002795615
AC091271.1	0.922210673	0.014921121
FGF19	0.91510036	0.000653103
FOXD3	0.910147766	0.020604475
GNG7	0.905664229	0.047981926
FGFBP1	0.885999029	0.0055185
LINC00322	0.879369997	0.014672343
AL451165.2	0.877467773	0.04185975
BMF	0.841300963	3.03E-05
ENO3	0.840519991	3.12E-16
LINC00346	0.838975314	0.005249788
ARHGAP5-AS1	0.834451095	0.044927809
ELF3-AS1	0.830436539	0.024720429
ABCA1	0.825698003	0.00255324
ARHGEF10L	0.824256062	2.93E-20
MYBPC2	0.819390023	5.63E-09
GOLGA8N	0.811709839	1.91E-06
BCYRN1	0.80825435	0.049752998
BDKRB2	0.807283556	7.40E-18
DUSP13	0.793867176	0.006164379
TIGAR	0.780646658	0.0433927
ZNF438	0.77965907	0.037552895
FBXO32	0.76625051	0.00209722
GOLGA8Q	0.761390204	0.01537715
NBPF19	0.752288562	0.002061636
RELB	0.745718083	0.004004358
AL391422.4	0.7417235	0.000991792
KCNJ18	0.740816754	2.31E-06
DEFB1	0.738163588	0.001239005
AC092135.3	0.731851963	0.001250079
SLC6A19	0.717680585	0.049550925
HMGCS1	0.695550576	9.03E-27
CYP51A1	0.691968092	3.23E-06
CPLANE2	0.690019068	0.007212481
NUDT13	0.688053496	0.023213391
GDF6	0.677713505	0.008401143
C21orf58	0.663106202	0.002934749
TAGLN2P1	0.66179938	0.040754786
SPR	0.655407079	1.17E-12
MTLN	0.651953113	1.35E-05
MSMO1	0.646560868	3.83E-21

ADM	0.644705581	1.05E-06
ANO2	0.640253318	2.23E-10
FABP3	0.638873098	0.012644539
NANOS1	0.63155074	0.011359814
ERFE	0.630775195	0.000838845
PXMP4	0.627450384	0.011838815
AK7	0.619805245	0.031406314
INSIG1	0.617476371	6.52E-21
CARD6	0.616569423	0.003183003
ARVCF	0.592819135	0.001531398
CTH	0.590837883	8.29E-19
USP2-AS1	0.586160163	0.028370802
HES6	0.584560517	0.00350618
TENT5B	0.581787988	0.002263686
NBPF26	0.579486261	0.015132009
AC111200.2	0.57780321	0.029498622
ARSD	0.574681954	0.015389304
TRGC1	0.572283512	3.54E-07
NPL	0.570939385	0.025994632
LMTK3	0.567150369	0.03896721
LRRCC1	0.563894721	0.005970236
CHST13	0.563664513	1.14E-11
AATK	0.554566519	0.007754715
CREB3L1	0.55299109	0.003560243
FAM69B	0.545833581	0.008411796
RAPGEF6	0.545521558	0.006052248
NDUFV2P1	0.543850641	0.032594658
ACOT1	0.542034978	0.000935374
CHRD	0.540701874	0.048384164
PHLDB3	0.539701794	0.038688798
PTP4A3	0.533592494	8.11E-05
MVD	0.533527057	3.06E-07
ANKRD33B	0.53136374	2.01E-05
TNNC1	0.529703833	0.002051072
OPTN	0.527926327	5.66E-05
TLR6	0.527246583	0.01850227
LSS	0.525471551	3.50E-09
AC027309.2	0.5218092	0.033926847
AC007537.1	0.510283044	0.014449119
TM4SF1	0.50667269	3.93E-16
KIAA1328	0.506534314	0.035226985
IDI1	0.505106203	1.46E-12
KCNK15	0.504069813	1.46E-07
FDPS	0.50305135	1.98E-12

Overview of all produced miniproteins

Protein	Sequence	Yield[1]	Molecular weight calculated	Molecular weight observed
Omomyc (**1**)	AMADIGSMATEENVKRRTHNVLERQRRNELKRSFFALRDQIPELENNEKAPKVVILKKATAYILSVQAETQKLISEIDLLRKQNEQLKHKLEQLRNSCA	13.7 mg/L 4.5 mg/L	11527.3	11526.6
ArgMYC (**2**)	AMADIGSMATEENVKRRTHNVLERQRRNELKRSFFALRDQIPELENNEKAPKVVILKKATAYILSVQAETRKLISEIRLLRKQNEQLKHKLERLRNSAA		11592.5	11591.8
ArtiMYC (**3**)	AMADIGSMATEENVKRRTHNVLERQRRNELKRSFFALRDQIPELENNEKAPKVVILKKATAYILSVKREIAALKREIAALKREIAALKRE	8 mg/L	10398.2	10397.6
Protein **4**	AMADIGSMATEENVKRRTHNCLERQRRCELKRSFFALRDQIPELENNEKAPKVVILKKATAYILSVQAETQKLISEIDLLRKQNEQLKHKLEQLRNSAA	20.9 mg/L 10.4 mg/L	11488.3	11487.7
Protein **5**	AMADIGSMATEENCKRRTHNCLERQRRNELKRSFFALRDQIPELENNEKAPKVVILKKATAYILSVQAETQKLISEIDLLRKQNEQLKHKLEQLRNSAA	6.2 mg/L	11503.3	11502.6

Protein **6**	AMADIGSMACEENCKRRTHNVLERQRRNELKRSFFALRDQIPELENNEKAPKVVILKKATAYILSVQAETQK LISEIDLLRKQNEQLKHKLEQLRNSAA	7.3 mg /L 2.3 mg /L	11501. 3	11500. 6
Protein **7**	AMADIGSMATEENCKRRCHNVLERQRRNELKRSFFALRDQIPELENNEKAPKVVILKKATAYILSVQAETQK LISEIDLLRKQNEQLKHKLEQLRNSAA	8 mg /L 4.4 mg /L	11501. 3	11500. 6
HeloM YC- 1421 (**8**)	AMADIGSMATEENVKRRTHNCLERQRRCELKRSFFALRDQIPELENNEKAPKVVILKKATAYILSVQAETQK LISEIDLLRKQNEQLKHKLEQLRNSAA (stapled with 1)	69 %	11666. 3	11665. 8
HeloM YC-714 (**9**)	AMADIGSMATEENCKRRTHNCLERQRRNELKRSFFALRDQIPELENNEKAPKVVILKKATAYILSVQAETQK LISEIDLLRKQNEQLKHKLEQLRNSAA(stapled with 1)	67 %	11681. 3	11680. 8
HeloM YC-37 (**10**)	AMADIGSMACEENCKRRTHNVLERQRRNELKRSFFALRDQIPELENNEKAPKVVILKKATAYILSVQAETQK LISEIDLLRKQNEQLKHKLEQLRNSAA (stapled with 2)	28 %	11603. 3	11602. 8
HeloM YC-711 (**11**)	AMADIGSMATEENCKRRCHNVLERQRRNELKRSFFALRDQIPELENNEKAPKVVILKKATAYILSVQAETQK LISEIDLLRKQNEQLKHKLEQLRNSAA (stapled with 2)	26 %	11603. 3	11602. 8
Omom yc-FITC (**12**)	AMADIGSMATEENVKRRTHNVLERQRRNELKRSFFALRDQIPELENNEKAPKVVILKKATAYILSVQAETQK LISEIDLLRKQNEQLKHKLEQLRNSCA*FITC	22 %	11916. 7	11916. 1

HeloMYC-FITC (**13**)	AMADIGSMATEENVKRRTHNCLERQRRCELKRSFFALRDQIPELENNEKAPKVVILKKATAYILSVQAETQKLISEIDLLRKQNEQLKHKLEQLRNSAA*FITC (stapled with 1)	43 %	12055.7	12055.6
Benzo MYC (**S1**)	AMADIGSMATEENVKRRTHNCLERQRRCELKRSFFALRDQIPELENNEKAPKVVILKKATAYILSVQAETQKLISEIDLLRKQNEQLKHKLEQLRNSAA (Cys-capped with Bz)	37 %	11668.5	11667.9
Nucleo MYC (**16**)	ADIGSMATEENVKRRTHNCLERQRRCELKRSFFALRDQIPELENNEKAPKVVILKKATAYILSVQAETQKLISEIDLLRKQNEQLKHKLEQLRNSAAPAAKRVKLD (stapled with 1)	60 &	12645.2	12645.3
Nucleo MYC (**17**)	ADIGSPAAKRVKLDMATEENVKRRTHNCLERQRRCELKRSFFALRDQIPELENNEKAPKVVILKKATAYILSVQAETQKLISEIDLLRKQNEQLKHKLEQLRNSAA (stapled with 1)	46 %	12645.2	12645.1

[1]First number represents yield of his-tagged protein, second number represents yield of tag-cleaved protein

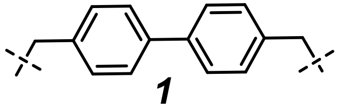

1

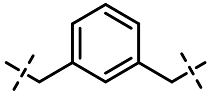

2

References

1. Jumper, J. *et al.* Highly accurate protein structure prediction with AlphaFold. *Nature* **596**, (2021).
2. Crone, N. S. A., Kros, A. & Boyle, A. L. Modulation of Coiled-Coil Binding Strength and Fusogenicity through Peptide Stapling. *Bioconjug. Chem.* **31**, 834–843 (2020).
